# Protocol for the MEXIRON study: a first-in-disease phase I–II investigation into the safety, tolerability and mechanistic proof-of-concept of extracorporeal iron depletion using the MEX-CD1 device in patients with transfusional iron overload

**DOI:** 10.1136/bmjopen-2026-121216

**Published:** 2026-10-27

**Authors:** Auguste Dargent, Mael Heiblig, Laurent Villeneuve, Marine Alves Ferreira, Maxime Bonjour, Francois Lux, Olivier Tillement, Bernard Allaouchiche

**Affiliations:** 1 Intensive Care, Lyon Sud Hospital, Hospices Civils de Lyon, Lyon, France; 2 VetAgro Sup, Campus Vétérinaire de Lyon, Marcy L’Etoile, France; 3 Department of Hematology, Centre Hospitalier Lyon Sud, Lyon, France; 4 Service Recherche & Épidémiologie Cliniques, Pôle Santé Publique, Hospices Civils de Lyon, Lyon, France; 5 CICLY, Université Claude Bernard Lyon 1, Lyon, France; 6 Service Recherche & Épidémiologie Cliniques, Hospices Civils de Lyon, Lyon, France; 7 Service de biostatistiques, Pôle Santé Publique, Hospices Civils de Lyon, Lyon, Auvergne-Rhône-Alpes, France; 8 Université Lyon 1, CNRS, ILM, UMR 5306, Villeurbanne, France, Lyon, France; 9 Department of Anesthesia and Intensive Care, Lyon Sud Teaching Hospital, Lyon, France

**Keywords:** Anaemia, Other metabolic, e.g. iron, porphyria, Dialysis, Blood bank & transfusion medicine

## Abstract

**Introduction:**

Secondary iron overload is a frequent and debilitating complication in patients who depend on chronic red blood cell transfusion support, including those with myelodysplastic syndromes (MDS), myelofibrosis and other haematological malignancies. As transferrin becomes saturated, iron appears in plasma in non-transferrin-bound forms whose redox-active fraction mediates organ toxicity. Oral and subcutaneous iron chelators are available, but a substantial subset of patients remains intolerant of or refractory to these therapies. This protocol presents a pilot clinical trial designed to evaluate the mechanistic feasibility and the safety of a novel approach: extracorporeal iron depletion using the MEX-CD1 medical device.

**Methods and analysis:**

The MEXIRON study is a prospective, open-label, single-arm, non-comparative, monocentric Phase I–II study sponsored by the Hospices Civils de Lyon. It aims to include 10 evaluable patients who are dependent on red blood cell transfusion in the context of a haematological malignancy or another condition of immuno-haematological origin, who have documented transfusional iron overload and who have a contraindication to or intolerance of conventional chelation. Participants will undergo a cycle of three extracorporeal iron depletion sessions over 1 week. The intervention uses the MEX-CD1 device, a functionalised biopolymer combining chitosan and DOTA-GA, coupled with a haemodialysis platform to selectively sequester plasma iron. The primary outcome is a mechanistic feasibility criterion: the mass of iron recovered in the dialysis effluent at the end of each session, quantified by inductively coupled plasma mass spectrometry. Secondary outcomes are safety and tolerance, the kinetics of iron removal, changes in serum ferritin and transferrin saturation, procedural feasibility, subsequent transfusion requirements and quality of life.

**Ethics and dissemination:**

The protocol has received approval from the Committee for the Protection of Persons (CPP Ouest III) and authorisation from the French National Agency for the Safety of Medicines (ANSM). Results will be disseminated through peer-reviewed publications.

**Trial registration number:**

ClinicalTrials.gov NCT06781099; ID-RCB 2024-A02473-44.

**STRENGTHS AND LIMITATIONS OF THIS STUDY**

- The primary outcome is quantified directly in the protein-free dialysis effluent by inductively coupled plasma mass spectrometry, with prespecified calibration, recovery and blank-subtraction criteria, thereby avoiding the analytical limitations of serum labile-iron assays.
- Adsorption of iron and of the MEX-CD1 polymer to the membrane and circuit is addressed by a prespecified polymer-recovery correction factor and by separate assay of a standardised circuit rinse.
- The deliberately conservative dialysis configuration (paediatric-sized dialyser, standard porosity, low blood and dialysate flow rates) is likely to underestimate the iron extraction achievable with the MEX-CD1 system.
- The single-centre, single-arm, open-label design with 10 evaluable participants, a 1-week intervention and 90-day follow-up permits no inferential comparison and captures neither end-organ iron burden nor transfusion independence.

## Background and rationale

 Post-transfusion iron overload is a major complication of repeated transfusions required for various haematological diseases: thalassaemia, sickle cell disease, myelodysplastic syndromes (MDS), etc. As iron stores increase, plasma transferrin becomes quickly saturated, leading to the presence of free iron in the bloodstream.^1^ Free iron can cross cell membranes in certain organs and act as a catalyst to form reactive oxygen species, causing cellular damage.^2 3^ Even outside of haematologic diseases, iron metabolism is disrupted during inflammation and emerges as a therapeutic target, especially in critically ill patients.^4^ Patients with MDS frequently experience anaemia, which requires regular transfusions. Transfusion-dependent MDS patients also suffer from ineffective erythropoiesis, which can disturb iron balance and lead to iron overload. Tissue iron overload can be detected within months of starting transfusions and is responsible for significantly increased mortality due to organ damage,^5^ particularly of the heart and liver. The bone marrow is also affected, and iron overload worsens dysmyelopoiesis and the risk of transformation to myelodysplasia,^6^ impacting stem cell transplant prognosis.

Iron homeostasis in myelodysplastic syndromes and in other marrow-failure states is not governed by transfusional input alone. Ineffective erythropoiesis drives the erythroid regulators erythroferrone and GDF-15, which suppress hepatic hepcidin transcription; the resulting hepcidin–ferroportin imbalance maintains intestinal iron absorption and macrophage iron release even when body iron stores are already expanded. Reported hepcidin concentrations in MDS are heterogeneous and vary with WHO subtype, transfusion history and molecular background, notably SF3B1 mutational status, which limits the value of hepcidin as a stand-alone biomarker but underlines that the transfused patient accumulates iron by both an exogenous and an endogenous route.^7^ This dual mechanism is one reason why tissue iron overload in MDS develops within months of the onset of transfusion support and why interrupting transfusion alone does not reverse it.

The clinical consequences of this accumulation are mediated not by total body iron but by the small, redox-active fraction of circulating iron, and the terminology used to describe that fraction requires precision. Once transferrin saturation exceeds approximately 70–75%, iron appears in plasma in forms that are not bound to transferrin (non-transferrin-bound iron, NTBI): a heterogeneous set of low-molecular-weight complexes with citrate, acetate and, in part, albumin. Labile plasma iron (LPI) designates the redox-active and chelatable subset of NTBI, the component that participates in Fenton chemistry, generates reactive oxygen species and enters cardiomyocytes and hepatocytes through non-transferrin routes such as ZIP14 and L-type calcium channels.^8^ NTBI and LPI are therefore not synonymous: LPI is operationally defined by a chelator-based fluorimetric assay, whereas NTBI is defined by what it is not, and different analytical methods yield systematically different values for both. In this protocol, 'NTBI’ denotes the non-transferrin-bound fraction as a whole; 'LPI’ is used only for the chelatable, redox-active subset as measured by a validated LPI assay; the unqualified terms 'free iron’ and 'labile iron’ are avoided. The species that cross a dialysis membrane are, by definition, the low-molecular-weight, non-transferrin-bound species, and we therefore designate the primary outcome as dialysable NTBI without equating it with LPI (see 'Rationale for outcome selection and analytical considerations’).

Haematopoietic stem cell transplantation is a high-risk situation for iron overload, which can develop extremely rapidly due to conditioning-induced erythropoiesis blockage. Iron overload has a negative prognostic impact on post-transplant survival.^9^ In patients undergoing long-term transfusions for conditions such as major thalassaemia, iron overload can reach approximately 2–12 g per year.^10^ Complications related to iron overload, such as cardiac or hepatic failure, endocrinopathies and increased infection risk, contribute to reduced survival of patients with MDS.

The aim of chelation therapy is therefore to prevent cardiac, hepatic and endocrine complications. There is currently no consensus on which chelator to recommend. The patient’s age, presence or absence of comorbidities, side effects of available treatments and patient preferences should be considered when initiating chelation therapy. In the USA and Europe, deferasirox and deferiprone are currently the most widely used chelators, both available orally.^11^ Deferasirox at 40 mg/kg enables net iron excretion of 0.445 mg/kg per day. The treatment goal is to achieve a ferritin level <1000 µg/L, liver iron concentration <7 mg/g and a cardiac MRI T2* >20 ms.^12^ Treatment intensification may be considered, particularly if there is cardiac failure related to iron overload, with deferiprone alone or in combination with deferasirox.^13 14^ There are numerous protocols for combining chelators in combined or sequential treatments.^13^ However, chelators may cause potentially debilitating side effects (nausea, vomiting, abdominal pain) that can compromise treatment adherence and render therapy ineffective.^15^ Thus, data from several retrospective studies suggest that iron overload can have clinical consequences in patients with MDS, including organ dysfunction and reduced overall survival.^16–18^ Thus, iron overload remains a significant issue in managing polytransfused patients,^15^ highlighting the need for alternative approaches to reduce this iron overload.

MEX-CD1 (medical device—Class III) is a highly chelating polymeric compound derived from chitosan—a natural polysaccharide—modified with DOTA-GA, a potent chelating agent already used in clinical imaging. DOTA-GA has a high affinity for metal cations, including iron. By keeping the polymer in the dialysate fluid compartment, separated from the blood by the semi-permeable membrane of the dialyzer, the system establishes a concentration gradient (figure 1). Free iron species in the plasma, which are small enough to pass through the membrane pores, diffuse into the dialysate, where they are trapped by the MEX-CD1 polymer.^19^ The polymer-iron complex is too large to back-diffuse into the blood, ensuring permanent removal. This approach is currently evaluated in several conditions where metallic cations accumulation exists.^20^

**Figure 1:**
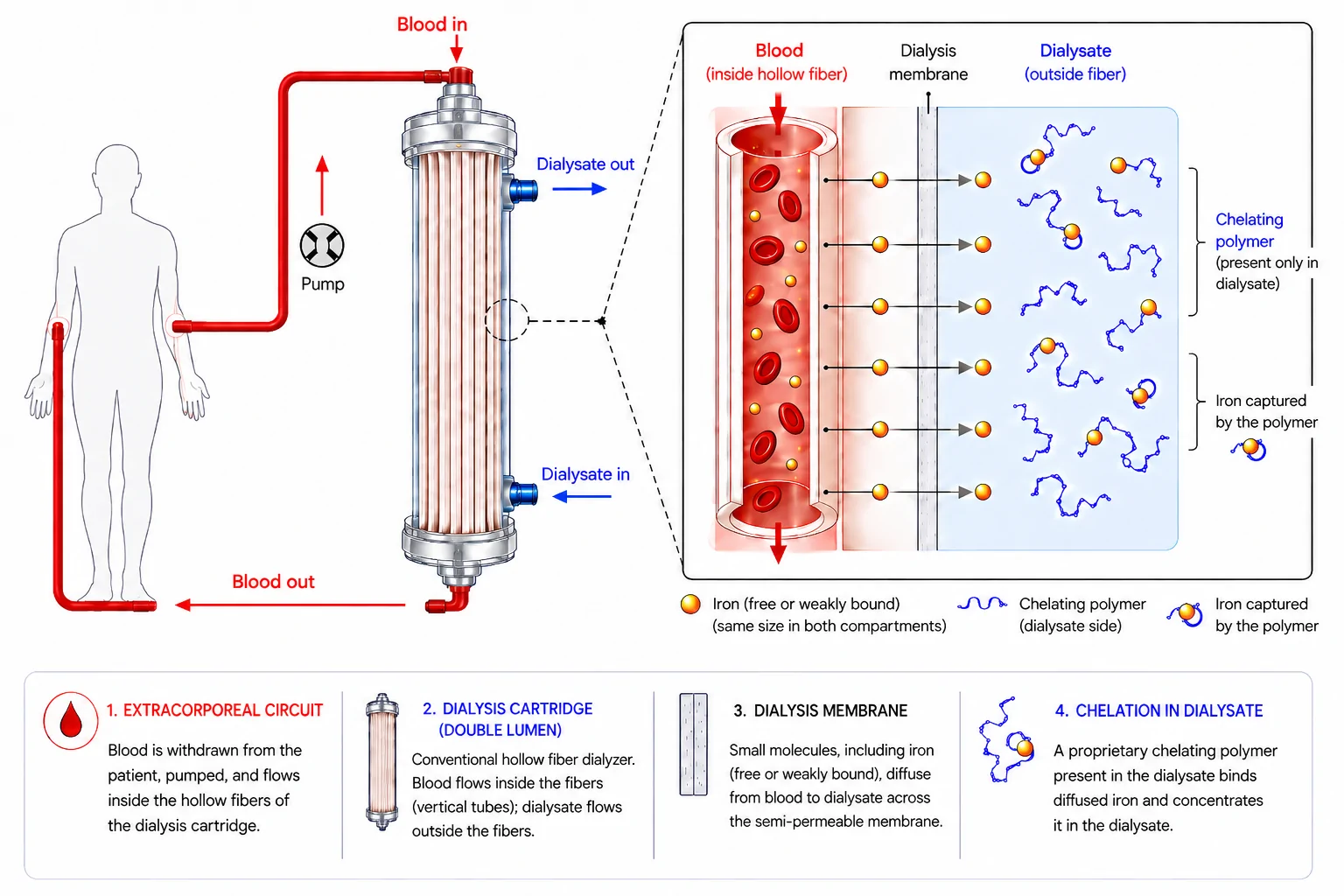
Schaematic representation of hemodialysis using a double-grafted chelating polymer designed for iron removal. Chelating polymers are unable to penetrate the dialysis membrane, unlike metallic ions that are weakly bonded to smaller molecules. These ions are then ensnared by the chelating polymers and become concentrated within the dialysate.

### Aim of the study

Unlike systemic chelation, in which the drug circulates throughout the body, the MEX-CD1 strategy confines the chelating agent to the dialysate compartment. The polymer remains separated from the patient’s blood by the semipermeable dialysis membrane. Non-transferrin-bound iron species diffuse from the blood across the membrane into the dialysate, where they are rapidly and irreversibly bound by the hyper-chelating polymer. The rationale for this configuration is that it minimises systemic exposure to the chelating agent, and hence the adverse-effect profile associated with systemic distribution, while retaining the capacity to remove circulating non-transferrin-bound iron. Whether that capacity is quantitatively meaningful in patients with iron overload is the question this study is designed to address.

We hypothesise that extracorporeal removal of non-transferrin-bound iron is feasible in patients with transfusional iron overload, with a good tolerance profile. This study aims to evaluate the performance and the safety of the MEX-CD1 chelating polymer used in combination with short CVVHD sessions for the removal of dialysable NTBI in patients with transfusion-dependent anaemia and documented iron overload.

We will conduct a single-centre, first-in-disease Phase I–II investigation of the safety, tolerability and mechanistic proof-of-concept of this extracorporeal approach. The MEX-CD1 polymer has been administered to humans in other indications, including Wilson’s disease (MEXWILS, NCT05917327); the present study is therefore not a first-in-human study of the device, but the first use of the approach in transfusional iron overload.

### Primary objective

The primary objective of this study is to establish the mechanistic feasibility of extracorporeal iron depletion using the MEX-CD1 device. Specifically, to determine whether a measurable and quantifiable mass of dialysable NTBI is recovered in the dialysis effluent across three sessions in this first-in-disease context. Demonstrating that the device physically extracts iron from the circulation is a necessary proof-of-concept step, without which the design of any subsequent trial targeting clinical endpoints would be premature.

### Secondary objectives

The secondary objectives are to evaluate the safety and clinical feasibility of the procedure, with attention to adverse events such as intradialytic hypotension and disturbances of electrolytes and trace elements; to describe the kinetics of iron removal within and across sessions; to describe changes in serum ferritin and transferrin saturation; and to describe quality of life and subsequent transfusion requirements. These secondary objectives are exploratory and descriptive; the study is not designed to establish a clinical effect.

## Methods and analysis

### Study design

The MEXIRON study is a single-arm, prospective, monocentric, open-label, non-comparative, first-in-disease Phase I–II study. The Phase I component addresses safety and tolerability in a fragile population exposed to the approach for the first time; the Phase II component addresses the mechanistic signal of iron removal. The study is sponsored by the Hospices Civils de Lyon. All eligible patients undergo three CVVHD sessions over 1 week, without a comparative arm. The modality is CVVHD—continuous veno-venous haemodialysis, that is, purely diffusive clearance delivered on a continuous renal replacement therapy platform—operated on an intermittent schedule of three discrete sessions. The design and study timeline are summarised in figure 2. The complete study protocol is provided in the online supplemental material.

**Figure 2:**
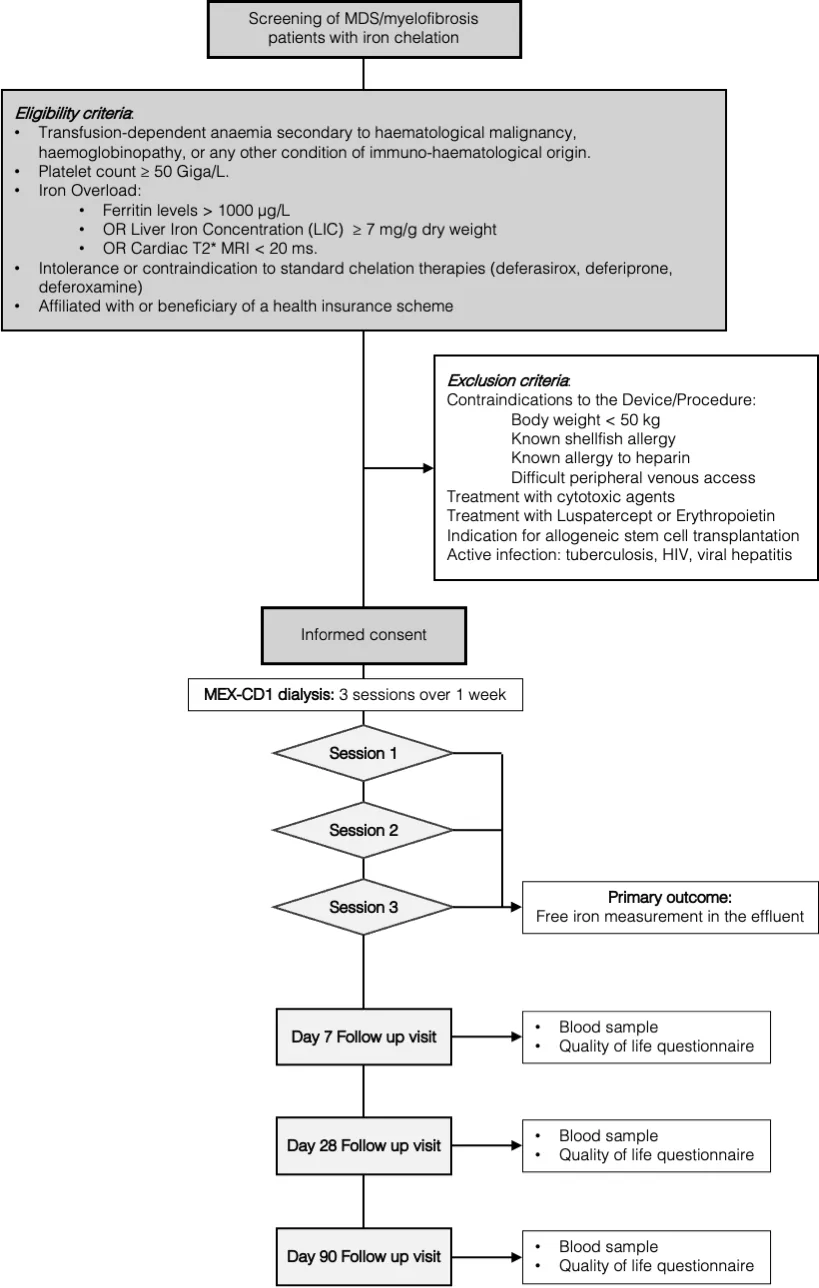
Flow chart of the MEXIRON study.

### Eligibility criteria

Eligible patients are adults who are dependent on red blood cell transfusion in the context of a haematological malignancy, a haemoglobinopathy, or any other condition of immuno-haematological origin, who have documented transfusional iron overload and who have no viable alternative for its management. Participation will be proposed by the attending haematologist during a follow-up consultation. The eligibility criteria reported below correspond to the amended version of the protocol; the amendment is described, with its rationale, in 'Rationale for the eligibility criteria’.

### Inclusion criteria

1. Adults with transfusion-dependent anaemia secondary to a haematological malignancy (including myelodysplastic syndrome and myelofibrosis), a haemoglobinopathy, or any other condition of immuno-haematological origin.
2. Iron overload of transfusional origin, defined as: serum ferritin>1000 µg/L (on two successive measurements at least 2 weeks apart), OR liver iron concentration (LIC)≥7 mg/g dry weight, OR cardiac T2* MRI<20 ms.
3. Chelation failure and/or intolerance. Failure is defined by a stable (≥80% of baseline) or increasing ferritin level after at least 6 months of continuous treatment at the maximum tolerated dose.
4. Platelet count≥50 × 10⁹/L.
5. Established dependence on red blood cells (RBC) transfusions with haemoglobin levels<80 g/L requiring support.
6. Able to understand the study information and to give written informed consent personally.
7. Affiliated with or beneficiary of a health insurance plan.

### Exclusion criteria

1. Contraindications to the device and haemodialysis procedure:

- Body weight<50 kg.
- Known shellfish allergy (as the chitosan in MEX-CD1 is of animal origin).
- Known allergy or contraindication to heparin

Patients with difficult peripheral venous access or those in whom venous capital must be strictly preserved (eg, imminent need for chronic renal dialysis).

Treatment with azacitidine (as monotherapy or in combination) or with any other chemotherapy, or agent considered as such, administered on a repeated or monthly schedule for acute leukaemia, myelodysplastic syndrome or myelofibrosis. Patients treated with a JAK1/2 inhibitor may be included provided that the treatment was introduced at least 3 months before inclusion and that the dose has not been modified during the 4 weeks preceding inclusion.

Treatment with luspatercept or with an erythropoiesis-stimulating agent (erythropoietin) received in the month preceding inclusion.

Allogeneic haematopoietic stem cell transplantation planned within 1 month of inclusion.

Known active infection such as tuberculosis, HIV/AIDS or positive screening for hepatitis B (HBsAg) or hepatitis C (RNA).

Participation in another interventional study that could interfere with results.

Pregnancy or breastfeeding.

Legal incapacity (guardianship/tutorship) or inability to express consent.

### Rationale for the eligibility criteria

#### Underlying diagnosis

Eligibility was initially restricted to myelodysplastic syndromes and myelofibrosis. In the light of the first months of screening, the study scientific committee reconsidered the target population and amended the criteria as reported above. The reasoning is as follows. The mechanism under investigation is not disease-specific: the device removes low-molecular-weight NTBI from plasma by diffusion into a chelating dialysate. The quantity available for removal is determined by transferrin saturation and the size of the circulating NTBI pool, not by the marrow disorder that generated the transfusion requirement. The primary outcome is a physicochemical quantity measured in the effluent, and there is no biological rationale for expecting it to differ between a myelodysplastic syndrome and another cause of iron overload. Restricting the study to a single diagnosis would therefore not have made the primary outcome more homogeneous. With ten evaluable patients, no inferential comparison between diagnostic subgroups is planned, and none would be interpretable; individual patient-level data, including the underlying disease, are presented for each participant alongside the extraction data, so that any disease-related pattern is visible.

#### Definition of iron overload

Serum ferritin, liver iron concentration and cardiac T2* are not interchangeable and identify partly different populations. They are nevertheless retained as alternative entry routes because each independently establishes a transfusional iron burden that justifies chelation and because requiring MRI in all candidates would restrict access without informing the primary outcome. The criterion actually met is recorded and reported for each participant.

#### Concomitant treatments

Patients receiving azacitidine or another chemotherapy on a repeated or monthly schedule are not eligible. These agents alter erythropoiesis, transfusion requirement and iron kinetics over precisely the time frame of the intervention and its 90-day follow-up, and with ten evaluable patients the resulting variability could not be distinguished from the effect of the device. The exclusion has been narrowed to repeated-schedule cytotoxic therapy: patients treated with a JAK1/2 inhibitor are eligible provided the treatment has been stable for at least 3 months with no dose change in the preceding 4 weeks, so that the patient is in a steady haematological state. Luspatercept and erythropoiesis-stimulating agents are excluded, for the same reason: both act directly on erythropoiesis and on the transfusion requirement and would therefore alter the very parameters against which iron extraction is to be interpreted. Ongoing treatments and any change during follow-up are recorded and reported.

#### Allogeneic transplantation

In the initial protocol, an indication for allogeneic haematopoietic stem cell transplantation was an exclusion criterion. Patients awaiting transplantation are among those in whom iron overload matters most, and the criterion has been narrowed accordingly: only patients for whom transplantation is planned within 1 month of inclusion are excluded, as conditioning blocks erythropoiesis and abruptly changes iron handling, and the transplant admission would overlap the follow-up window.

### Interventions

Participants will undergo a single treatment cycle consisting of three extracorporeal iron depletion sessions:

- The three sessions (S1, S2 and S3) are performed within a single week.
- Vascular access will be established using two peripheral catheters.
- Systemic anticoagulation with unfractionated heparin will be used to maintain circuit patency: 10 IU/kg/h, reduced to 5 IU/kg/h for patients with a platelet count between 50 and 100×10⁹/L.
- Sessions will be performed in CVVHD mode (continuous veno-venous haemodialysis, purely diffusive, without convective replacement fluid) on a Multifiltrate platform (Fresenius Medical Care), with a paediatric-sized dialyser of standard porosity, a blood flow of 70 mL/min and a dialysate flow of 600 mL/h.
- For each session, 2 L of dialysate (multiBic K+0 mmol/L, Fresenius Medical Care) will be used, after addition on MEX-CD1 compound for a final concentration of 0.5 g/L.
- Each session will be programmed for 200 min (3 hour 20 min), corresponding to the single-pass delivery of the full 2 L of chelating dialysate at 600 mL/h.

### Additional treatments

Trace element supplementation will be standardised for all participants to mitigate the risk of deficiency. At the end of each session, patients will receive a parenteral formulation containing 7 mg of zinc gluconate, 44 µg of sodium selenite, 1.5 mg of copper gluconate, 13 µg of potassium iodide and 40 µg of manganese gluconate. If required, transfusion will be performed before the start of a session; platelet transfusion is mandatory if the platelet count is <50 × 10⁹/L on the day of the session.

### Rationale for the dialysis prescription

- Modality. The sessions are delivered on a continuous renal replacement therapy platform operated in CVVHD mode. This platform was chosen in preference to a conventional intermittent haemodialysis monitor because it permits the low, precisely controlled dialysate flow rate (600 mL/hour) required to work with a fixed 2 L volume of MEX-CD1-containing dialysate. The schedule is nevertheless intermittent: a continuous-mode diffusive circuit delivered in three discrete sessions.
- Duration. The 200 min duration is the time required to deliver the entire prepared volume of chelating dialysate exactly once in single-pass mode, without recirculation.
- Session spacing. We chose to perform the three sessions within a single week. Biologically, the plasma NTBI pool is replenished rapidly from tissue and macrophage stores; closely spaced sessions allow the kinetics of that replenishment, and any cumulative effect across sessions, to be observed within a single physiological state, whereas weekly sessions would be separated by intervening transfusions that would confound the within-patient comparison. From a monitoring standpoint, a 1 week cycle keeps the whole intervention within a single closely supervised observation window, which the sponsor and the ethics committee considered appropriate for a first exposure of this population to the device. No regulatory requirement imposed this schedule.

### Outcomes

#### Primary outcome

The primary outcome is the mass of iron recovered in the effluent bag at the end of each of the three sessions (S1, S2, S3), expressed in milligrams and measured by inductively coupled plasma mass spectrometry. Because the dialysis membrane retains transferrin and other iron-binding proteins, this quantity is designated dialysable NTBI; it is not reported as labile plasma iron (see below).

### Rationale for outcome selection and analytical considerations

In keeping with the Phase I–II design of this study, the primary outcome addresses the Phase II mechanistic question: whether a measurable and quantifiable mass of NTBI is recoverable in the dialysis effluent in this patient population. This proof-of-concept demonstration is a necessary prerequisite for the design of any subsequent trial targeting clinical endpoints. Quantifying labile iron in serum presents methodological difficulties, as there is no universally accepted gold standard and the available assays are chelator-based and operationally defined. We therefore chose to measure total iron in the dialysis effluent. Because of the approximately 30 kDa cut-off of the dialyser membrane, transferrin, ferritin and other iron-binding proteins do not cross into the effluent, so that the iron recovered there necessarily belongs to the low-molecular-weight, non-transferrin-bound fraction, that is, to NTBI. The potential adsorption of the MEX-CD1 polymer and of iron to the dialyser membrane and circuit, which would lead to underestimation, is addressed by the prespecified correction and rinse procedures described in the following section.

### Analytical methods for iron quantification

Iron removal will be quantified by inductively coupled plasma mass spectrometry (ICP-MS). Given the very high complexation constant of DOTA for Fe(III) (log K=29.4),^21^ the fraction of Fe(III) remaining unbound to the chelating polymer in the dialysate is expected to be negligible. Therefore, the iron concentration measured in the dialysate should predominantly reflect iron chelated by the polymer. However, as previously observed in preclinical studies (pending publication) partial adsorption of the polymer onto the dialysis membrane occurs. Consequently, direct measurement of iron in the dialysate alone may underestimate the total amount of iron removed, since a fraction of the iron-loaded polymer may remain associated with the membrane. To account for this phenomenon, the polymer concentration in the dialysate will be directly measured and quantified using label-free high-performance liquid chromatography coupled with electrospray ionisation tandem mass spectrometry (HPLC-ESI-MS/MS). The amount of polymer retained on the dialysis membrane will then be estimated from the difference between the initial amount of polymer introduced and the amount recovered in the dialysate.

For the calculation of total iron removal, we will conservatively assume that the iron-to-polymer ratio is identical for polymer remaining in solution and polymer adsorbed onto the dialysis membrane. This assumption is expected, if anything, to underestimate rather than overestimate total iron removal, as membrane-associated polymer may retain or exhibit increased accessibility to iron and could therefore display a higher degree of iron complexation. Accordingly, the estimated amount of iron removed using this approach should be regarded as a conservative estimate.

### Expected magnitude of iron extraction

An explicit a priori expectation is stated so that the observed extraction can be interpreted against a prespecified benchmark. In the in vivo ovine feasibility experiments that preceded this trial, a 4 hour session using Chitosan@DOTAGA at 1 g/L in 1 L of dialysate extracted approximately 0.5 mg of iron in animals with normal iron status.^22^

A worked example follows. For a 70 kg patient with serum ferritin 2500 µg/L, transferrin saturation 90% and an estimated NTBI of 5 µmol/L (0.28 mg/L), and assuming complete equilibration of the effluent with plasma NTBI, the expected extraction is 2 L×0.28 mg/L ≈ 0.56 mg per session, that is approximately 1.7 mg over the three-session cycle. Under the more conservative assumption of 50% equilibration, which is plausible with the standard-porosity paediatric dialyser used here, the expected values are approximately 0.28 mg per session and 0.8 mg per cycle.

The purpose of this trial is not to demonstrate a clinically meaningful reduction in iron stores, but to establish that the device removes a quantifiable mass of the NTBI species that mediate iron toxicity, and to characterise the safety of the procedure, in a deliberately conservative configuration. Should this proof of mechanism be established, extraction is expected to scale with membrane surface area, membrane porosity and dialysate flow, and these are the parameters that a subsequent dose-finding study would address. A recovered mass below 0.1 mg per session, or below the limit of quantification after blank subtraction, would be interpreted as a failure of the mechanistic proof-of-concept in this configuration.

### Secondary outcomes

1. **Extraction kinetics:** concentration of iron in the effluent measured hourly on the effluent line (H1, H2 and end of session) during each session.
2. **Biological effect (exploratory):**

- Changes in serum iron, non-transferrin-bound iron, ferritin and the transferrin saturation coefficient (TSC) from the start (H0) to the end of each session.
- Cumulative changes in these parameters from the start of the first session to the end of the third session, and at follow-up visits (V1, V2 and V3).

**Safety and tolerance:**

- Incidence of Adverse Events (AEs) and Serious Adverse Events (SAEs).
- Specific monitoring for Adverse Events of Special Interest (AESI), including intradialytic hypotension, anaphylactoid reactions, vascular access complications and post-session electrolyte disturbances (hypocalcaemia, hypomagnesaemia, hypokalaemia and hyponatraemia).
- Patient-reported tolerance using a Visual Analogue Scale (VAS) for fatigue/malaise and pain.

Clinical Impact: Assessment of transfusion requirements (number of Red Blood Cell units and dates of transfusion) following the intervention.

Feasibility:

- Proportion of patients completing the first session with full treatment.
- Proportion of patients completing the full cycle of three sessions.
- Ratio of effective treatment time to programmed time (200 min).
- Total volume of blood treated.

Quality of Life: Evolution of quality-of-life measured by the MOS SF-12 self-administered questionnaire at inclusion and last visit of follow-up (V3).

Ancillary Study:

Trace metals: Quantification of copper, zinc, selenium and lead extraction to assess the specificity of the chelation.

### Statistical power and sample size

The sample size reflects the pilot nature of the study. The purpose is to obtain preliminary data on the feasibility and the variance of iron removal in order to inform the design of a future trial. No formal power calculation based on a hypothesis test was performed, and none would be meaningful for a mechanistic feasibility endpoint. The protocol specifies a target of 10 evaluable patients, that is, patients who complete at least one full dialysis session; we plan the recruitment of a maximum of 13 patients to reach that target. This sample is sufficient to describe safety and to estimate the central tendency and the dispersion of iron extraction and is not sufficient for any inferential claim. Statistical analysis will be primarily descriptive.

### Analysis populations

Two analysis populations are prespecified, and they account for the difference between the 13 patients to be recruited and the target of 10 evaluable patients. These two definitions are based solely on treatment exposure (cannulation attempted; full session delivered) and apply identically to all patients regardless of underlying diagnosis, including those recruited under the amended eligibility criteria.

The safety population comprises all included patients in whom the procedure was initiated, that is, in whom cannulation was attempted, irrespective of whether a dialysis session was subsequently delivered. It is the population for all adverse events, adverse events of special interest, vital signs and biological safety data, which are analysed as treated.

The evaluable population comprises the patients who completed at least one full dialysis session, that is, a session delivered to its programmed 200 min term. It is the population for the analysis of iron extraction, each patient contributing the sessions actually completed (i.e. sessions delivered to their full programmed term; a session interrupted before term is excluded from this calculation and is reported separately, see Handling of incomplete sessions below). The target of 10 evaluable patients refers to this population. A total of 13 patients will be recruited to reach it, because a proportion of included patients may not complete a first full session (e.g. because of inadequate peripheral venous access, adverse event of the dialysis procedure).

#### Handling of incomplete sessions

A session interrupted before its programmed term does not count towards evaluability, but the data collected during it are neither discarded nor imputed: the effluent bag is assayed, and the iron recovered is reported together with the effective treatment time and the reason for interruption. Patients in whom no full session was completed remain in the safety population and are described individually. No imputation of missing biological data is planned; the number of missing values is reported for every variable. The number of patients in each population, and the reason for non-evaluability in each case, will be reported in the participant flow diagram.

### Biological sample collection

Plasma and PBMCs (peripheral blood mononuclear cells) will be collected for future transcriptomic analysis of circulating mature elements (bio-banking). Any future analysis will be described in a separate protocol and will be subject to the appropriate regulatory approvals.

### Data collection

All information required by the protocol will be recorded in an electronic case report form (eCRF). This eCRF, specific to the study, will be developed by a data manager from the Hospices Civils de Lyon on the Ennov Clinical software (V.8.2.50). Patients follow-up and data collected are detailed in table 1.

**Table 1:** Study data collection

Procedures and assessments	Inclusion	Treatment cycle						Follow-up		
		Session 1		Session 2		Session 3		Visit 1	Visit 2	Visit 3
		*Start*	*End*	*Start*	*End*	*Start*	*End*	S3+7 days	S3+28 days	S3+90 days
Inclusion/non-inclusion criteria	X									
Characteristics at baseline*	X	X		X		X		X	X	X
Information and consent	X									
Iron metabolism biomarkers†		X	X	X	X	X	X	X	X	X
Inflammatory biomarkers‡		X	X	X	X	X	X	X	X	X
Vital parameters**		X		X		X				
Pain & discomfort assessment			X		X		X			
Quality of life evaluation§	X								X	X
Ancillary study and bio-banking										
Trace metals††		X	X	X	X	X	X	X	X	
Bio-banking¶	X			X				X		X
Adverse events monitoring	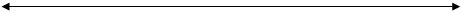									

* Characteristics of the patient include age, gender, height, weight, BMI, performance status.

† Iron metabolism biomarkers include serum iron, transferrin, transferrin saturation, soluble transferrin receptor, ferritin.

‡ Inflammatory biomarkers include C reactive protein and neutrophils.

§ Vital parameters include weight, temperature, blood pressure, heart rate, SpO_2_

¶ Collection of plasma and PBMCs for future transcriptomic analysis of circulating mature elements.

** Vital parameters include weight, temperature, blood pressure, heart rate, SpO_2_.

†† Samples will be taken on the venous line, the effluent line and bag and dialysate bag.

The data set will be computerised in a coded way, in accordance with the law for data protection and freedom of information (Article L.1121-3 of the French Public Health Code). The study families will be identified by a unique study inclusion number and by the first initial of their surname and of their given name.

Data will be entered, as soon as they are collected, by the authorised persons (investigator and personnel recorded on the delegation log) according to the law for data protection and freedom of information.

Access to the data will be restricted to the authorised persons only. Authentication will be made using passwords, which will be regularly changed. The investigators and clinical research assistants of an investigating centre will only have access to the data for their patients and will enter the data directly into the eCRF using a secured website. The investigator will be responsible for the reliability of the data entered. Throughout the length of the study, the data will be stored in an ISO 27001-certified data centre and backed up daily. All adverse events will be recorded, coded (MedDRA) and reported in accordance with regulatory requirements. Data will be securely stored, pseudonymised and monitored for completeness and consistency. Analysis datasets will be frozen only after quality control and cleaning.

### Statistical analysis plan

The patients’ characteristics and the baseline biology will be described according to the standard statistics defined below. Quantitative data will be described for the entire population using the following descriptive statistics: sample size, number of missing values, mean, SD, median, first and third quartiles, as well as minimum and maximum values. These statistics will be considered standard for the analysis of quantitative variables. Quantitative variables may be categorised according to a predefined cut-off. The CIs provided will be two-sided 95% CIs. Qualitative variables will be summarised for the entire population using the following descriptive statistics: sample size and percentage of missing values, frequencies and percentages for each level of the variable (missing values will not be included in the denominator when calculating frequencies). These statistics will be considered standard for the analysis of qualitative variables. When necessary, CIs for proportions will be calculated based on an exact binomial distribution. A graphical longitudinal description of the biomarkers will be provided.

Given the pilot nature of this study and the limited number of evaluable participants, the analysis will be primarily descriptive. NTBI, ferritin and TSC will be summarised at each time point using means and SD, or medians and IQRs as appropriate, both overall and by session (S1, S2, S3). Individual trajectories and mean profiles will be displayed graphically. Within-session changes will be described for each session, with their 95% CIs.

As a secondary and exploratory step, the primary endpoint will be modelled using a linear mixed-effects model to account for the repeated-measures structure of the data. Fixed effects will include session (S1, S2, S3) and time within session; an interaction between time and session may be explored if the data support it. The random-effects structure will have a random intercept per patient, which captures between-patient heterogeneity in baseline iron status and the within-patient correlation of repeated measurements. A random intercept for session nested within patient may be added when several measurements are available within a session, provided the model converges to a non-singular solution.

If a model fails to converge or yields a singular fit, the following pre-specified simplifications will be applied in order:

- Retaining a random intercept for patient only.
- Adopting a summary-measures approach in which one value per patient and per session (within-session change or individual slope) is analysed using paired non-parametric tests.

The magnitude of the intervention effect will be tested using a Wald test; the 95% CI of the mean difference will be provided. CIs will be reported throughout and interpreted as estimates of precision. No confirmatory claim will be based on p-values.

Changes in biomarker values (NTBI, ferritin, TSC) between the start of the first session (S1H0) and the end of the third session (S3) will be analysed as absolute differences as well as percentage changes. Changes in NTBI and TSC measured between the start of the first session (S1H0) and V1 (S3+D7) / V2 (S3+D28) / V3 (S3+D90) will be analysed as absolute differences as well as percentage changes. Data transformations may be performed if necessary to satisfy the normality assumptions of the model.

### Patients and public involvement

Patients and/or the public were not involved in the design, conduct, or reporting, or dissemination plans of this research.

## Ethics and dissemination

### Ethical approval and regulatory compliance

The study is conducted in strict adherence to the ethical principles of the Declaration of Helsinki and the Good Clinical Practice (GCP) guidelines.

The study protocol and the informed consent form were reviewed and approved by the Ethics Committee Ouest III from Poitiers (No. 25.00336.000384). The study has been authorised from the National Agency for Safety of Drugs and Medical Devices (April 10, 2025, No. IDRCB: 2024-A02473-44) prior to the initiation of patient recruitment in compliance with the applicable research and human subjects regulations. The study is registered on ClinicalTrials.gov under the identifier NCT06781099.

### Trial status

Regulatory authorisation was granted on 10 April 2025. The first participant was included in January 2025. At the date of submission of this revised manuscript, 1 participant had been included with one treatment cycle completed. Recruitment is expected to be completed by January 2028, with last-patient last-visit 90 days thereafter. The version of the protocol described in this manuscript is version seven dated July 31^st^, 2026.

### Stopping rules—individual level

A session is interrupted immediately in the event of: a fall in systolic blood pressure below 90 mmHg, or by more than 30% from baseline, not responding to fluid challenge and to a reduction in blood flow; any suspected anaphylactoid reaction; symptomatic hypocalcaemia, hypokalaemia or hyponatraemia; circuit clotting that cannot be resolved by a circuit change; or a request from the patient. A participant who has had a session interrupted because of an event attributed to the device or to the procedure does not undergo further sessions.

### Stopping rules—study level

Recruitment is suspended, and the sponsor, the independent safety monitoring committee and the competent authority are notified, in the event of: any death or life-threatening event considered related or possibly related to the device or the procedure; any event meeting the criteria of a suspected unexpected serious adverse reaction; two or more anaphylactoid reactions attributed to MEX-CD1; or an adverse event of special interest attributed to the procedure in three or more of the first six participants. The study is not resumed until the safety monitoring committee has reviewed the events and the sponsor has confirmed the acceptability of the benefit–risk balance. Because this is a single-arm mechanistic study with no efficacy hypothesis, no interim analysis of the primary outcome and no efficacy or futility stopping rule is planned.

### Protocol amendment

Any modifications to the protocol which may impact on the conduct of the study, potential benefit of the patient or may affect patient safety, including changes of study objectives, study design, patient population, sample sizes, study procedures or significant administrative aspects will require a formal amendment to the protocol. Such amendment will be approved by the ethics committee prior to implementation and notified to the health authorities in accordance with French regulations.

### Informed consent procedure

Written informed consent of the patient is a preliminary requirement for participation. The investigator will provide detailed oral and written information to the patient regarding the study’s objectives, procedures, potential risks (related to haemodialysis and the device) and benefits. Patients are informed of their right to withdraw consent at any time without prejudice to their ongoing care. Specific consent is also obtained for the collection of biological samples for the ancillary bio-banking and for the processing of personal data.

### Confidentiality

Personal information will be anonymised and securely stored. Access to identifiable data will be restricted to authorised study staff, the sponsor and regulatory bodies for purposes such as monitoring, auditing, or inspection, in compliance with French data protection regulations and Good Clinical Practice.

All study-related information is stored securely at the study site. All reports, data collection, process and administrative forms are identified by a coded ID number only to maintain participant confidentiality. All records that contain names or other personal identifiers, such as informed consent forms, are stored separately from study records identified by code number. All local databases are secured with password-protected access systems. The study has been previously registered in compliance with requirements from the French data protection authority (Commission Nationale Informatique et Libertés).

### Dissemination plan

The findings from the MEXIRON study will be shared with the scientific community. Any significant changes to the protocol will be updated on ClinicalTrials.gov. The study’s outcomes will be published in peer-reviewed scientific journals and presented at both national and international conferences. Patients or their representatives can request a summary of the study’s findings. All publications and presentations will recognise the sponsor and funding sources and will include authorship in line with ICMJE criteria.

### Data sharing statement

Deidentified individual participant data underlying the results reported in the forthcoming main manuscript, together with the data dictionary and the statistical code, will be made available beginning 12 months after publication and for 5 years thereafter, to investigators whose proposed use of the data has been approved by an independent review committee for the purpose of achieving the aims of an approved proposal. Requests should be addressed to the corresponding author and will require a signed data access agreement.

## Supplementary material

10.1136/bmjopen-2026-121216 online supplemental file 1
