# Supplementary material for: Protocol for the MEXIRON study: a first-in-disease phase I–II investigation into the safety, tolerability and mechanistic proof-of-concept of extracorporeal iron depletion using the MEX-CD1 device in patients with transfusional iron overload
Source: BMJ Open. 2026 Oct 27;16(10):e121216. doi: 10.1136/bmjopen-2026-121216 (PMC13723837; doi:10.1136/bmjopen-2026-121216)
Supplement: Supplementary file 1 [file bmjopen-16-10-s001.pdf]

Etude pilote de faisabilité, tolérance et efficacité de l'épuration extracorporelle du fer chez des patients présentant une surcharge martiale nécessitant une stratégie de déplétion en fer associée à une intolérance, une contre-indication ou une inefficacité à un traitement chélateur oral ou sous cutané.

MEXIRON

Protocole d'investigation clinique (cas n°2)

Version 7 du 28/07/2026

Promoteur : Hospices Civils de Lyon
BP 2251
3 quai des Célestins
69229 LYON Cedex 02

Investigateur principal : **Dr Auguste DARGENT**
Service d'Anesthésie - Réanimation - Médecine Intensive
Hôpital Lyon Sud
Hospices Civils de Lyon
165 Chemin du grand revoyet
69495 OULLINS-PIERRE-BENITE Cedex
Tél. : 04 78 86 20 06
Email : auguste.dargent@chu-lyon.fr

Coordinateur Scientifique : **Pr Bernard ALLAOUCHICHE**
Service d'Anesthésie - Réanimation - Médecine Intensive
Hôpital Lyon Sud
Hospices Civils de Lyon
165 Chemin du grand revoyet
69495 OULLINS-PIERRE-BENITE Cedex
Tél. : 04 78 86 23 42
Email : bernard.allaouchiche@chu-lyon.fr

Code promoteur : 69HCL23_0102
N°ID-RCB / EUDAMED : 2024-A02473-44
Numéro d'enregistrement clinicaltrials.gov : NCT06781099
Avis favorable du CPP Ouest III le : 13/03/2025
Autorisation de l'ANSM le : 10/04/2025

LISTE DES ABREVIATIONS

**ALR**	Autorisation de Lieu de Recherche
**ANSM**	Agence Nationale de Sécurité des Médicaments et des produits de santé
**ARC**	Attaché de Recherche Clinique
**BI**	Brochure Investigateur
**BMF**	Bone Marrow Failure
**BPC**	Bonnes Pratiques Cliniques
**CE**	Communauté Européenne
**CEC**	Circulation ExtraCorporelle
**CFR**	*Code of Federal Regulations*
**CGR**	Concentrés de Globules Rouges
**CNIL**	Commission Nationale Informatique et Liberté
**CPP**	Comité de Protection des Personnes
**eCRF**	*Case Report Form* (cahier d'observation) électronique
**CRP**	Protéine C-réactive
**CSI**	Comité de Surveillance Indépendant
**CSP**	Code de Santé Publique
**CST**	Coefficient de Saturation de la Transferrine
**CTCAE**	*Common Terminology Criteria for Adverse Events*
**DM**	Dispositif Médical
**DRS**	Direction de la Recherche en Santé
**EDTA**	Acide éthylènediaminetétraacétique
**EER**	Epuration extrarénale
**EI**	Evénement Indésirable
**EIG**	Evénement Indésirable Grave
**EIP**	Evènement d'Intérêt Particulier
**EVA**	Echelle Visuelle Analogique
**FDA**	*Food and Drug Administration*
**FN**	Fait Nouveau
**HCL**	Hospices Civils de Lyon
**IC**	Investigation Clinique
**ICH**	*International Conference on Harmonisation*
**ILM**	Institut Lumière Matière (Université Claude Bernard Lyon 1)
**IMC**	Indice de Masse Corporelle
**IRM**	Imagerie par Résonance Magnétique
**ITT**	*Intention To Treat*
**ISO**	*International Organization for Standardization*
**MDCG**	Groupe de Coordination en matière de Dispositifs Médicaux
**MOS SF-12**	*Medical Outcome Study Short Form 12*
**MR**	Méthodologie de Référence
**NFS**	Numération Formule Sanguine
**PBMC**	Celulles mononucléaires du sang périphérique

**POS**	Procédures Opératoires Standardisée
**PT**	*Preferred Term*
**PUI**	Pharmacie à Usage Intérieur
**POS**	Procédures Opératoires Standards
**RCP**	Résumé des Caractéristiques du Produit
**RDM**	Règlement européen du Dispositif Médical
**RGPD**	Règlement Général sur la Protection des Données
**SaO_2**	Saturation artérielle de l'hémoglobine en oxygène
**SMD**	Syndrome myélodysplasique
**SOC**	*System Organ Class*
**SUSAR**	*Suspected Unexpected Serious Adverse Reaction*
**TAD**	Tension Artérielle Diastolique
**TAS**	Tension Artérielle Systolique
**TMF**	*Trial Master File*
**UI**	Unité Internationale
**VRB**	Volontaires pour les Recherches Biomédicales

CONFIDENTIEL

RESUME

**Titre**	MEXIRON : Etude pilote de faisabilité, tolérance et efficacité de l'épuration extracorporelle du fer chez des patients présentant une surcharge martiale nécessitant une stratégie de déplétion en fer associée à une intolérance, une contre-indication ou une inefficacité à un traitement chélateur oral ou sous cutané.
**Titre compréhensible par personnes profanes**	MEXIRON : Etude pilote de performance et tolérance du dispositif MEX-CD1 pour l'épuration extracorporelle du fer chez des patients et présentant présentant une surcharge en fer, nécessitant une stratégie de déplétion en fer associée à une intolérance, une contre-indication ou une inefficacité à un traitement chélateur.
**Promoteur**	Hospices Civils de Lyon<br>BP 2251<br>3 quai des Célestins,<br>69229 LYON cedex 02<br><br>Représentant légal :<br>Alexandre PACHOT, Directeur de la Recherche en Santé
**Numéro promoteur**	69HCL23_0102
**Numero ID-RCB / Numéro d'identification unique du DM (EUDAMED)**	2024-A02473-44
**Investigateur principal**	**Dr Auguste DARGENT**<br>Service d'Anesthésie - Réanimation - Médecine Intensive<br>Hôpital Lyon Sud<br>Hospices Civils de Lyon<br>165 Chemin du grand revoyet<br>69495 OULLINS-PIERRE-BENITE Cedex<br>Tél. : 04 78 86 20 06<br>Email : auguste.dargent@chu-lyon.fr
**Version du protocole**	Version 7 du 28/07/2026
**Modalités de financement investigation clinique**	Cette étude a obtenu un financement dans le cadre de l'appel à projets « Innovation Clinique » porté par les Hospices Civils de Lyon et Lyonbiopôle. Un co-financement de la société MexBrain est également prévu.
**Scénario général de l'investigation clinique**	Etude pilote non comparative, ouverte, sur une cohorte de 10 patients traités séquentiellement lors de séances d'Epuration ExtraCorporelle (EEC) utilisant le dispositif médicale MEX-CD1.<br>Etude pilote monocentrique, multisites HCL, prospective, descriptive.
**Données d'identification et description du dm**	Nom : MEX-CD1<br>Fabricant : Mexbrain (13 avenue Albert Einstein - 69100 Villeurbanne)<br>Le MEX-CD1 est un composé polymérique hyper chélatant utilisé en combinaison avec une technique d'hémodialyse à faible volume pour extraire du sang le fer en excès.
**Informations sur le DM**	Le MEX-CD1 est un dispositif médical de Classe III, non marqué CE.
**Caractéristiques techniques et fonctionnelles du DM**	La stratégie thérapeutique proposée repose sur la combinaison de deux technologies déjà présentes en clinique et permettant de réduire les inconvénients intrinsèques à chacune : la dialyse et la chélation. Dans cette technologie, le composé actif (MEX-CD1) est maintenu séparé du sang par la membrane de dialyse. Le MEX-CD1 a été conçu à partir d'un polysaccharide naturel, le chitosane, en y associant un chélate déjà utilisé en clinique le DOTA-GA. Ce polymère fonctionnalisé présente l'avantage d'être biocompatible et fortement chélatant. Il n'existe actuellement pas de dispositif alternatif sur le marché pour l'épuration extracorporelle de métaux lourds.
**Risques et bénéfices du dm**	**Risques potentiels**

CONFIDENTIEL

	Aucun effet indésirable lié au MEX-CD1 n'a été constaté lors des études précliniques.<br>En l'absence de contact direct du MEX-CD1 avec le sang, les risques potentiels sont liés à la procédure d'hémodialyse et aux différents prélèvements sanguins veineux répétés.<br>Manifestations communes et bénignes associées à l'hémodialyse :<br>- Crampes musculaires<br>- Frissons, inconfort<br>- Nausées, vomissements<br>- Syndrome de déséquilibre<br>- Fatigue post-dialytique<br>- Céphalées<br>- Hémorragies – hématomes au niveau du point de ponction<br>Manifestations rares et graves survenant pendant la séance de dialyse :<br>- Troubles du rythme cardiaque<br>- Angor - Infarctus du myocarde<br>- Convulsions<br>- Réactions fébriles et chocs pyrogènes<br>- Manifestations de type allergique<br>- Hémolyse intravasculaire<br>Incidents et accidents survenant pendant la période interdialytique (extrêmement rares) :<br>- Infections<br>- Colite ischémique<br><br>**Contraintes**<br>Le cycle de 3 séances d'épuration extracorporelle étant prévu sur une semaine, le patient est contraint de devoir revenir sur le site (service de néphrologie de l'Hôpital Lyon Sud) de façon rapprochée pour la réalisation des séances.<br>Des visites de suivi sur site sont également prévus à 7 jours [± 2 jours] (V_1) et 28 jours [± 2 jours] (V_2) de la 3ème séance (S_3) d'EEC.<br>Enfin, une visite de fin d'étude (V_3) est prévue sur site 90 jours [± 7 jours] après la dernière séance S_3 d'Epuration ExtraCorporelle.<br><br>**Bénéfices**<br>Le principal bénéfice attendu pour les patients qui acceptent de participer à l'étude est une diminution significative de la charge en fer total de l'organisme, objectivée par une baisse de leur ferritinémie à l'issue du cycle de 3 séances d'Epuration ExtraCorporelle.
**Justification scientifique**	.<br><br>Les patients atteints d'hémopathies chroniques, d'hémoglobinopathies ou de pathologies d'origine immuno-hématologique nécessitant des transfusions répétées présentent un risque élevé de surcharge en fer. Celle-ci résulte de l'accumulation progressive du fer apporté par les concentrés érythrocytaires et, dans certaines situations, d'une érythropoïèse inefficace contribuant à une dérégulation de l'homéostasie martiale. Cette problématique concerne notamment les syndromes myélodysplasiques, les myélofibroses, certaines leucémies aiguës en rémission avant allogreffe, les thalassémies, les drépanocytoses, ainsi que diverses anémies congénitales ou acquises responsables d'une dépendance transfusionnelle prolongée.<br>Lorsque les capacités de transport du fer par la transferrine sont dépassées, du fer circulant non lié à la transferrine apparaît dans le plasma. Cette fraction de fer libre est particulièrement toxique car elle

CONFIDENTIEL

	favorise la production d'espèces réactives de l'oxygène responsables de lésions cellulaires et tissulaires. La surcharge martiale peut ainsi entraîner des atteintes cardiaques, hépatiques et endocriniennes, majorer le risque infectieux et contribuer à une diminution de la survie. Dans certaines situations spécifiques, notamment chez les patients candidats à une allogreffe de cellules souches hématopoïétiques, une surcharge en fer importante constitue également un facteur pronostique défavorable associé à une mortalité accrue et à une moins bonne survie post-greffe.<br><br>Le traitement de référence repose actuellement sur les chélateurs du fer, dont les plus utilisés sont le déférasirox et la défériprone. L'objectif de ces traitements est de réduire la charge martiale afin de prévenir les complications viscérales à long terme et d'améliorer le pronostic des patients polytransfusés. Toutefois, il n'existe pas de consensus clair sur le choix du traitement optimal, celui-ci devant être adapté à l'âge du patient, à ses comorbidités, à son contexte clinique, à la tolérance des traitements disponibles ainsi qu'à ses préférences.<br><br>Malgré leur efficacité démontrée, les traitements chélateurs présentent plusieurs limites. Leurs effets indésirables, notamment digestifs, peuvent compromettre l'observance thérapeutique et réduire leur efficacité en pratique courante. Par ailleurs, certains patients présentent une réponse insuffisante malgré l'utilisation de doses optimales ou de stratégies combinées. Enfin, le délai nécessaire à l'obtention d'une réduction significative de la charge martiale peut être incompatible avec certaines situations cliniques nécessitant une déplétion rapide du fer, notamment en amont d'une greffe de cellules souches hématopoïétiques.<br><br>De nombreuses études ont montré que la surcharge en fer est associée à une augmentation de la morbi-mortalité des patients polytransfusés, indépendamment de la pathologie sous-jacente. Les complications cardiaques, hépatiques, endocriniennes et infectieuses qu'elle induit constituent une cause majeure de détérioration clinique à long terme. Ainsi, malgré les progrès apportés par les traitements chélateurs conventionnels, le besoin de nouvelles stratégies thérapeutiques permettant une réduction plus rapide, plus efficace et mieux tolérée de la surcharge martiale demeure important. Dans ce contexte, les approches innovantes d'épuration extracorporelle du fer pourraient constituer une alternative ou un complément aux traitements actuellement disponibles.
**Objectifs et hypothèses de l'investigation clinique**	**Objectif principal**<br>Estimer l'effet du MEX-CD1 dans la chélation du fer à la fin de chacune des trois séances d'un cycle d'Epuration ExtraCorporelle.<br><br>**Objectifs secondaires**<br>1) Décrire la cinétique de la chélation du fer au cours de chacune des trois séances d'un cycle d'Epuration ExtraCorporelle<br>2) Estimer l'effet de l'Epuration ExtraCorporelle du MEX-CD1 sur les paramètres du bilan martial à court terme.<br>3) Décrire la tolérance des séances d'Epuration ExtraCorporelle par le MEX-CD1.<br>4) Évaluer l'effet de l'Epuration ExtraCorporelle avec le MEX-CD1 sur les besoins transfusionnels chez les patients présentant une dépendance transfusionnelle en CGR (applicable uniquement aux patients atteints de syndrome myélodysplasique, myélofibrose, hémoglobinose ou aplasie médullaire/BMF).<br>5) Décrire la faisabilité du traitement expérimental.

CONFIDENTIEL

<table>
<tr><td></td><td>
6) Décrire la tolérance globale de la séance ressentie par le patient.
7) Décrire l'évolution de la qualité de vie ressentie par le patient à distance du cycle de séances d'Epuration ExtraCorporelle.

Etude ancillaire No.1 :
Estimer la capacité de chélation du, cuivre, zinc, sélénium, plomb et cadmium sanguins d'un cycle de 3 séances d'Epuration ExtraCorporelle avec le MEX-CD1.

Etude ancillaire No.2[Δ] :
Estimer l'effet de la déplétion martial (fer) sur les programmes transcriptomiques des éléments matures circulants (différentiation).
[Δ] Sur la totalité des patients inclus et ayant bénéficié d'au moins deux séances d'Epuration ExtraCorporelle.

Hypothèse :
L'utilisation du MEX-CD1 pourrait permettre l'Epuration ExtraCorporelle (EEC) d'une quantité importante de fer chez des patients en surcharge. Pour vérifier cette hypothèse, nous proposons cet essai clinique pilote, monocentrique multisites HCL sur des patients qui présentent une surcharge en fer post-transfusionnelle, qui nécessitent une strétagie de déplétion en fer et sont intolérants ou réfractaires aux chélateurs.
</td></tr>
<tr><td>Critères de jugements</td><td>
Critère de jugement principal
Quantité de fer extrait mesurée au niveau du sac d'effluent (H_f) à la fin chaque séance d'Epuration ExtraCorporelle (S_1, S_2, S_3).

Critères de jugement secondaires
1) Quantité de fer mesurée sur la ligne d'effluent (H_1, H_2, H_f) au cours de chaque séance d'Epuration ExtraCorporelle (S_1, S_2, S_3)
2) Variations de fer, ferritine, CST mesurées entre le début (H_0) et la fin (H_f) de chaque séance d'Epuration ExtraCorporelle (S_1, S_2, S_3) ; Variations de fer, ferritine, CST mesurées entre le début de la 1ère séance (S_1H_0) et la fin de la 3ème séance (S_3H_f) ; Variations de fer, ferritine et CST mesurées entre le début de la fin de la dernière séance (S_1H_4) et V_1 (S_3 +J_7) / V_2 (S_3 +J_{28}) / V_3 (S_3 +J_{90})
3) Occurrence des évènements indésirables de tout type et notamment les Evènements d'Intérêt Particulier (EIP) de type : hypotension per procédure, réaction anaphylactoïde, effets indésirables au point de ponction veineux, troubles ioniques post-séance (sodium, potassium, calcium, magnésium). Pour chaque EIP, l'investigateur déterminera son imputabilité liée à la procédure (cf 11.1.4) et son intensité (cf 11.2.1) selon la CTCAE v5.0.
4) Dates de transfusion et nombre de CGR transfusés par patient chez les patients inclus présentant une dépendance trasnfusionnelle en CGR.
5) - Proportion de patients ayant réalisé la première séance d'Epuration ExtraCorporelle dans son entièreté.
- Proportion de patients ayant réalisé un cycle complet de 3 séances d'Epuration ExtraCorporelle consécutives.
- Ratio temps réel (heure) sous traitement expérimental / temps programmé (3h20).
- Volume total (litre) de sang traité (donnée fournie par l'équipement de dialyse).
</td></tr>
</table>

CONFIDENTIEL

	6) Cotation par EVA (Echelle Visuelle Analogique) [0 : normal – 10] sur la tolérance de la séance (S_1, S_2 et S_3) :<br>Asthénie/malaise ressenti : [0 : normal – 10 : très fatigué ou malaise intense après chaque séance]<br>Douleur ressentie : [0 : pas de douleur – 10 : douleur très forte après chaque séance]<br>7) Score à l'auto-questionnaire MOS SF-12 à l'inclusion, V_2 et V_3.<br><br>***Etude ancillaire n°1*** :<br>Quantités mesurées de, cuivre, zinc, sélénium, cadmium et plomb :<br>- à partir de prélèvements de sang veineux (tubes secs) à J_0 (inclusion), S_1H_0, S_1H_4, V_1 et V_2 ;<br>- à partir de la poche de dialysat avant le début de la séance (H0), après ajout du MEX-CD1 et homogénéisation, pour déterminer la quantité de métaux initialement présente dans le dialysat;<br>- à partir de prélèvements (1 par heure à H_1, H_2, H_f) au niveau de la ligne effluent pour calculer la cinétique d'extraction des métaux;à partir du sac d'effluent à la fin de la séance (H_f), après homogénéisation pour déterminer la quantité total (µg) de métaux extrait.<br>***Etude ancillaire n°2*** :<br>Mise en place d'une biocollection d'échantillons PBMC et plasma EDTA à partir de prélèvements sanguins réalisés à : inclusion, S_2, V_1 et V_3.
**Conception de l'investigation clinique**	Etude pilote non comparative, ouverte, sur une cohorte de 10 patients traités séquentiellement.<br>Etude pilote monocentrique, multisites HCL, prospective, descriptive.
**Informations sur le type d'investigation clinique**	L'investigation clinique est une catégorie 2.<br>Le critère de jugement principal est la quantité de fer mesurée, au niveau de la poche de dialysat (H_0), sur la ligne d'effluent (H_1, H_2, H_f), et du sac d'effluent (H_f) au cours chaque séance d'Epuration ExtraCorporelle (S_1, S_2, S_3).
**Informations sur les participants**	**Critères d'inclusion**<br>- Patients âgés de 18 ans et plus.<br>- Ferritinémie >1000 µg/L sur 2 mesures successives à 2 semaines d'intervalle ,secondaire à des supports transfusionnels dans le cadre d'une hémopathie, une hémoglobinose ou toute autre pathologie d'origine immuno-hématologique.<br>- Patients présentant un taux de plaquettes ≥ 50 giga/L.<br>- Patients présentant une intolérance ou une contre-indication à un traitement chélateur oral /sous cutané ou une infficacité réelle ou attendue d'un traitement chélateur aux doses optimales.<br>- Patient capable de comprendre et de se conformer au protocole, ayant signé le consentement éclairé.<br>- Patient affilié à un régime de sécurité sociale ou un régime similaire.<br><br>**Critères de non-inclusion**<br>- Patients présentant une hémochromatose primitive (coefficient de saturation de la transferrine CS-Tf >45%).<br>- Patient présentant une hyperferritinémie >1000 µg/L dans le cadre d'un syndrome métabolique, d'une pathologie inflammatoire chronique ou tout autre pathologie ne rentrant pas dans le cadre

CONFIDENTIEL

	d'une hémopathie, une hémoglobinose ou toute autre pathologie d'origine immuno-hématologique.<br>- Patient présentant une contre-indication liée à l'utilisation du MEX-CD1 : poids < 50 kg, carence en fer.<br>- Patient présentant une allergie connue ou contre-indication à l'héparine ou au citrate.<br>- Patient sous azacitidine (monothérapie ou en association) ou tout autre chimiothérapie (ou considéré comme tel) administré en schéma répété/mensuels pour une leucémie aigüe, syndrome myélodysplasique ou une myélofibrose. Les patients sous inhibiteurs de JAK1/2 peuvent être inclus sous réserve que le traitement ait été introduit depuis au moins 3 mois et que la dose n'ait pas été modifié dans les 4 dernières semaines avant l'inclusion.<br>- Patient ayant reçu un traitement par luspatercept ou érythropoïétine (EPO) au cours du mois précédant l'inclusion<br>- Patient avec indication pour un traitement par allogreffe de cellules souches dans les 4 semaines après son inclusion dans l'étude.<br>- Patient présentant une allergie connue aux crustacés (MEX-CD1 contenant du chitosan d'origine animale) ou à l'un des autres composant du MEX-CD1.<br>- Patients présentant un abord vasculaire périphérique difficile ou à préserver (notamment en cas de projet de dialyse).<br>- Patient ayant une infection connue telle que la tuberculose, au virus d'immunodéficience humaine (VIH) ou maladie liée au syndrome d'immunodéficience acquise (SIDA).<br>- Patient ayant un test positif pour l'antigène de surface de l'hépatite B et/ou l'ARN de l'hépatite C (si sérologie positive)<br>- Patient participant à une autre recherche interventionnelle qui pourrait interférer avec les résultats de l'étude.<br>- Patient majeur sous mesure de protection légale (tutelle, curatelle) ou hors d'état d'exprimer leur consentement.<br>- Patient faisant l'objet de soins psychiatriques.<br>- Patient privé de liberté par une décision judiciaire ou administrative.<br>- Femme enceinte (Test de grossesse adéquat par dosage de la gonadotrophine chorionique humaine, hCG pour les femmes en âge de procréer) ou allaitante.<br><br>L'IC MEXIRON est une étude pilote visant à estimer la faisabilité logistique et la tolérance de l'intervention proposée et obtenir des premières données cliniques et biologiques qui seraient nécessaires pour calculer la taille d'échantillon d'une future étude de plus grande envergure.<br>Nous avons prévu d'inclure un nombre total de 10 sujets traités.
**Critères de sortie prématurée**	- Survenue d'un événement indésirable de type de réaction anaphylactique ou anaphylactoïde pendant une des séances.<br>- Abord vasculaire difficile observé et contre-indiquant la réalisation de la séance d'Epuration ExtraCorporelle (selon jugement de l'investigateur et avis spécialisé).<br>- Retrait de consentement du patient.
**Procédures cliniques <u>ajoutées</u> du fait de l'investigation**	**Traitements**

CONFIDENTIEL

	- Anticoagulation préventive aux séances d'Epuration Extracorporelle<br>- Supplémentation en oligoéléments (gluconate de Zinc, gluconate de Cuivre et Sélénite de sodium)<br>**Consultations**<br>- Visite et examen clinique à l'inclusion (J0)<br>- Visites et examens cliniques de suivi (V_1, V_2 et V_3)<br>**Bilans biologiques**<br>- Prélèvements sanguins veineux pour le dosage du fer, CST, ferritine, CRP, ionogramme sanguin (sodium, potassium, calcium, magnésium) et NFS en début (H_0) et fin (H_f) de chaque séance d'Epuration Extracorporelle (S_1, S_2, S_3) ainsi que lors des visites de suivi V_1, V_2 et V_3.<br>- Monitoring TAS, TAD, fréquence cardiaque et température durant toute la durée des séances d'Epuration Extracorporelle : H_{0+2min}, H_{0+3min}, H_1, H_2 et H_f avant débranchement.<br>**Prélèvements sanguins (étude ancillaire No.1)**<br>- Prélèvement veineux (1 tube d'analyse des oligo-éléments 6 mL) pour le dosage du cuivre zinc, plomb, cadnium et sélénium lors de la visite d'inclusion (J_0), au début et à la fin de la séance d'Epuration ExtraCorporelle 1 (S_1H_0, S_1H_f), de la visite de suivi (V_1) et de la visite de suivi (V_2)<br>**Prélèvements du liquide de dialyse (étude ancillaire No.1)**<br>- Prélèvement (1 tube à centrifuger stérile 15 mL) au niveau de la poche dialysat au début (H_0) de chaque séance d'Epuration ExtraCorporelle.<br>- Prélèvements (1 tube à centrifuger stérile 15 mL) sur la ligne de l'effluent à H_1, H_2, H_f au cours de chaque séance d'Epuration ExtraCorporelle.<br>- Prélèvement (2 tubes à centrifuger stérile 15 mL) au niveau du sac de l'effluent à la fin (H_f) de chaque séance d'Epuration ExtraCorporelle.<br>**Questionnaire – échelle**<br>- Auto-passation d'une EVA en fin de séance d'Epuration ExtraCorporelle (S_1, S_2, S_3)<br>- Auto-questionnaire de qualité de vie MOS SF-12 à l'inclusion (J_0), V_2 et V_3<br>**Constitution d'une biocollection (étude ancillaire No.2)**<br>- Visite d'inclusion (J_0) : prélèvements sanguins veineux (3 tubes EDTA 4 mL et 1 tube sec 4 mL) ;<br>- EEC-séance S_2 : prélèvements sanguins veineux (3 tubes EDTA 4 mL et 1 tube secs 4 mL) ;<br>- Visite de suivi (V_1) : prélèvements sanguins veineux (3 tubes EDTA 4 mL et 1 tube sec 4 mL) ;<br>- Visite fin d'étude (V_3) : prélèvements sanguins veineux (3 tubes EDTA 4 mL et 1 tube sec 4 mL).
**Liste des lieux de recherche**	Hôpital Lyon Sud :<br>- Service d'Anesthésie - Réanimation - Médecine Intensive.<br>- Service d'Hématologie Clinique.<br>- Service de Néphrologie, dialyse et nutrition rénale
**Plan de surveillance**	L'IC MEXIRON est conduite en application de l'article 80 du RDM. Les investigateurs devront notifier immédiatement au promoteur tous

CONFIDENTIEL

	les EIG et défectuosités qui auraient pu déboucher sur un EIG (cf. chapitre « Evaluation de la sécurité » ci-dessous).<br>Un Attaché de Recherche Clinique (ARC) mandaté par le promoteur s'assure de la bonne réalisation de l'investigation clinique, du recueil des données générées par écrit, de leur documentation, enregistrement et rapport, en accord avec les POS mises en application au sein de la DRS des Hospices Civils de Lyon et conformément aux Bonnes Pratiques Cliniques ainsi qu'aux dispositions législatives et réglementaires en vigueur.
**Considérations statistiques**	Cette étude de phase 1 permettra avec 13 patients d'évaluer l'effet moyen du MEX-CD1 sur les différents paramètres biologiques des patients au cours des différentes séances, ainsi que la sécurité du dispositif.<br>Il n'y a pas de nombre de sujet nécessaire à calculer.
**Mesures pour réduire les biais**	Le suivi de tous les patients inclus est strictement identique et le recueil de données sera standardisé au moyen d'un cahier d'observation électronique (eCRF) pour éviter biais de sélection avec d'éventuels patients perdus de vue et réduire le risque d'attrition de données.
**Gestion des données**	Les données de l'étude sont recueillies dans un eCRF compliant FDA, CNIL et RGPD. Cet eCRF, spécifique à l'étude, a été développé par un data manager des Hospices Civils de Lyon sur le logiciel Ennov Clinical® 82.50.<br>Cette étude entre dans le cadre de la « Méthodologie de Référence » (MR-001) en application des dispositions de l'article 54 alinéa 5 de la loi n°78-17 du 6 janvier 1978 modifiée relative à l'informatique, aux fichiers et aux libertés. Ce changement a été homologué par décision du 5 janvier 2006 et modifiée le 21 juillet 2016. Les Hospices Civils de Lyon, promoteur de l'étude, ont signé un engagement de conformité à cette « Méthodologie de Référence ».
**Gestion des modifications au plan d'investigation clinique**	En cas de modification substantielle apportée au protocole par l'investigateur, elle sera approuvée par le promoteur. Ce dernier devra obtenir préalablement à sa mise en œuvre un avis favorable du CPP et une autorisation de l'ANSM, dans le cadre de leurs compétences respectives.
**Ecarts au protocole**	Tout écart significatif au protocole sera relevé et fera l'objet d'une action correctrice et sera pris en compte pour l'analyse finale.
**Responsabilité concernant le DM**	Le dispositif médical MEX-CD1 de classe III est fabriqué par la société MexBrain (13 avenue Albert Einstein, 69100 VILLEURBANNE). Le dispositif sera mis à disposition du service de réanimation dès la mise en place de l'étude. La société MexBrain livrera le matériel à l'Unité des Dispositifs Médicaux Stériles (service de Pharmacie – Hôpital Lyon Sud) au fil des inclusions et par anticipation aux séances de traitement planifiées. L'Unité des Dispositifs Médicaux Stériles est responsable de la comptabilité des distributions des produits, des demandes de réapprovisionnement, de la vérification de la date de péremption et des modalités de conservation du produit. L'utilisation du dispositif est sous la responsabilité de l'investigateur principal de l'étude. En cas de dysfonctionnement constaté du dispositif, celui-ci est retourné à la société MexBrain tout comme les dispositifs non utilisés en fin d'étude ou arrivant à expiration.
**Déclaration de conformité**	Considérant l'article 62 du Règlement européen du Dispositif médical (RDM) 2017/745 sur les dispositions de conformité régissant les investigations cliniques,le promoteur et l'investigateur, en signant ce protocole, s'engagent à ce que l'investigation clinique soit conduite :<br>- Conformément au Règlement européen du Dispositif médical (RDM) [(UE) 2017/745 relatif aux dispositifs médicaux] ainsi qu'aux dispositions prises pour son application en France ;<br>- Conformément au protocole ;

CONFIDENTIEL

	- Conformément aux recommandations du *Medical Device Coordination Group* (MDCG) applicables concernant les investigations cliniques et notamment concernant le contenu de la Brochure pour l'Investigateur ;<br>- Conformément aux BPC ICH-E6 dans leur dernière version en vigueur pour les parties applicables ;<br>- Conformément à la déclaration d'Helsinki de l'Association médicale mondiale sur les principes éthiques applicables à la recherche médicale impliquant des êtres humains ;<br>- Conformément au Règlement Général sur la Protection de Données (RGPD) et conformément à la Loi Informatique et Liberté (LIL) concernant le traitement des données ;<br>- Conformément aux dispositions législatives et réglementaires actuellement en vigueur en France ;<br>- Conformément à la norme ISO 9001, certification du promoteur depuis 2018.<br>Considérant ces engagements de respect de conformité, nous concluons que le niveau de conduite de l'étude sera mené avec un niveau équivalent à la norme ISO 14155 concernant la sécurité, la qualité et la robustesse des données..
**Procédure de consentement**	Les patients sont informés de façon complète et loyale, en des termes compréhensibles, des objectifs et des contraintes de l'étude, des risques éventuels encourus, des mesures de surveillance et de sécurité nécessaires, de leurs droits de refuser de participer à l'IC ou de la possibilité de se rétracter à tout moment.<br>Toutes ces informations figurent sur un formulaire d'information et de consentement remis au patient. Le consentement libre, éclairé et écrit du patient est recueilli par l'investigateur, ou un médecin qui le représente avant l'inclusion définitive dans l'étude. Une copie du formulaire d'information et de consentement signé par les deux parties est remise au patient, l'investigateur en conserve aussi un exemplaire original dans le classeur de l'étude après l'avoir scanné et archivé dans le dossier médical informatisé patient.
**Sécurité**	De la première visite (inclusion J_0) jusqu'à la fin de l'étude et jusqu'à leur résolution, l'investigateur recherche, rapporte, enregistre, traite et évalue dans l'eCRF :<br>a. Tout événement indésirable défini dans le protocole comme déterminant pour l'évaluation des résultats de l'investigation clinique ;<br>b. Tout évènement indésirable grave ;<br>c. Toute défectuosité d'un DM qui aurait pu déboucher sur un événement indésirable grave en l'absence de mesures appropriées ou d'une intervention, ou si les circonstances avaient été moins favorables ;<br>d. Tout nouvel élément concernant un événement visé aux points *a)* à *c)*<br><br>Les événements suivants ne sont pas considérés comme des événements indésirables (ils ne sont donc pas à collecter dans le eCRF) :<br>- Admission pour raisons sociale ou administrative ;<br>- Hospitalisation prédéfinie par le protocole ;<br>- Passage en hôpital de jour programmé dans le cadre du suivi de la pathologie étudiée ou d'une maladie intercurrente déjà connue à l'inclusion.<br><br>**Défectuosité du dispositif médical expérimental**

CONFIDENTIEL

	Tout défaut en matière d'identité, de qualité, de durabilité, de fiabilité, de sécurité ou de performances du dispositif médical expérimental faisant l'objet d'une investigation, y compris tout dysfonctionnement, toute erreur d'utilisation ou tout défaut dans les informations fournies par le fabricant.<br>**Evènement d'intérêt particulier**<br>- Défectuosité d'un dispositif causant un EIG ;<br>- Défectuosité d'un dispositif qui aurait pu déboucher sur un événement indésirable grave en l'absence de mesures appropriées ;<br>- Réaction anaphylactique, manifestations de type allergiques quel que soit le grade pendant les séances ;<br>- Réactions fébriles supérieure à 38,5°C et chocs pyrogènes pendant les séances;<br>- Hémolyse intravasculaire ;<br>- Allo-immunisation ;<br>- Événements hémorragiques dans les 48h suivant la séance d'Epuration ExtraCorporelle et nécessitant une transfusion ;<br>- Changement de grades CTCAE des cytopénies, plus particulièrement de l'anémie ;<br>- Troubles du rythme cardiaque ;<br>- Infections nécessitant une antibiothérapie oral et/ou IV ;<br>- Hypotension artérielle<br>- Colite ischémique.<br>**Restriction des EIG**<br>- Hospitalisation pour traitement médical ou chirurgical programmé avant la recherche ;<br>- Admission aux urgences de moins de 24 heures (non liée au traitement) ;<br>- Neutropénie fébrile (grade 3) ;<br>Transfusions liées au soin courant et à la prise en charge du patient habituelle.
**Suivi des participants après la fin de l'investigation**	Après la fin de l'investigation clinique, les patients seront pris en charge selon les modalités habituelles.
**Prise en charges des participants**	Aucun soin supplémentaire n'est nécessaire du fait de la participation du patient à l'investigation clinique.
**Rapport d'investigation clinique**	Conformément à la réglementation, le rapport final d'investigation clinique sera réalisé un an après la fin de la recherche.<br>Les communications et rapports scientifiques correspondant à cette étude seront réalisés sous la responsabilité de l'investigateur principal de l'investigation clinique avec l'accord des investigateurs associés. Les co-auteurs du rapport et des publications seront les investigateurs et les cliniciens impliqués, au prorata de leur contribution à l'étude, ainsi que le méthodologiste, le biostatisticien et les chercheurs associés.<br>Les règles de publications suivront les recommandations internationales (N Engl J Med, 1997; 336 :309-315).<br>L'étude sera enregistrée sur un registre des essais cliniques en libre accès (clinicaltrials.gov) avant l'inclusion du premier patient.
**Durée de l'investigation clinique**	Durée de la période d'inclusion : 12 mois<br>Durée de la participation pour chaque sujet : 15 semaines<br>Durée totale de l'investigation clinique : 16 mois<br>Date réelle de début de la recherche : 06/01/2026<br>Date théorique de fin de la recherche : 06/05/2027

SOMMAIRE

1 INFORMATIONS GENERALES ... 17
1.1 Titre ... 17
1.2 Identifiants du projet et historique des mises à jour ... 17
1.3 Promoteur ... 17
1.3.1 Identité ... 17
1.3.2 Signature du protocole au nom du Promoteur ... 17
1.3.3 Responsables de la recherche au niveau du Promoteur ... 17
1.3.4 Responsable de la vigilance des essais au niveau du Promoteur ... 17
1.4 Investigateurs ... 18
1.4.1 Investigateur principal ... 18
1.4.2 Investigateurs des sites associés ... 18
1.5 Coordinateur scientifique ... 18
1.6 Coordination d'étude ... 18
1.7 Scientifiques associés ... 19
1.8 Méthodologistes ... 19
1.9 Data-Management ... 20
1.10 Biostatisticien ... 20
1.11 Pharmacien ... 20
1.12 Comité de Surveillance Indépendant ... 20
1.13 Modalités de financement de l'investigation clinique ... 21
1.14 Assurance ... 21
1.15 Scénario général de l'investigation clinique ... 21
2 DISPOSITIF MEDICAL ... 21
2.1 Données d'identification et description du DM ... 21
2.1.1 Description du DM ... 21
2.1.2 Procédures d'investigation ... 22
2.1.3 Procédures additionnelles ... 23
2.1.4 Bénéfices escomptés du DM ... 25
2.2 Caractéristiques techniques et fonctionnelles du DM évalué ... 26
2.3 Risques et bénéfices du DM évalué ... 26
2.3.1 Bénéfices ... 26
2.3.2 Contraintes ... 26
2.3.3 Risques potentiels ... 27
3 JUSTIFICATION SCIENTIFIQUE ... 28
4 OBJECTIFS ET CRITERES DE JUGEMENT ... 31
5 CONCEPTION DE L'INVESTIGATION CLINIQUE ... 34
5.1 Information sur le type d'investigation clinique ... 34
5.2 Information sur le dispositif médical ... 34
5.3 Mesures pour réduire les biais ... 34
5.4 Informations sur les participants ... 35
5.4.1 Critères d'inclusion ... 35
5.4.2 Critères de non-inclusion ... 35
5.4.3 Critères de sortie prématurée ... 36
5.4.4 Taille de la population et modalités de recrutement ... 36
6 ORGANISATION GENERALE DE L'ETUDE ... 37
6.1 Calendrier de l'étude ... 37

6.2 Schéma général et tableau récapitulatif ... 37
6.3 Déroulement de l'étude ... 37
6.3.1 Screening – inclusion (J_0) ... 37
6.3.2 Déroulement d'une séance d'Epuration ExtraCorporelle (S_1, S_2, S_3) ... 40
6.3.3 Visites de suivi (V_1, V_2) ... 41
6.3.4 Visite de fin de la recherche (V_3) ... 42
6.4 Procédures cliniques ... 42
6.5 Collection d'échantillon biologique (étude ancillaire No. 2) ... 43
6.6 Plan de surveillance / Contrôle qualité ... 44
7 CONSIDERATIONS STATISTIQUES ... 44
7.1 Justification de la taille de l'échantillon ... 44
7.2 Population d'analyse ... 45
7.3 Méthode statistiques ... 45
7.3.1 Critère de jugement principal ... 45
7.3.2 Critères de jugements secondaires ... 45
7.4 Analyses intermédiaires ... 46
7.5 Méthode de prise en compte des données manquantes ... 46
7.6 Gestion des modifications apportées au plan d'analyse ... 46
7.7 Responsable des analyses ... 46
8 DROITS D'ACCES AUX DONNEES ET DOCUMENT SOURCES ... 46
8.1 Accès aux données ... 46
8.2 Documents sources ... 47
8.3 Confidentialité des données / CNIL ... 47
8.4 Conformité à la Norme IS0 14155 ... 47
9 GESTION ET CONSERVATION DES DONNEES ... 48
9.1 Gestion des modifications ... 48
9.2 Ecarts au protocole ... 48
9.3 Cahier d'observation (électronique) ... 48
9.4 Gestion des données ... 49
9.5 Plan de data management ... 50
9.6 Archivage ... 50
10 CONSIDERATIONS ETHIQUES ... 51
10.1 Période d'exclusion et interdiction de participer à une autre étude clinique ... 51
10.2 Indemnisation des sujets et inscription au fichier national des personnes se prêtant à une recherche interventionnelle ... 51
10.3 Remboursement des sujets ... 51
10.4 Autorités compétentes ... 51
10.5 Modifications substantielles ... 51
11 RESPONSABILITES CONCERNANT LE DISPOSITIF ... 51
11.1 Fabrication ... 51
11.2 Conditionnement et étiquetage ... 51
11.3 Gestion du dispositif ... 52
11.4 Dispensation du dispositif ... 52
11.5 Stockage ... 53
11.6 Contrôle de l'accès au dispositif médical expérimental ... 53
11.7 Suivi de l'utilisation dans le cadre de l'investigation ... 53

CONFIDENTIEL

11.8 Retour des dispositifs médicaux expérimentaux inutilisés, arrivés à expiration ou présentant un dysfonctionnement 53
12 DECLARATION DE CONFORMITE 53
13 ÉVALUATION DE LA SÉCURITÉ 53
13.1 Définitions de la vigilance des investigations cliniques 53
13.1.1 Evénement indésirable (EI) 53
13.1.2 Effet indésirable du dispositif 54
13.1.3 Evénement ou effet indésirable grave 54
13.1.4 Défectuosité du dispositif médical expérimental 54
13.1.5 Classification des niveaux de causalité 54
13.1.6 Effet indésirable grave inattendu 55
13.2 Responsabilité de l'investigateur 55
13.2.1 Modalités de détection et de recueil des événements indésirables 55
13.2.2 Notification immédiate des Evénements Indésirables Graves (EIG) au promoteur 56
13.2.3 Evaluation de la causalité des EI 57
13.2.4 Période de notification des EIG relié à la procédure d'investigation 57
13.2.5 Evènement d'intérêt particulier 57
13.2.6 Restriction des EIG (formulaire de notification des EIG à ne pas envoyer au secteur vigilance mais à recueillir uniquement dans l'eCRF au plus tard à la fin de participation à l'étude) : 57
13.3 Responsabilités du promoteur 57
13.3.1 Déclaration aux autorités compétentes 57
13.3.2 Documents d'évaluation du risque pour l'évaluation du caractère attendu/inattendu 58
13.3.2.1 Risques liés au dispositif médical expérimental 58
13.3.2.2 Risques liés aux procédures d'investigation (pour la mise en oeuvre du dispositif médical expérimental) 58
13.3.2.3 Description des effets indésirables reliés aux autres procédures additionnelles 59
13.3.2.4 Description des événements indésirables reliés à la maladie étudiée 59
13.3.2.5 Description des événements indésirables reliés au soin 59
13.3.2.6 Description des événements indésirables à ne pas déclarer à l'ANSM 59
13.4 Comité de surveillance indépendant 60
14 SUIVI DES PARTICIPANTS APRES LA FIN DE L'IC 60
14.1 Arrêt de participation d'une personne à la recherche 60
14.2 Arrêt d'une partie ou de la totalité de la recherche 60
15 PRISE EN CHARGE DES PARTICIPANTS 61
16 RAPPORT D'INVESTIGATION CLINIQUE 61
17 REFERENCES BIBLIOGRAPHIQUES* 61
18 LISTE DES ANNEXES 63

CONFIDENTIEL

1 INFORMATIONS GENERALES

1.1 Titre

MEXIRON : Faisabilité, tolérance et efficacité de l'épuration extracorporelle du fer chez des patients présentant une surcharge martiale nécessitant une stratégie de déplétion associée à une intolérance, une contre-indication ou une inefficacité à un traitement chélateur oral ou sous cutané.

1.2 Identifiants du projet et historique des mises à jour

Code promoteur : 69HCL23_0102
N°ID-RCB / EUDAMED : 2024-A02473-44
Avis favorable du CPP Ouest III le : 13/03/2025
Autorisation de l'ANSM le : 10/04/2025

**Historique des versions**		
**Version**	**Date**	**Motif de la mise à jour**
1	22/01/2025	Rédaction initiale
2	06/03/2025	Modifications à la demande du CPP
3	24/03/2025	Modifications à la demande de l'ANSM
4	12/06/2025	Amendement n°1
4.1	08/08/2025	Amendement n°1 - Réponse au CI ANSM
5	12/01/2026	Amendement n°2
6	31/03/2026	Amendement n°3
7	28/07/2026	Amendement n°4

1.3 Promoteur

1.3.1 Identité

Hospices Civils de Lyon
BP 2251
3 Quai des Célestins
69229 LYON Cedex 02

1.3.2 Signature du protocole au nom du Promoteur

Alexandre PACHOT, Directeur de la Recherche en Santé
Hospices Civils de Lyon, Direction de la Recherche en Santé, Siège Administratif, BP 2251, 3 Quai des Célestins, 69229 LYON Cedex 02
Tél. : 04 72 40 68 52

1.3.3 Responsables de la recherche au niveau du Promoteur

Thierry HEREMBERT, Responsable secteur promotion interne
Hospices Civils de Lyon, Direction de la Recherche en Santé, Siège Administratif, BP 2251, 3 Quai des Célestins, 69229 LYON Cedex 02
Tél. : 04 72 40 70 87

1.3.4 Responsable de la vigilance des essais au niveau du Promoteur

Emeline BLANC, Pharmacien référent
Hospices Civils de Lyon, Direction de la Recherche en Santé, Siège Administratif, BP 2251, 3 Quai des Célestins, 69229 LYON Cedex 02
Tél. : 04 72 40 68 13

CONFIDENTIEL

1.4 Investigateurs

1.4.1 Investigateur principal

Dr Auguste DARGENT
Service d'Anesthésie - Réanimation - Médecine Intensive
Hôpital Lyon Sud
Hospices Civils de Lyon
165 Chemin du Grand Revoyet
69310 OULLINS-PIERRE-BENITE
Tél. : 04 78 86 20 06
Email : auguste.dargent@chu-lyon.fr

1.4.2 Investigateurs des sites associés

Dr Maël HEIBLIG
Service d'Hématologie Clinique (centre recruteur)
Hôpital Lyon Sud
Hospices Civils de Lyon
165 Chemin du Grand Revoyet
69310 OULLINS-PIERRE-BENITE
Tél. : 04 78 86 22 10
Email : mael.heiblig@chu-lyon.fr

Dr Bastien BERGER
Service de Nephrologie - Hémodialyse (centre non recruteur)
Hôpital Lyon Sud
Hospices Civils de Lyon
165 Chemin du Grand revoyet
69310 OULLINS-PIERRE-BENITE
Tél. : 04 78 87 87 19
Email : bastien.berger@chu-lyon.fr

1.5 Coordinateur scientifique

Pr Bernard ALLAOUCHICHE
Service d'Anesthésie - Réanimation - Médecine Intensive
Hôpital Lyon Sud
Hospices Civils de Lyon
165 Chemin du Grand Revoyet
69310 OULLINS-PIERRE-BENITE
Tél : 04 78 86 23 42
Email : bernard.allaouchiche@chu-lyon.fr

1.6 Coordination d'étude

Marine ALVES FERREIRA
Service Recherche et Epidémiologie Cliniques
Pôle Santé Publique
Hôpital Lyon Sud
Hospices Civils de Lyon
165 Chemin du Grand Revoyet
69310 OULLINS-PIERRE-BENITE
Tél. : 04 78 86 57 25
Email : marine.alves-ferreira@chu-lyon.fr

Laurent VILLENEUVE
Service Recherche et Epidémiologie Cliniques
Pôle Santé Publique
Hôpital Lyon Sud
Hospices Civils de Lyon
165 Chemin du Grand Revoyet
69310 OULLINS-PIERRE-BENITE
Tél. : 04 78 86 45 36
Email : laurent.villeneuve@chu-lyon.fr

CONFIDENTIEL

1.7 Scientifiques associés

Dr Maxime ESPI
Service de Néphrologie – Hémodialyse
Hôpital Lyon Sud
Hospices Civils de Lyon
165 Chemin du Grand revoyet
69310 OULLINS-PIERRE-BENITE
Tél. : 04 78 87 87 19
Email : maxime.espi@chu-lyon.fr

Dosage des métaux lourds [Etude ancillaire No. 1]

Dr François PARANT
CBAPS - Biochimie et Biologie Moléculaire
Laboratoire d'Analyse de Traces et Métaux Toxiques
165 chemin du Grand Revoyet
69495 PIERRE-BÉNITE
Tél : 04 78 86 38 63
Email : francois.parant@chu-lyon.fr

Pr Olivier TILLEMENT
Institut Lumière Matière
UMR5306 - UCBL - CNRS
10 rue Ada Byron
69622 VILLEURBANNE CEDEX
Tél. : 07 87 09 25 62
Email : olivier.tillement@univ-lyon1.fr

Axel AIGLE
MexBrain
13 avenue Albert Einstein
69100 VILLEURBANNE
Tél. : 01 46 41 84 90
Email : axel.aigle@mexbrain.com

1.8 Méthodologistes

Julien BERTHILLER
Cellule d'Expertise Dispositifs Médicaux Innovants
Direction de l'Innovation
Hospices Civils de Lyon
3 Quai des Célestins
69229 LYON Cedex 02
Tél. : 04 27 85 63 01
Email : julien.berthiller@chu-lyon.fr

Laure HUOT
Cellule d'Expertise Dispositifs Médicaux Innovants
Direction de l'Innovation
Hospices Civils de Lyon
3 Quai des Célestins
69229 LYON Cedex 02
Tél. : 04 72 11 57 62
Email : laure.huot@chu-lyon.fr

Dr Sylvie BIN-DOREL
Service Recherche et Epidémiologie Cliniques
Pôle Santé Publique
Hospices Civils de Lyon
162 Avenue Lacassagne
69424 LYON Cedex 03
Tél. : 04 72 11 53 66
Email : sylvie.bin@chu-lyon.fr

CONFIDENTIEL

1.9 Data-Management

Dr Sabine MAINBOURG
Pôle Santé Publique - Unité Bases Cliniques et Epidémiologiques
Hospices Civils de Lyon
162 Avenue Lacassagne
69424 LYON Cedex 03
Tél. : 06 19 51 36 15
Email : sabine.mainbourg@chu-lyon.fr

1.10 Biostatisticien

Dr Muriel RABILLOUD
Service de Biostatistiques et Bioinformatique
Pôle Santé Publique - Hospices Civils de Lyon
162 Avenue Lacassagne
69424 LYON Cedex 03
Tél. : 04 72 11 57 22
Email : muriel.rabilloud@chu-lyon.fr

Lorraine BERNARD
Service de Biostatistiques et Bioinformatique
Pôle Santé Publique - Hospices Civils de Lyon
162 Avenue Lacassagne
69424 LYON Cedex 03
Tél. : 04 72 11 57 91
Email : lorraine.bernard01@chu-lyon.fr

Dr Maxime BONJOUR
Service de Biostatistiques et Bioinformatique
Pôle Santé Publique - Hospices Civils de Lyon
162 Avenue Lacassagne
69424 LYON Cedex 03
Tél. : 04 72 11 51 01
Email : maxime.bonjour@chu-lyon.fr

1.11 Pharmacien

Dr Cédric BESNIER
Service de Pharmacie
Unité des Dispositifs Médicaux Stériles
Hôpital Lyon Sud
Hospices Civils de Lyon
165 Chemin du Grand Revoyet
69310 OULLINS-PIERRE-BENITE
Tél. : 04 78 86 23 10
Email : cedric.besnier@chu-lyon.fr

1.12 Comité de Surveillance Indépendant

Dr Tristan DELORY
Délégation à la Recherche Clinique et à l'Innovation
Centre Hospitalier Annecy Genevois
1 Avenue de l'hôpital
74370 EPAGNY-METZ-TESSY
Tél. : 04 56 49 72 46
Email : tdelory@ch-annecygenevois.fr

Dr Jean-Pierre QUENOT
Service de Médecine Intensive – Réanimation
CHU Dijon Bourgogne
Hôpital François Mitterrand
14 rue Gaffarel
BP 7790
21079 DIJON Cedex
Tél. : 03 80 29 36 85
Email : jean-pierre.quenot@chu-dijon.fr

Dr Mathieu MEUNIER
Service d'Hématologie Clinique
Hôpital Michallon - CHU Grenoble Alpes
Boulevard de la Chantourne
38700 LA TRONCHE
Tél. : 04 76 76 54 52
Email : mmeunier2@chu-grenoble.fr

CONFIDENTIEL

1.13 Modalités de financement de l'investigation clinique

Cette étude est financée conjointement par la société MexBrain (13 avenue Albert Einstein - 69100 VILLEURBANNE) et par l'organisme Lyon Biopôle-HCL.

1.14 Assurance

Le promoteur a souscrit pour toute la durée de l'investigation clinique une assurance garantissant sa propre responsabilité civile ainsi que celle de tout médecin impliqué dans la réalisation de l'investigation clinique. Il assurera également l'indemnisation intégrale des conséquences dommageables à la recherche pour la personne qui s'y prête et ses ayants droit, sauf preuve à sa charge que le dommage n'est pas imputable à sa faute ou à celle de tout intervenant, sans que puisse être opposé le fait d'un tiers ou le retrait volontaire de la personne qui avait initialement consenti à se prêter à la recherche.

Le contrat d'assurance a été souscrit avant le démarrage de l'investigation clinique auprès de la Société *Lloyd's Insurance Company*, Bastion Tower, Marsveldplein 5, 1050 Bruxelles, sous le numéro HSLCET24002.

1.15 Scénario général de l'investigation clinique

Etude pilote non comparative, ouverte, sur un groupe unique de 10 patients traités séquentiellement.
Etude monocentrique multisites HCL prospective descriptive.

2 DISPOSITIF MEDICAL

2.1 Données d'identification et description du DM

2.1.1 Description du DM

Le dispositif médical MEX-CD1 est basé à la fois sur le principe de la dialyse et la science des polymères pour l'extraction des métaux du sang. Le composant actif MEX-CD1 est composé de DOTA-GA lié de manière covalente à un chitosane partiellement (c'est-à-dire ~40%) acétylé. En raison de leur taille (masses moléculaires >100 kDa), les composants actifs ne peuvent pas traverser la membrane de dialyse et ne pénètrent donc pas dans la circulation systémique.

Le gradient de concentration entre les cations de fer libres ou ceux associés à de petites espèces chimiques présentes dans le sang et ceux présents dans le liquide de dialyse induit un flux de ces cations du sang vers le liquide de dialyse. Il en résulte une clairance progressive du fer du sang et un enrichissement concomitant du liquide de dialyse en cations de fer. En dialyse conventionnelle, le flux de diffusion s'arrête lorsque la concentration de cations métalliques diffusibles est la même dans le sang et dans le liquide de dialyse. Dans le traitement MEX-CD1, les cations de fer qui ont traversé la membrane sont capturés par les polymères ultra-chélatant (MEX-CD1) et ne sont plus disponibles pour la diffusion à travers la membrane, ce qui permet de maintenir un gradient de cations de fer important et donc de poursuivre le processus de diffusion/purification.

La solution d'hémodialyse à base de MEX-CD1 est un produit non invasif fabriqué à partir de matières premières d'origine animale qui sont rendues non viables. Cette solution est contenue dans un flacon de 50 mL. MEX-CD1 est formulé à une concentration de 10 g/L dans le liquide d'injection standard. Ce dispositif médical est de classe III en vertu du règlement 2017/745 sur les dispositifs médicaux (RDM) conformément aux règles 3 et 18.

La solution polymérique chélatrice MEX-CD1 utilise un chélateur (DOTA-GA) et un biopolymère (chitosane) qui ont déjà été approuvés pour une utilisation clinique. De plus, l'absence de contact direct entre la circulation sanguine et la solution polymérique chélatrice MEX-CD1 qui sont (i) physiquement séparées par la membrane de dialyse qui (ii) ne peut pas être traversée par le biopolymère greffé en raison de sa taille, assure un potentiel de toxicité très limité pour l'utilisation du dispositif médical MEX-CD1.

La biocompatibilité de la solution polymérique chélatrice MEX-CD1 a été évaluée conformément aux normes ISO 10993-18 (caractérisation chimique) et ISO 10993-4 (hémocompatibilité, hémolyse directe

CONFIDENTIEL

et analyse du temps de céphaline partielle), et des tests de toxicité et de cytotoxicité ont également été effectués (respectivement, séries d'injection intraveineuse directe chez des rats sains avec un niveau d'effet nocif non observé et évaluation de la dose maximale tolérée, et prolifération de lignées cellulaires 13.S.1.24 non différenciées). La caractérisation chimique de la solution polymérique chélatrice MEX-CD1 n'a mis en évidence aucun produit chimique lixiviable en dehors du DOTA-GA résiduel non greffé (<10%) et la quantité de solvant résiduel était bien inférieure aux limites acceptables pour les doses recommandées (solvant résiduel : 1,2-propanediol <1% p/p, acide acétique <5% p/p). Les tests d'hémolyse directe, l'analyse du temps partiel de céphaline ainsi que les tests de toxicité et de cytotoxicité n'ont déclenché aucun effet indésirable pour les doses recommandées en cours d'utilisation. Les essais et la posologie de la charge microbienne (selon l'EP 2.6.12), de la stérilité (selon l'EP 2.6.1 et de l'ISO 17665-1) et des endotoxines (selon l'EP 2.6.14) ont été validés et font partie du contrôle de la libération du dispositif médical MEX-CD1 (charge microbienne selon l'EP 2.6.12, stérilisation et endotoxines revendiquées <0,5 UI/L). Les détails des tests et les résultats sont disponibles dans le BI (Annexe 1).

Le dispositif médical MEX-CD1 n'est pas marqué CE.

Le dispositif médical MEX-CD1 doit être utilisé en association avec :

Composants à usage unique	Composants réutilisables
Deux (2) sacs de 5 L de solution stérile MultiBic (Fresenius Medical Care) - Réf : F00007445)	Moniteur multiFiltratePRO (Fresenius Medical Care – Réf. : M205001)
Seringue stérile de 50 mL (B. Braun – Réf. : 300865)	
Kit MultiFiltratePRO AVpaed HD 400 (stérile) (Fresenius Medical Care – Réf. : F00009082)	
Prolongateur en pvc avec robinet à trois voies (Cair LGL– Réf. : PS3302M)	
Deux (2) sacs effluents 10 L (Fresenius Medical Care – Réf. : 5029011)	

2.1.2 Procédures d'investigation

La mise en place du dispositif médical MEX-CD1 requiert la pose de cathéters permettant une circulation extracorporelle (CEC). Dans le cas de la présente investigation clinique, la pose de deux cathéters veineux périphériques de bon calibre (18G) est nécessaire (un à chaque membre supérieur). Ces cathéters permettent la mise en place de la circulation extracorporelle, assurée par un appareil d'hémodialyse dédié exclusivement à l'IC (multiFiltratePRO, Fresenius Medical Care) et équipé de pompes péristaltiques. Par ailleurs, un circuit standard adapté à l'appareil sera utilisé et couplé à un dialyseur pédiatrique de type AVpaed (Fresenius Medical Care). Une anticoagulation préventive étant requise pour permettre la circulation extracorporelle, nous utilisons de l'héparine non fractionnée (HNF) (HEPARINE CHOAY 5000 UI/1 ml) en perfusion continue (IVSE) avec un schéma adapté au taux de plaquette :

- 10 UI/kg/h chez les patients ayant un taux de plaquettes supérieur à 100 G/L
- 5 UI/kg/h chez les patients ayant un taux compris entre 50 et 100 G/L

Cette anticoagulation est injectée directement sur le circuit pour optimiser son efficacité dans la CEC, et limiter les effets systémiques, mais comporte classiquement un faible risque hémorragique.

Afin d'anticiper le risque d'épuration indésirables d'autres métaux par le MEX-CD1, les patients recevront après chaque séance d'épuration (une fois que les différents prélèvements auront été réalisés) une ampoule de mélange d'oligoéléments (spécialité JUNIMIN, ampoule 10 mL injectable). Chaque ampoule contient :

- 6970 µg de gluconate de zinc
- 1428 µg de gluconate de cuivre
- 40,52 µg de gluconate de manganèse
- 13,08 µg de iodure de potassium
- 43,81 µg de sélénite de sodium

CONFIDENTIEL

2.1.3 Procédures additionnelles

Les cathéters veineux périphériques mis en place au niveau des membres supérieurs pour assurer la CEC sont utilisés pour réaliser les prélèvements sanguins veineux pour les bilans biologiques prévus (fer, CST, ferritine, CRP, ionogramme sanguin et NFS) en début et fin de chaque séance d'Epuration ExtraCorporelle (2 x 13 mL par séance soit un total de 78 mL de sang total pour un cycle complet de trois séances S_1, S_2 et S_3).

Temps		Nombre de tubes / patient	Quantité total / patient
Séances d'Epuration ExtraCorporelle (S_1)	H_0¶	1 tube PST (4 mL)	26 mL
		1 tube SST (5 mL)	
		1 tube EDTA (4 mL)	
	H_f*	1 tube PST (4 mL)	
		1 tube SST (5 mL)	
		1 tube EDTA (4 mL)	
Séances d'Epuration ExtraCorporelle (S_2)	H_0¶	1 tube PST (4 mL)	26 mL
		1 tube SST (5 mL)	
		1 tube EDTA (4 mL)	
	H_f*	1 tube PST (4 mL)	
		1 tube SST (5 mL)	
		1 tube EDTA (4 mL)	
Séances d'Epuration ExtraCorporelle (S_3)	H_0¶	1 tube PST (4 mL)	26 mL
		1 tube SST (5 mL)	
		1 tube EDTA (4 mL)	
	H_f*	1 tube PST (4 mL)	
		1 tube SST (5 mL)	
		1 tube EDTA (4 mL)	

¶ ± 10 min. du début de la séance

* jusqu'à 10 min après la fin de la séance d'Epuration ExtraCorporelle

Les visites de suivi prévues par le protocole (V_1, V_2 et V_3) nécessitent également la réalisation d'un prélèvement sanguin veineux de 13 mL à chaque visite (soit un total de 39 mL de sang total pour les trois visites).

Temps	Nombre de tubes / patient	Quantité total / patient
Visite de suivi (V_1)<br>S_3+7 jours [± *2 jours*]	1 tube PST (4 mL)	13 mL
	1 tube SST (5 mL)	
	1 tube EDTA (4 mL)	
Visite de suivi (V_2)<br>S_3+28 jours [± *2 jours*]	1 tube PST (4 mL)	13 mL
	1 tube SST (5 mL)	
	1 tube EDTA (4 mL)	
Visite de fin d'étude (V_3)<br>S_3+90 jours [± *7 jours*]	1 tube PST (4 mL)	13 mL
	1 tube SST (5 mL)	
	1 tube EDTA (4 mL)	

Etude principale et ancillaire No. 1

L'un des objectifs secondaires de l'étude est d'évaluer l'effet du dispositif médical MEX-CD1 sur les concentrations en éléments de trace métallique : fer (étude principale) et cuivre, zinc, sélénium, cadmium, plomb (étude ancillaire) dans le sang et liquide de dialyse.

Tous les prélèvements réalisés dans le cadre de l'étude sont adressés au Laboratoire d'Analyse de Traces et Métaux Toxiques (Dr François PARANT) sur l'Hôpital Lyon Sud.

Les prélèvements du liquide de dialyse (tube à centrifuger stérile) sont réceptionnés, centrifugés et analysés pour déterminer les quantités de fer. Les fonds de tubes sont prélevés, aliquotés dans 1 tube

CONFIDENTIEL

Eppendorf de 1 mL, stockés et congelés à -20°C. Les prélèvements sanguins (tubes d'analyse des oligo-éléments) sont réceptionnés et stockés à -20°C.

Les tubes Eppendorf et tubes d'analyses des oligo-éléments sont récupérés et analysés par l'institut lumière pour établir le profil multi minéral (quantification des oligo-éléments et macro-minéraux autre que le fer) dans le cadre de l'étude ancillaire 1 : Analyse sous la responsabilité du Dr Olivier TILLEMENT. L'analyse des échantillons dans le cadre de l'étude ancillaire 1 sera réalisée ultérieurement (recherche de financements complémentaires).

Les prélèvements sont les suivants :

- Prélèvement sanguin veineux (tube d'analyse des oligo-éléments 6 mL) à différents temps : J_0, S_1H_0, S_1H_f, V_1 et V_2.

Temps		Nombre de tubes / patient	Quantité totale / patient
Inclusion	J_0	1 tube d'analyse des oligo-éléments	6mL
Séances d'Epuration ExtraCorporelle (S_1)	S_1H_0	1 tube d'analyse des oligo-éléments	6 mL
Séances d'Epuration ExtraCorporelle (S_1)	S_1H_f	1 tube d'analyse des oligo-éléments	6 mL
Visite de suivi (V1)<br>S_3+7 jours [± *2 jours*]	V_1	1 tube d'analyse des oligo-éléments	6 mL
Visite de suivi (V2)<br>S_3+28 jours [± *7 jours*]	V_2	1 tube d'analyse des oligo-éléments	6 mL

- Prélèvements du liquide de dialyse au niveau de la ligne d'effluent (1 tube à centrifuger stérile 15mL exempt de métaux lourds – Labcon// Dutscher Référence 949892) toutes les heures (H_1, H_2,H_f) de chaque séance d'Epuration ExtraCorporelle, et au niveau du sac d'effluent (2 tubes à centrifuger stériles 15mL exempts de métaux lourds – Labcon// Dutscher Référence 949892) en fin de séance (H_f). L'un des tubes de 15mL prélevé au niveau du sac d'effluent en fin de séance (H_f) de chaque séance (S_1, S_2 et S_3) sera stocké à -20°C. Les 3 tubes de 15 mL collectés et congelés seront récupérés par MexBrain après la 3ème séance d'Epuration ExtraCorporelle (S_3) pour une analyse centralisée.

Temps		Nombre de tubes / patient	Quantité totale/ séance	Echantillonnage
Séances d'Epuration ExtraCorporelle (S_1)	$S_1H_1^{\theta}$	1 tube à centrifuger stérile 15 mL exempt de métaux lourds	75 mL	1 tube Eppendorf (1 mL)
	$S_1H_2^{\theta}$	1 tube à centrifuger stérile 15 mL exempt de métaux lourds		1 tube Eppendorf (1 mL)
	$S_1H_f^{\theta,\ddagger}$	3 tubes à centrifuger stériles 15 mL exempts de métaux		2 tubes Eppendorf (2 x 1mL)
Séances d'Epuration ExtraCorporelle (S_2)	$S_2H_1^{\theta}$	1 tube à centrifuger stérile 15 mL exempt de métaux lourds	75 mL	1 tube Eppendorf (1 mL)
	$S_2H_2^{\theta}$	1 tube à centrifuger stérile 15 mL exempt de métaux lourds		1 tube Eppendorf (1 mL)
	$S_2H_f^{\theta,\ddagger}$	3 tubes à centrifuger stériles 15 mL exempts de métaux		2 tubes Eppendorf (2 x 1mL)
Séances d'Epuration ExtraCorporelle (S_3)	$S_3H_1^{\theta}$	1 tube à centrifuger stérile 15 mL exempt de métaux lourds	75 mL	1 tube Eppendorf (1 mL)
	$S_3H_2^{\theta}$	1 tube à centrifuger stérile 15 mL exempt de métaux lourds		1 tube Eppendorf (1 mL)
	$S_3H_f^{\theta,\ddagger}$	3 tubes à centrifuger stériles 15 mL exempts de métaux		2 tubes Eppendorf (2 x 1mL)

$^{\theta}$ au niveau de la ligne d'effluent (1 tube)

‡ au niveau du sac de l'effluent (2 tubes)

CONFIDENTIEL

- Prélèvements (tube à centrifuger stérile 15mL exempt de métaux lourds – Labcon// Dutscher Référence 949892) au niveau de la poche de dialysat en début (H_0) de chaque séance.

**Temps**		**Nombre de tubes / patient**	**Quantité totale/ séance**	**Echantillonnage**
Séances d'Epuration ExtraCorporelle (S_1)	S_1H_0	1 tube à centrifuger stérile 15 mL exempt de métaux lourds	15 mL	1 tube Eppendorf (1 mL)
Séances d'Epuration ExtraCorporelle (S_2)	S_2H_0	1 tube à centrifuger stérile 15 mL exempt de métaux lourds	15 mL	1 tube Eppendorf (1 mL)
Séances d'Epuration ExtraCorporelle (S_3)	S_3H_0	1 tube à centrifuger stérile 15 mL exempt de métaux lourds	15 mL	1 tube Eppendorf (1 mL)

Etude ancillaire No. 2$^{\Delta}$

Pour estimer l'effet de la déplétion martial (fer) des séances d'Epuration Extracorporelle sur les programmes transcriptomiques des éléments matures circulants (différenciation), une biocollection est mise en place à partir de prélèvements sanguins réalisés à différents temps. La biocollection sera centralisée au sein du Centre de Ressources Biologiques (Hôpital Lyon Sud). L'analyse des échantillons sera réalisée ultérieurement (recherche de financements complémentaires).

**Temps**	**Nombre de tubes / patient**	**Quantité total / patient**	**Echantillonnage**
Inclusion (J_0)	3 tubes EDTA (4 mL)<br><br>1 tube sec (4 mL)	12 mL<br><br>4 mL	2 cryotubes (PBMC)<br>2 cryotubes (Plasma EDTA)<br>2 cryotubes (sérum)
Séances d'Epuration ExtraCorporelle (S_2)	3 tubes EDTA (4 mL)<br><br>1 tube sec (4 mL)	12 mL<br><br>4 mL	2 cryotubes (PBMC)<br>2 cryotubes (Plasma EDTA)<br>2 cryotubes (sérum)
Visite de suivi (V_1)<br>S_3+7 jours [± *2 jours*]	3 tubes EDTA (4 mL)<br><br>1 tube sec (4 mL)	12 mL<br><br>4 mL	2 cryotubes (PBMC)<br>2 cryotubes (Plasma EDTA)<br>2 cryotubes (sérum)
Visite de fin d'étude (V_3)<br>S_3+90 jours [± *7 jours*]	3 tubes EDTA (4 mL)<br><br>1 tube sec (4 mL)	12 mL<br><br>4 mL	2 cryotubes (PBMC)<br>2 cryotubes (Plasma EDTA)<br>2 cryotubes (sérum)

$^{\Delta}$ Sur la totalité des patients inclus et ayant bénéficié d'au moins deux séances d'Epuration ExtraCorporelle.

2.1.4 Bénéfices escomptés du DM

Les chélateurs aujourd'hui utilisés pour le traitement de la surcharge en fer présentent de nombreux effets indésirables potentiellement invalidants (nausées, vomissements, douleurs abdominales) qui peuvent compromettre l'observance thérapeutique et rendre le traitement inefficace. Cette absence d'efficacité ou leur impossibilité d'utilisation s'accompagne alors d'un maintien de la surcharge martiale, d'une forte diminution de la survie post-greffe et de conséquences graves sur le fonctionnement d'organes qui, selon la pathologie sous-jacente, peut altérer le pronostic des patients.

Le MEX-CD1 (dispositif médical – Classe III) est un composé polymérique hyper chélatant utilisé en combinaison avec une technique d'hémodialyse à faible volume pour extraire du sang le fer libre en excès. Ce procédé pourrait donc permettre l'Epuration ExtraCorporelle d'une quantité importante de fer chez des patients en surcharge. Pour vérifier cette hypothèse, nous proposons cet essai clinique pilote, monocentrique multisites chez des patients qui présentent une surcharge en fer post-transfusionnelle nécessitant une stratégie de déplétion en fer, quelle que soit l'hémopathie, l'hémoglobinose ou toute autre pathologie d'origine immuno-hématologique sous-jacente et présentant sont intolérants ou réfractaires aux chélateurs.

En cas d'efficacité sur la surcharge en fer chez ces patients, le dispositif pourrait constituer un nouveau traitement incontournable pour cette population de patients, avec un bénéfice très probable sur la réduction des complications liées à la surcharge martiale et la mortalité. En effet, les résultats

CONFIDENTIEL

spectaculaires apportés par la chélation standard confirment le rôle majeur de la surcharge en fer dans la morbi-mortalité de ces patients.

2.2 Caractéristiques techniques et fonctionnelles du DM évalué

Un nouveau dispositif médical a été développé et breveté conjointement par MexBrain et l'ILM pour permettre l'extraction sélective des métaux du corps humain. Cette thérapie est basée sur l'utilisation d'un dispositif médical de purification du sang, associé à un fluide polymérique contenant un biopolymère ultrachélatant. Cette technologie a dans un premier temps été proposée pour le traitement de patients souffrant d'une surcharge en cuivre (maladie de Wilson) après sa validation sur un modèle de gros animal (étude 'MEXWILS' en cours - ID RCB : 2022-A01628-35 - NCT05917327).

Le principe de la technologie repose sur la combinaison de deux technologies déjà présentes en clinique et permettant de réduire les inconvénients intrinsèques à chacune : la dialyse et la chélation. Dans cette technologie, le composé actif (MEX-CD1) est maintenu séparé du sang par la membrane de dialyse.

Cette séparation innovante permet de combiner plusieurs avantages pour la chélation des métaux :

- L'absence de contact direct entre le produit (MEX-CD1) et la circulation sanguine permet en premier lieu d'éviter toute toxicité qui pourrait être induite par le biopolymère ultrachélatant puisque qu'il n'existe aucun contact entre le produit et les différents composants du sang et du corps humain.
- Une très haute efficacité procurée par la haute concentration en chélateurs greffés sur le biopolymère additivé au dialysat. La séparation des chélateurs du reste du corps humain et particulièrement du système sanguin permet d'augmenter très largement les quantités de polymère utilisées sans risquer d'effet secondaire néfaste pour la santé du patient. Le biopolymère ultrachélatant peut ainsi être introduit en excès dans le dialysat afin de permettre une extraction optimale des métaux ciblés.
- La grande spécificité résultant du choix du chélateur et de la membrane. Le choix du chélateur tout d'abord, permet d'obtenir une spécificité vis-à-vis des métaux ciblés. Ainsi un chélateur a été sélectionné afin d'extraire spécifiquement le fer présent en trop grande quantité dans la circulation des patients sélectionnés. Le choix de la membrane permet ensuite d'obtenir une spécificité vis-à-vis de l'environnement des métaux ciblés. Il est nécessaire, dans ce type de traitement, de ne cibler que les métaux présentant une toxicité. Ainsi un atome de fer lié par la transferrine, essentiel au bon fonctionnement du corps humain, ne pourra être extrait car la membrane empêchera le passage de la transferrine dans le dialysat alors qu'un atome de fer associé à de petites espèces moléculaires, appelé fer catalytique et qui présente une très forte toxicité, pourra être extrait. Cette spécificité permet de ne cibler que les métaux d'intérêt qui sont présents dans une configuration toxique sans impact sur les métaux essentiels au bon fonctionnement de l'organisme.

Cette nouvelle technologie permet donc l'extraction ciblée et spécifique de métaux hautement toxiques dans la circulation sanguine chez des patients affaiblis. Cette extraction permettra un impact fort sur la morbi-mortalité des patients affectés et donc une diminution des pathologies secondaires graves résultantes.

2.3 Risques et bénéfices du DM évalué

2.3.1 Bénéfices

Le principal bénéfice attendu pour les patients qui accepteront de participer à l'étude serait une diminution significative de la charge en fer total de l'organisme, objectivée par une baisse de leur ferritinémie à l'issue du cycle de 3 séances d'Epuration ExtraCorporelle.

2.3.2 Contraintes

Le cycle de 3 séances d'Epuration ExtraCorporelle étant prévu sur une semaine, le patient est contraint de revenir sur le site (service de néphrologie de l'Hôpital Lyon Sud) de façon rapprochée pour la réalisation de ces séances d'une durée réglée de 4 heures chacune.

CONFIDENTIEL

Des visites de suivi sur site sont également prévus à 7 jours [± 2 jours] (V_1) et 28 jours [± 2 jours] (V_2) de la 3ème séance (S_3) d'Epuration ExtraCorporelle.

Enfin, une visite de fin d'étude (V_3) est prévue sur site à 90 jours [± 7 jours] après la dernière séance S_3 d'Epuration ExtraCorporelle.

2.3.3 Risques potentiels

Aucun effet indésirable lié au MEX-CD1 n'a été constaté lors des études précliniques.

En l'absence de contact direct du MEX-CD1 avec le sang, les risques potentiels sont liés à la procédure d'hémodialyse et aux différents prélèvements sanguins veineux répétés.

Manifestations communes et bénignes associées à l'hémodialyse (entre 1 et 10% des séances) :

N.B : le dispositif MEX-CD1 peut être considéré comme un hémodialyse à très bas débit (600 mL/h). Les effets indésirables indiqués sont issus des données concernant l'hémodialyse conventionnelle (utilisant des débits 60 fois supérieurs) et sont dus pour la plupart aux fortes variations ioniques et osmolaires déclenchées par cette forte dialysance. Une partie de ces effets est aussi due au contact du sang avec la membrane, ou avec l'anticoagulation (identiques entre les 2 techniques).

Crampes musculaires

Sans être dangereuses, les crampes musculaires, qui touchent principalement les membres inférieurs peuvent être douloureuses. Elles sont déclenchées par un taux d'ultrafiltration rapide ou par une déplétion extracellulaire excessive. Les crampes cèdent à l'administration de solutions hypertoniques (sodium 10%, glucose 50%) et à l'arrêt de l'ultrafiltration.

Frissons, inconfort

Nausées, vomissements

Les nausées et les vomissements survenant en cours de dialyse sont habituellement prémonitoires ou contemporains d'une chute de pression artérielle. Plus rarement, ces troubles digestifs s'accompagnent d'hypertension artérielle, de céphalées, ou d'autres manifestations neurologiques centrales (obnubilation, troubles visuels) et font évoquer un syndrome de déséquilibre, ou une hypercalcémie (ou un syndrome de l'eau dure en cas d'épidémie dans un centre) ou tout autre désordre osmotique.

Syndrome de déséquilibre

Ce syndrome est caractérisé par l'apparition de céphalées accompagnées parfois de photophobie et de nausées.

Fatigue post-dialytique

Certains patients peuvent se plaindre d'une asthénie persistante plusieurs heures après la fin de la dialyse. Cette fatigue s'observe plus volontiers en présence d'ultrafiltration importante et d'hypovolémie postdialytique.

Céphalées

Les céphalées de fin de séance sont relativement fréquentes au cours des premières séances de dialyse ou au cours des séances courtes à très haute efficacité. Apparues en cours ou en fin de séance de dialyse, elles sont parfois contemporaines d'une hypertension artérielle et de troubles digestifs. Elles doivent faire suspecter une hypercalcémie, une alcalose métabolique sévère ou un syndrome de déséquilibre.

Hémorragies – hématomes au niveau du point de ponction

Très peu probable dans le cas présent, au vu des abords périphériques temporaires choisis pour l'investigation clinique. Les événements hémorragiques restent rares chez les patients dialysés, et sont en général limités à des hématomes superficiels ou des hémorragies digestives rarement sévères. Cependant, ils seront surveillés spécifiquement dans la population de l'étude.

Manifestations rares et graves survenant pendant la séance de dialyse (<1% des séances) :

Troubles du rythme cardiaque

Des extrasystoles variées ou des accès de fibrillation, peuvent être observées, en particulier chez les sujets porteurs de cardiopathie. Le risque de fibrillation auriculaire est accru par l'hypovolémie

CONFIDENTIEL

notamment chez les coronariens et les patients digitalisés. Des flux trop rapide potassiques et calciques perdialytiques sont parfois en cause dans ces arythmies.

Angor - Infarctus du myocarde

Une crise d'angor ou un infarctus du myocarde peuvent être déclenchés par une séance d'hémodialyse, notamment à l'occasion d'un épisode d'hypotension artérielle sévère chez un patient à risques.

Convulsions

La survenue d'une crise comitiale en dialyse doit faire évoquer en premier lieu une hypotension artérielle sévère chez un sujet à risques. Dans ce cas, la correction de l'hypovolémie suffit à remédier au problème.

Réactions fébriles et chocs pyrogènes

Les réactions fébriles en cours de séance de dialyse sont rares. Lorsqu'elles surviennent précocement après le branchement, elles sont la conséquence du contact du sang avec des substances pyrogènes (endotoxines ou autres) présentes dans le circuit sanguin.

Manifestations de type allergique

Gêne respiratoire à composante bronchospasmodique, toux quinteuse, écoulement nasal, conjonctivite, impression de chaleur généralisée, érythème cutané prurigineux avec parfois chute de la tension artérielle, réaction anaphylactique ou anaphylactoïde

Hémolyse intravasculaire

Incidents et accidents survenant pendant la période interdialytique (extrêmement rares) :

Infections

Le risque infectieux est nettement plus élevé chez les urémiques que celui des sujets normaux. Cette sensibilité à l'infection tient d'une part à la technique de dialyse elle-même, qui met le sang du malade en contact avec le milieu extérieur de façon répétitive au long cours, d'autre part à la persistance de troubles de l'immunité cellulaire. Les complications infectieuses, bactériennes ou virales, peuvent survenir à tout moment dans la vie du dialysé. Les précautions d'asepsie et le respect strict des règles universelles d'hygiène, l'isolement de patients porteurs de virus de l'hépatite B, sont des facteurs essentiels dans la prévention de ces complications.

Colite ischémique

La survenue de complications digestives de type ischémique est devenue très rare depuis l'utilisation de tampon bicarbonate. Le risque persiste néanmoins chez les patients vasculaires. Habituellement, l'ischémie de la muqueuse colique ou grêle est déclenchée par un épisode d'hypotension artérielle sévère survenue au cours d'une séance de dialyse.

Au vu du bénéfice potentiel pour les patients et du faible risque encouru limité et contrôlé, notamment, par l'utilisation de cathéters périphériques et d'une faible dose d'anti coagulation, la balance bénéfice/risque paraît favorable au patient.

3 JUSTIFICATION SCIENTIFIQUE

La surcharge en fer post-transfusionnelle est une complication majeure des transfusions répétées nécessaires au cours de nombreuses hémopathies, hémoglobinopathies et autres pathologies d'origine immuno-hématologique:

- Les hémopathies concernées sont les syndromes myélodysplasiques, myélofibrose (primitive ou secondaire à un autre syndrome myéloprolifératif) ou les formes frontières myélodysplasiques/myéloproliférative (incluant les leucémie myélomonocytaire chronique). Les patients atteints de leucémie aigue (myéloïde ou lymphoïde) en rémission complète ayant achevés les consolidations dès suite d'un traitement par chimiothérapie intensive (et avant initiation d'une maintenance) ou en rémission complète mais en attente d'allogreffe de cellules souches hématopoïétiques.
- Les hémoglobinoses concernées sont les thalassémies, les drépanocytoses et les autres anémies d'origines congénitales liées à un déficit enzymatique/protéiques (i.e. anémie sidéroblastiques

CONFIDENTIEL

congénitales ALAS2 ou apparentées) ou à un défaut de structure membranaire (i.e. sphérocytose, stomatocytose).

- Les pathologies d'origine immuno-hématologiques concernés peuvent être d'origine acquise (aplasie médullaire idiopathique/hémoglobinurie paroxytisque nocturne (HPN), érythroblastopénies acquises) ou constitutionelle (i.e. anémies dysérythropoïétiques congénitales (CDA), anémie de Diamond-Blackfan). Chez les patients suivis pour une HPN, un traitement par bloqueur de la voie du complément est autorisé tant que le patient est sous le même traitement depuis au moins 3 mois.

Avec l'augmentation des réserves en fer, la transferrine plasmatique est rapidement saturée ce qui aboutit à la présence de fer libre dans la circulation [1] qui peut traverser la membrane cellulaire dans certains organes et agir comme catalyseur pour former des espèces réactives de l'oxygène et provoquer des dommages cellulaires [2, 3].

Les patients atteints de syndromes myélodysplasiques (SMD) présentent fréquemment une anémie, qui nécessite des transfusions régulières. Les patients atteints de SMD dépendants des transfusions présentent également une érythropoïèse inefficace, ce qui peut perturber l'équilibre en fer et entraîner une surcharge en fer.

Des mécanismes similaires sont également observés dans d'autres hémopathies chroniques, certaines hémoglobinopathies et d'autres pathologies d'origine immuno-hématologique nécessitant des transfusions répétées.

La surcharge tissulaire en fer est détectable dans les mois qui suivent le début des transfusions et est responsable d'une nette surmortalité liée aux atteintes d'organes [4], notamment cardiaque et hépatique. La moelle est également touchée et la surcharge en fer aggrave la dysmélopoïèse et le risque de transformation des myélodysplasies [5, 6], et influe sur le pronostic des greffes de cellules souches.

En dehors du syndrome myélodysplasique, d'autres hémopathies myéloïdes telles que la myélofibrose primitive ou secondaire à un syndome myéloprolifératif sont potentiellement associées à des besoins transfusionnels compliqués d'hémochromatose secondaire ainsi que des patients en rémission en attente d'allogreffe dès suite de traitements intensifs pour une leucémie aigue. Dans la myélofibrose, l'anémie prononcée et l'érythropoïèse inefficace, associées à une splénomégalie myéloïde, majorent la dépendance aux concentrés globulaires, conduisant à une accumulation rapide de fer tissulaire et hépatique délétère pour le pronostic global [ref].

Chez les patients en rémission en attente d'allogreffe dès suite de traitements intensifs pour une leucémie aigue, le titre de ferritine pré-transplantation conditionne fortement la probabilité de survie post allogreffe. Dans ce contexte, l'hémochromatose est secondaire aux transfusions intératives pendant les phases de traitement par chimiothérapie intensive plus que par une problématie d'érythropoïèse inéficace. Une large méta-analyse portant sur 25 études différentes a rapporté qu'une ferritine pré-allogreffe élevée était fortement associée à une moins bonne survie globale (HR = 1,82 ; IC95% : 1,47-2,26 ; $p < 0{,}001$), à une mortalité non liée à la rechute plus élevée (HR = 2,28 ; IC95% : 1,79-2,89 ; $p < 0{,}001$) et à une survie sans progression réduite (HR = 1,72 ; IC95% : 1,27-2,33 ; $p < 0{,}001$) [ref].

Bien qu'il soit admis que le taux de ferritine pré allogreffe soit déterminant de la survie post allogreffe, il n'est en général pas possible de réaliser une chélation martiale efficace chez ces patients [14]. En effet, les traitements chélateurs actuels (oraux ou parentéraux) mettent plusieurs mois à agir. Cependant, chez les patients atteints de leucémie aigue, en cas de rémission, l'allogreffe doit être réalisé au plus vite afin de réduire le risque de rechute.

D'autres situations d'origine non néoplasique tels que les hémoglobinoses d'origine constitutionelles (i.e. drépanocytose, thalassémie, autres anémies hémolytiques lié à un déficit enzymatique ou à un défaut de structure membranaire) ou les syndrome de défaillances médullaire (i.e. aplasie médullaire idiopathique) affectant principalement des sujets jeunes, peuvent être associé à des problématiques d'hémochromatose secondaire aux transfusions répétées. Chez les patients transfusés au long cours pour une thalassémie majeure par exemple, la surcharge en fer est d'environ 2-12g par an [7]. Chez ces patients atteints d'hémoglobinoses chroniques, l'exposition prolongée à la surcharge martiale post-transfusionnelle induit une morbi-mortalité cardiovasculaire et endocrinienne majeure à long terme [ref, ref]. Des études longitudinales ont démontré qu'un taux de ferritine élevé de manière persistante ou un

CONFIDENTIEL

pic de surcharge au cours de la vie augmentent significativement le risque d'insuffisance cardiaque, de diabète sucré et de surmortalité globale [ref, ref]. Par conséquent, un suivi régulier par imagerie par résonance magnétique (IRM) et une adaptation rigoureuse de la chélation demeurent indispensables pour infléchir ce pronostic péjoratif.

Les complications liées à la surcharge en fer, telles que l'insuffisance cardiaque ou hépatique, les endocrinopathies et le risque accru d'infection, peuvent contribuer à réduire la survie des patients atteints de pathologies induisant une hémochromatose secondaire. Le but du traitement chélateur est donc de prévenir les complications à long terme cardiaques, hépatiques et endocriniennes.

Il n'y a actuellement peu de consensus sur le choix du chélateur optimal à recommander, toute indication confondue. L'âge du patient, la présence ou l'absence de comorbidités, les effets secondaires des traitements disponibles et les préférences du patient sont à considérer au moment de l'instauration d'un traitement par chélation. Aux Etats-Unis et en Europe, le deferasirox et la deferiprone sont aujourd'hui les chélateurs les plus utilisés, disponibles par voie orale [8].

Le deferasirox à 40 mg/kg permet une excrétion nette du fer de 0,445 mg/kg par jour. L'objectif du traitement est d'obtenir un taux de ferritine <1000 µg/L[9].

Une intensification du traitement peut être proposée, notamment lorsqu'il existe une insuffisance cardiaque en rapport avec la surcharge en fer, avec la deferiprone seule ou associée au deferasirox [10-12]. Il existe de très nombreux schémas d'association de chélateurs en traitements combinés ou séquentiels [10].

Cependant, les chélateurs présentent des effets indésirables potentiellement invalidants (nausées, vomissements, douleurs abdominales) qui peuvent compromettre l'observance thérapeutique et rendre le traitement inefficace [13].Ils peuvent également s'avérer insuffisamment efficaces chez certains patients malgré une utilisation aux doses optimales.

Ainsi, les données tirées de plusieurs études rétrospectives tendent à montrer que la surcharge en fer peut avoir des conséquences cliniques pour les patients, quelle que soit la pathologie sous-jacente, notamment le dysfonctionnement d'un organe, et une réduction de la survie globale [15-18]. La surcharge en fer a donc un impact important sur la morbi- mortablité et la prise en charge des patients polytransfusés, et ce, malgré l'avènement des traitements chélateurs [13] d'où l'intérêt de trouver d'autre modalités pour diminuer cette surcharge en fer.

Le MEX-CD1 (dispositif médical – Classe III) est un composé polymérique hyper chélatant utilisé en combinaison avec une technique d'hémodialyse à faible volume pour extraire du sang le fer libre en excès. Ce procédé pourrait donc permettre l'épuration extracorporelle d'une quantité importante de fer chez des patients en surcharge.

CONFIDENTIEL

4 OBJECTIFS ET CRITERES DE JUGEMENT

Objectifs	Critères de jugement	Analyse statistique
Principal :		
Estimer l'effet du MEX-CD1 dans la chélation du fer à la fin de chacune des trois séance d'un cycle d'Epuration ExtraCorporelle.	Quantité de fer extrait mesurée au niveau du sac d'effluent (H_4) à la fin de chaque séance d'Epuration ExtraCorporelle (S_1, S_2, S_3) selon la formule :<br>• quantité totale (µg) de fer extrait : [fer (µg/L) du sac d'effluent (H_f) - fer (µg/L) de la poche de dialysat (H_0)] x volume d'effluent (L) = quantité extraite (µg)	• Estimation aux trois séances d'Epuration ExtraCorporelle de la quantité de fer chélaté à partir des valeurs numériques décrites selon leur moyenne, écart-type, médiane, valeur maximale et minimale.
Secondaires :		
1) Décrire la cinétique de la chélation du fer au cours de chacune des trois séance d'un cycle d'Epuration ExtraCorporelle.	• Quantité de fer mesurée sur la ligne d'effluent (H_1, H_2, H_f) au cours de chaque séance d'Epuration ExtraCorporelle (S_1, S_2, S_3) selon la formule :<br>• cinétique d'extraction du fer : [fer (µg/L) sur la ligne effluent (H_1, H_2, H_f)- fer (µg/L) de la poche de dialysat (H_0)] x QD (L/h) = fer extrait (µg/h)	• Représentation graphique de la cinétique des quantités de fer chélaté sur la ligne d'effluent à partir des valeurs numériques décrites selon leur moyenne, écart-type, médiane, valeur maximale et minimale.
2) Estimer l'effet de l'Epuration ExtraCorporelle du MEX-CD1 sur les paramètres du bilan martial à court terme.	• Variations de fer, ferritine, CST mesurées entre le début (H_0) et la fin (H_f) de chaque séance d'Epuration ExtraCorporelle (S_1, S_2, S_3) ;<br>• Variations de fer, ferritine, CST mesurées entre le début de la 1ère séance (S_1H_0) et la fin de la 3ème séance (S_3H_f) ;<br>• Variations de fer, ferritine,CST mesurées entre la fin de la dernière séance S_1H_f et V_1 (S_3 +J_7) / V_2 (S_3 +J_{28}) / V_3 (S_3 +J_{90})	• Les variations des valeurs de biomarqueurs (fer, ferritine, CST) seront analysées en différence absolue ainsi qu'en pourcentage de variation. Un modèle à effet mixte sera réalisé avec un intercept aléatoire pour estimer la moyenne en prenant en compte les données répétées. Un second modèle sera réalisé en ajoutant l'effet séance en effet fixe.<br>• Quand nécessaire, modélisation des paramètres du bilan martial avec un modèle linéaire à effets mixtes afin de prendre en compte un effet patient et période. Il inclura, en effet fixe, le temps (date de la visite). Le

CONFIDENTIEL

		modèle inclura en effet aléatoire un intercept par patient, et une pente aléatoire.
3) Décrire la tolérance des séances d'Epuration ExtraCorporelle par le MEX-CD1.	Occurrence des évènements indésirables de tout type et notamment les Evènements d'Intérêt Particulier (EIP) de type : hypotension *per* procédure, réaction anaphylactoïde, effets indésirables au point de ponction veineux, troubles ioniques post-séance (sodium, potassium, calcium, magnésium). Pour chaque EIP, l'investigateur déterminera son imputabilité liée à la procédure (cf 11.1.4) et son intensité (cf 11.2.1) selon la CTCAE v5.0.	Les évènements indésirables selon la CTCAE seront analysés en nombre total, en % d'évènements, ainsi qu'en patients ayant eu au moins 1 évènement.<br>Les évènements imputables seront décrits spécifiquement. Une description par grade d'intensité, par SOC et PT, sera effectuée.
4) Évaluer l'effet de l'Epuration ExtraCorporelle avec le MEX-CD1 sur les besoins transfusionnels chez les patients présentant une dépendance transfusionnelle en CGR (applicable uniquement aux patients atteints de syndrome myélodysplasique, myélofibrose, hémoglobinose ou aplasie médullaire/BMF).	Chez les patients présentant une dépendance transfusionnelle en CGR :<br>• Date des transfusions de CGR<br>• Nombre de CGR transfusés par patient	Le nombre de CGR sera décrit par la moyenne, écart-type, médiane, valeur maximale et minimale ou en répartition selon la distribution du nombre de transfusions.
5) Décrire la faisabilité du traitement expérimental.	• Patients ayant réalisé la première séance d'Epuration ExtraCorporelle dans son entièreté.<br>• Patients ayant réalisé un cycle complet de 3 séances d'Epuration ExtraCorporelle consécutives.<br>• Ratio temps réel (heure) sous traitement expérimental / temps programmé (3h20).<br>• Volume total (litre) de sang traité (donnée fournie par l'équipement de dialyse).	Chaque critère de jugement secondaire qualitatif sera décrit par l'effectif et la proportion.. Chaque critère de jugement secondaire quantitatif sera décrit par la moyenne, écart-type, médiane, valeur maximale et minimale.
6) Décrire la tolérance globale de la séance ressentie par le patient.	Cotation par EVA (Echelle Visuelle Analogique) sur la tolérance de la séance (S_1, S_2 et S_3) :<br>• Asthénie/malaise ressenti :<br>[0 : normal – 10 : très fatigué ou malaise intense après chaque séance]<br>• Douleur ressentie :<br>[0 : pas de douleur – 10 : douleur très forte après chaque séance]	Les scores seront décrits selon leur moyenne, écart-type, médiane, valeur maximal et minimale à chaque séance. L'évolution du score entre les séances sera représentée graphiquement pour chaque patient.

CONFIDENTIEL

7) Décrire l'évolution de la qualité de vie ressentie par le patient distance du cycle de séances d'Epuration ExtraCorporelle.	Score à l'auto-questionnaire MOS SF-12 (Annexe 2) à l'inclusion, V_2 et V_3.	Les scores seront décrits selon leur moyenne, écart-type, médiane, valeur maximale et minimale à chaque visite. L'évolution du score entre les visites sera représentée graphiquement pour chaque patient
Etude ancillaire No. 1 :		
Estimer la capacité de chélation du cuivre, zinc, sélénium, cadmium, plomb sanguins d'un cycle de 3 séances d'Epuration ExtraCorporelle avec le MEX-CD1.	Quantités mesurées de cuivre, zinc, sélénium, cadmium et plomb à partir de prélèvements de sang veineux (tubes secs) à J_0 (inclusion), S_1H_0, S_1H_f, V_1 et V_2 ;<br>• à partir de la poche de dialysat avant le début de la séance (H_0), après ajout du MEX-CD1 et homogénéisation, pour déterminer la quantité de métaux initialement présente dans le dialysat;<br>• A partir de prélèvements (1 par heure à H_1, H_2, H_f) au niveau de la ligne effluent pour calculer la cinétique d'extraction des métaux;<br>• à partir du sac d'effluent à la fin de la séance (H_f), après homogénéisation pour déterminer la quantité total (µg) de métaux extrait.	Représentations graphiques des taux mesurés.<br>• cinétique d'extraction des métaux : [métaux (µg/L) sur la ligne effluent - métaux (µg/L) de la poche de dialysat] x QD (L/h) = métaux extrait (µg/h)<br>• quantité total (µg) de métaux extrait : [métaux (µg/L) du sac d'effluent - métaux (µg/L) de la poche de dialysat] x volume d'effluent (L) = quantité extraite (µg)
Etude ancillaire No. 2$^{\Delta}$:		
Estimer l'effet de la déplétion martial (fer) sur les programmes transcriptomiques des éléments matures circulants (différentiation).	Mise en place d'une biocollection d'échantillons PBMC et plasma EDTA à partir de prélèvements sanguins réalisés à : inclusion, S_2, V_1 et V_3.	L'analyse des échantillons sera réalisé dans un second temps (recherche de financements complémentaires).

$^{\Delta}$ Sur la totalité des patients inclus et ayant bénéficié d'au moins deux séances d'Epuration ExtraCorporelle.

CONFIDENTIEL

5 CONCEPTION DE L'INVESTIGATION CLINIQUE

5.1 Information sur le type d'investigation clinique

L'étude MEXIRON est une investigation clinique (IC) de catégorie 2, répondant à la définition de l'article 62.1 du Règlement UE 2017/745 des IC conduites pour établir la conformité du DM de classe III. Il s'agit d'une étude prospective, monocentrique multisites HCL, visant à démontrer la preuve de concept de l'utilisation du dispositif d'épuration extracorporelle MEX-CD1 chez des patients présentent une surcharge en fer post-transfusionnelle nécessitant une stratégie de déplétion et intolérants ou réfractaires aux traitements chélateurs.

L'IC MEXIRON est une étude de pilote non comparative, ouverte, sur une cohorte de 10 patients traités séquentiellement.

5.2 Information sur le dispositif médical

Le MEX-CD1 (dispositif médical – Classe III) est une suspension polymérique hyper-chélatante utilisée en combinaison avec une technique d'hémodialyse à faible volume pour extraire du sang le fer en excès (cf 2.1.1). Le MEX-CD1 a été conçu à partir d'un polysaccharide naturel, le chitosane, en y associant un chélate déjà utilisé en clinique le DOTAGA. Ce polymère fonctionnalisé présente l'avantage d'être biocompatible et fortement chélatant. Il n'existe actuellement pas de dispositif alternatif sur le marché pour l'épuration extracorporelle de métaux lourds.

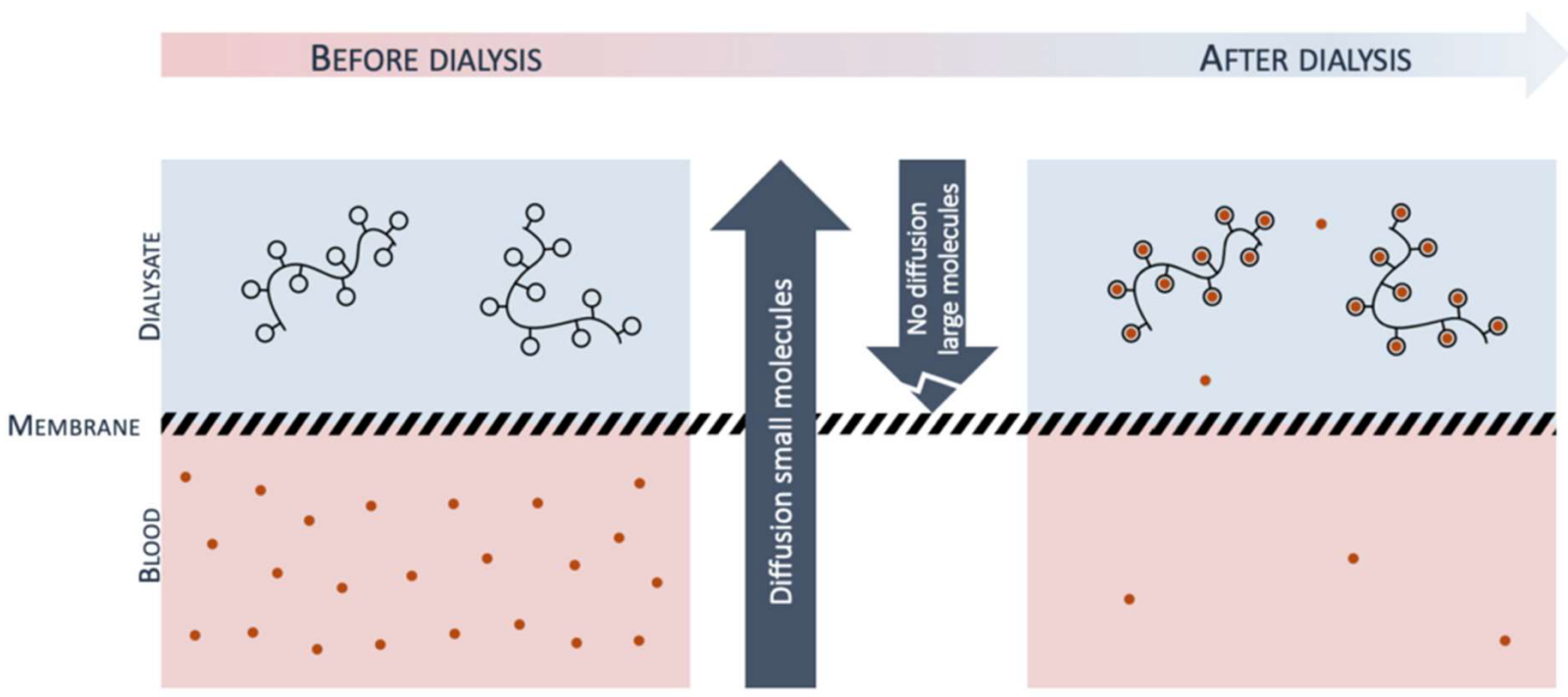

*Figure **1** Principe du MEX-CD1*

L'utilisation du MEX-CD1 nécessite un dispositif d'hémodialyse continue composé d'un dialyseur (membrane), d'un moniteur de dialyse et d'un liquide de dialyse.

- Dialyseur utilisé : AVpaed (Fresenius Medical Care). Ce dialyseur a une zone de membrane effective de 0,2 m², il est stérilisé par rayonnement gamma et stocké entre 4°C et 30°C.
- Moniteur de dialyse : multiFiltratePRO (Fresenius Medical Care). Le système multiFiltratePRO est un système d'épuration extrarénale (EER) continue conçu pour les patients souffrant d'insuffisance rénale aigue. Le système multiFiltratePRO peut dispenser toutes les modalités d'EER.
- La solution MultiBic K+ 0 mmol/L (Fresenius Medical Care) est une solution pour l'hémofiltration et l'hémodialyse, utilisée dans le traitement de l'insuffisance rénale aiguë. Elle est constituée d'une poche à deux compartiments. Les solutions des deux compartiments sont mélangées pour former la solution reconstituée. Sa formule ionique est la suivante : Calcium (Ca^{2+}) 1,75 mmol/l, Magnésium (Mg^{2+}) 0,5 mmol/l, Sodium (Na^{+}) 140 mmol/l, Chlorure (Cl^{-}) 109,5 mmol/l, Lactate 3 mmol/l, Bicarbonate (HCO_3^{-}) 32 mmol/l. Solution reconstituée limpide et transparente. Osmolarité théorique : 287 mOsm/l.

5.3 Mesures pour réduire les biais

Le suivi de tous les patients inclus est strictement identique. Le recueil de données est standardisé au moyen d'un cahier d'observation électronique (eCRF).

CONFIDENTIEL

La réalisation de séances d'Epuration ExtraCorporelle de manière rapprochée, sur une seule semaine, ainsi qu'une période de suivi limitée pour chaque patient inclus (90 jours +/- 7jours maximum après la dernière séance S_3), permettent de limiter le biais d'attrition et le risque de sujets perdus de vue.

Toutefois, tout sujet qui sortirait prématurément de l'IC et qui ne bénéficierait pas d'un cycle complet de 3 séances d'Epuration ExtraCorporelle consécutives, est remplacé.

Un monitoring des données recueillies sera réalisé par un ARC mandaté par le Promoteur conformément au plan de monitoring défini avant le début des inclusions.

5.4 Informations sur les participants

5.4.1 Critères d'inclusion

- Patient âgé de 18 ans et plus.

Ferritinémie >1000 µg/L OU concentration hépatique en fer ≥7 mg/g OU T2* cardiaque <20 ms à l'inclusion, secondaire à des supports transfusionnels dans le cadre d'une hémopathie, une hémoglobinose ou toute autre pathologie d'origine immuno-hématologique. Les hémopathies concernées sont les syndromes myélodysplasiques, myélofibrose (primitive ou secondaire à un autre syndrome myéloprolifératif) ou les formes frontières myélodysplasiques/myéloproliférative (incluant les leucémie myélomonocytaire chronique). Les patients atteints de leucémie aigue (myéloïde ou lymphoïde) en rémission complète ayant achevés les consolidations dès suite d'un traitement par chimiothérapie intensive (et avant initiation d'une maintenance) ou en rémission complète mais en attente d'allogreffe de cellules souches hématopoïétiques. Les hémoglobinoses concernées sont les thalassémies, les drépanocytoses et les autres anémies d'origines congénitales liées à un déficit enzymatique/protéiques (i.e. anémie sidéroblastiques congénitales ALAS2 ou apparentées) ou à un défaut de structure membranaire (i.e. sphérocytose, stomatocytose). Les pathologies d'origine immuno-hématologiques concernés peuvent être d'origine acquise (aplasie médullaire idiopathique/hémoglobinurie paroxytisque nocturne (HPN), érythroblastopénies acquises) ou constitutionelle (i.e. anémies dysérythropoïétiques congénitales (CDA), anémie de Diamond-Blackfan). Chez les patients suivis pour une HPN, un traitement par bloqueur de la voie du complément est autorisé tant que le patient est sous le même traitement depuis au moins 3 mois.

- Patient présentant un taux de plaquettes ≥50 giga/L à l'inclusion.
- Patient présentant une intolérance ou une contre-indication à un traitement chélateur oral / sous cutané ou une inefficacité réelle ou attendue d'un traitement chélateur aux doses optimales. L'inefficacité réelle à un traitement chélateur martial (deferoxamine, deferasirox, défériprone) est définie par l'absence de baisse ou une augmentation continue de la ferritinémie après 6 mois de traitement bien conduit, ou incapacité à maintenir le taux cible (< 1000 µg/L). L'inefficacité attendue ne s'applique qu'aux patients avec projet d'allogreffe en rémission avec un délai entre la fin de leur dernière cure de chimiothérapie et la date théorique d'allogreffe d'au moins 4 semaines. Aucun traitement actif pour l'hémopathie dans cet intervalle n'est réalisable.
- Patient capable de comprendre (francophone) et de se conformer au protocole, ayant signé le consentement éclairé.
- Patient affilié à un régime de sécurité sociale ou bénéficiaire d'un régime similaire.

5.4.2 Critères de non-inclusion

- Patient présentant une hémochromatose primitive (coefficient de saturation de la transferrine CS-Tf > 45%).
- Patient présentant une hyperferritinémie >1000 µg/L dans le cadre d'un syndrome métabolique, d'une pathologie inflammatoire chronique ou tout autre pathologie ne rentrant pas dans le cadre d'une hémopathie, une hémoglobinose ou toute autre pathologie d'origine immuno-hématologique.
- Patient présentant une contre-indication liée à l'utilisation du MEX-CD1 : poids < 50 kg, carence en fer.
- Patient présentant une allergie connue ou contre-indication à l'héparine ou au citrate.
- Patient sous azacitidine (monothérapie ou en association) ou tout autre chimiothérapie (ou considéré comme tel) administré en schéma répété/mensuels pour une leucémie aigüe, syndrome

CONFIDENTIEL

myélodysplasique ou une myélofibrose. Les patients sous inhibiteurs de JAK1/2 peuvent être inclus sous réserve que le traitement ait été introduit depuis au moins 3 mois et que la dose n'ait pas été modifié dans les 4 dernières semaines avant l'inclusion.
- Patient ayant reçu un traitement par luspatercept ou EPO dans le mois précédant l'inclusion
- Patient avec indication pour un traitement par allogreffe de cellules souches dans les 4 semaines après son inclusion dans l'étude.
- Patient présentant une allergie connue aux crustacés (MEX-CD1 contenant du chitosan d'origine animale) ou à l'un des autres composant du MEX-CD1.
- Patients présentant un abord vasculaire périphérique difficile ou à préserver (notamment en cas de projet de dialyse).
- Patient ayant une infection connue telle que la tuberculose, au virus d'immunodéficience humaine (VIH) ou maladie liée au syndrome d'immunodéficience acquise (SIDA).
- Patient ayant un test positif pour l'antigène de surface de l'hépatite B et/ou l'ARN de l'hépatite C (si sérologie positive)
- Patient participant à une autre recherche interventionnelle qui pourrait interférer avec les résultats de l'étude.
- Patient majeur sous mesure de protection légale (tutelle, curatelle) ou hors d'état d'exprimer leur consentement.
- Patient faisant l'objet de soins psychiatriques.
- Patient privé de liberté par une décision judiciaire ou administrative.
- Femme enceinte (Test de grossesse adéquat par dosage de la gonadotrophine chorionique humaine, hCG pour les femmes en âge de procréer) ou allaitante.

5.4.3 Critères de sortie prématurée

- Survenue d'un événement indésirable de type de réaction anaphylactique ou anaphylactoïde pendant une des séances.
- Abord vasculaire difficile observé et contre-indiquant la réalisation de la séance d'Epuration ExtraCorporelle (selon jugement de l'investigateur et avis spécialisé).
- Retrait de consentement du patient.

5.4.4 Taille de la population et modalités de recrutement

L'IC MEXIRON est une étude pilote visant à estimer la tolérance et la faisabilité logistique de l'intervention proposée et obtenir des premières données cliniques et biologiques qui seraient nécessaires pour calculer la taille d'échantillon d'une future étude de plus grande envergure.

Nous prévoyons un nombre total de 10 sujets qui a suivi un cycle complet de 3 séances d'Epuration ExtraCorporelle. Pour pallier d'éventuelles sorties prématurées (patient qui n'auraient pas bénéficié d'un cycle complet de 3 séances), nous prévoyons d'inclure un total de 13 patients.

La durée d'inclusion prévue (12 mois) semble être suffisante au regard du nombre de patients, répondant aux critères d'éligibilité définis par le protocole, et pris en charge au sein du centre.

La file active actuelle de syndrome myélodysplasique est de 450 patients dont une cinquantaine est actuellement en support transfusionnel. Le nombre de diagnostics annuels de syndrome myélodysplasique est de 50 par an sur notre centre. Pour les patients présentant une myélofibrose primitive (ou secondaire à un précédent syndrome myéloprolifératif), la file active du centre est de 100 patients dont 30-35 ont un support transfusionnel actif. Chez les patients allogreffés pour une leucémie aigue myéloïde, la file active annuelle est de 70/an sachant et que 40% d'entre eux présenteront une surcharge martiale pré-allogreffe. La file active des patients atteints de pathologie immuno-hématologique actuelle est de 25/an.

Les patients sont informés de façon complète et loyale, en des termes compréhensibles, des objectifs et des contraintes de l'étude, des risques éventuels encourus, des mesures de surveillance et de sécurité nécessaires, de leurs droits de refuser de participer à l'IC ou de la possibilité de se rétracter à tout moment.

CONFIDENTIEL

Toutes ces informations figurent sur un formulaire d'information et de consentement remis au patient. Le consentement libre, éclairé et écrit du patient est recueilli par l'investigateur, ou un médecin qui le représente avant l'inclusion définitive dans l'étude. Un exemplaire du formulaire d'information et des consentements signés par les deux parties est remis au patient, l'investigateur en conserve aussi un original après l'avoir scanné et archivé dans le dossier médical informatisé patient.

6 ORGANISATION GENERALE DE L'ETUDE

6.1 Calendrier de l'étude

- Durée de la période d'inclusion : 12 mois
- Durée de la participation pour chaque sujet : 15 semaines
- Durée totale de l'étude : 16 mois
- Date réelle de début de la recherche : 06/01/2026
- Date théorique de fin de la recherche : 06/05/2027

6.2 Schéma général et tableau récapitulatif

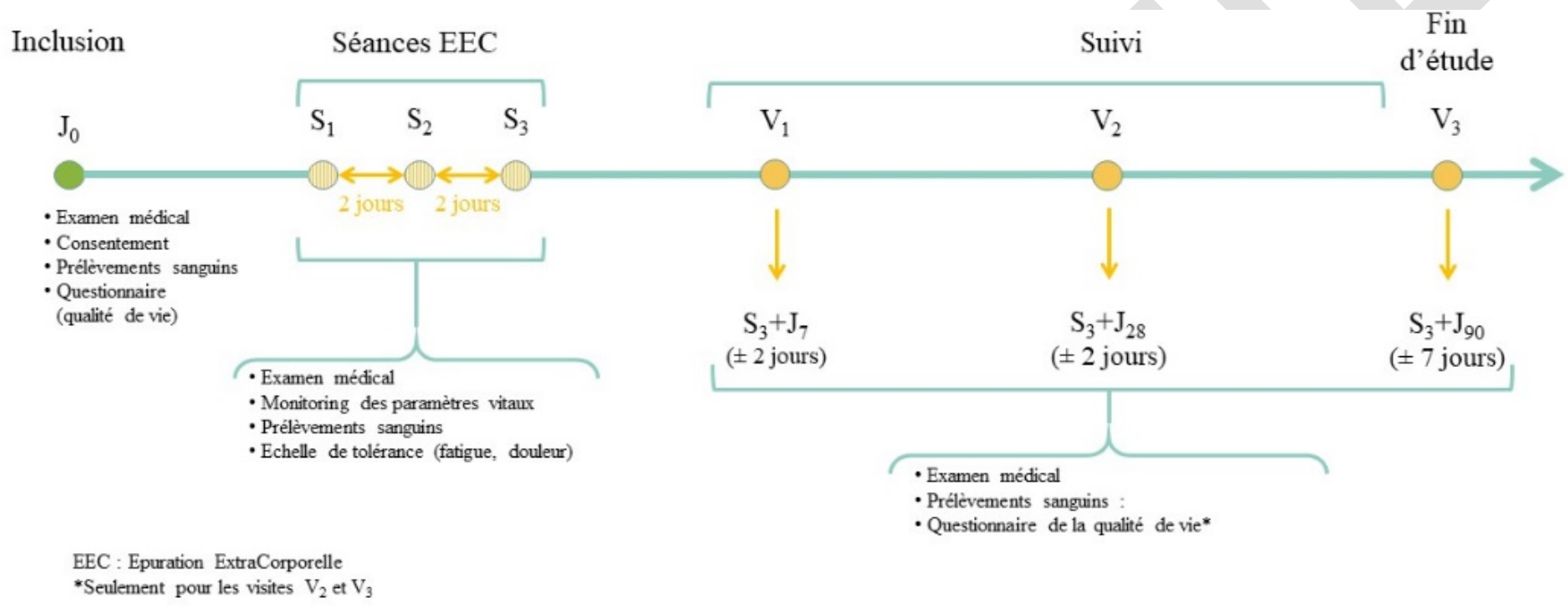

*Figure **2** Schéma général de l'IC MEXIRON*

6.3 Déroulement de l'étude

6.3.1 Screening – inclusion (J_0)

Les patients susceptibles de participer à l'étude sont identifiés au sein du service d'Hématologie Clinique de l'Hôpital Lyon Sud dans le cadre de leur suivi médical régulier. Les patients sont informés par un médecin investigateur du protocole ou un médecin qui le représente. Toute explication nécessaire à la bonne compréhension de l'étude est donnée au patient, ainsi qu'une lettre d'information expliquant les objectifs et le déroulement du protocole.

Le patient est informé de façon complète et loyale, en des termes compréhensibles, des objectifs et des contraintes de l'étude, des risques éventuels encourus, des mesures de surveillance et de sécurité nécessaires, de ses droits de refuser de participer à l'étude ou de la possibilité de se rétracter à tout moment.

L'investigateur remet au patient un formulaire de consentement de participation à l'étude. Il est laissé au patient le délai de réflexion nécessaire (à minima 1 semaine) pour décider de sa participation à l'étude. Un consentement en vue de constituer une biocollection à partir des prélèvements sanguins et de dialysat est également à recueillir auprès du patient.

Les formulaires de consentement doivent être signés avant la réalisation de tout examen clinique ou paraclinique dans le cadre l'étude.

Si le patient donne son accord de participation, le volontaire et l'investigateur datent et signent nominativement (nom et prénom en clair) les formulaires de consentement. Un exemplaire est conservé par le patient, tandis qu'un second est conservé dans le classeur investigateur de l'étude après avoir été scanné et archivé dans le dossier médical informatisé patient.

Pour les femmes en âge de procréer, un prélèvement sanguin (tube PST vert 3 mL) est réalisé pour effectuer un dosage de la gonadotrophine chorionique humaine, hCG afin de vérifier qu'une grossesse n'est pas en cours.

La programmation des séances d'Epuration ExtraCorporelle est alors planifiée en discussion partagée avec le patient.

Le patient complète l'auto-questionnaire MOS SF-12.

Etude ancillaire No.1 : Un prélèvement sanguin veineux (1 tube sec 4mL) est réalisé avant d'être acheminé par un réseau pneumatique au laboratoire d'Analyse de Traces et Métaux Toxiques (Dr François PARANT) sur l'Hôpital Lyon Sud pour y être réceptionné et stocké avant d'être récupéré par l'institut lumière pour des analyses complémentaires : profil multi minéral (sous la responsabilité du Dr Olivier TILLEMENT).

Etude ancillaire No.2 : Des prélèvements sanguins veineux (3 tubes EDTA 4 mL et 1 tube sec 4 mL) sont réalisés avant d'être envoyés au Centre de Ressources Biologiques de l'Hôpital Lyon Sud, sous la responsabilité du Pr Alexandra TRAVERSE-GLEHEN, pour y être réceptionnés, pré-techniqués, aliquotés et stockés (-80°C) sous 2 cryotubes (PBMC), 2 cryotubes (Plasma EDTA) et 2 autres cryotubes (sérum).

CONFIDENTIEL

		Inclusion	Procédures d'Epuration ExtraCorporelle						Suivi		Fin d'étude
			Séance S_1		Séance S_2		Séance S_3				
		J_0	*Début* H_0	*Fin* H_f	*Début* H_0	*Fin* H_f	*Début* H_0	*Fin* H_f	V_1 $S_{3+7 jours}$ *± 2 jours*	V_2 $S_{3+28 jours}$ *± 2 jours*	V_3 $S_{3+90 jours}$ *± 7 jours*
	Vérification des critères d'éligibilité	**X**									
	Examen clinique<br>Taille, poids, IMC, ECOG PS	**X**	**X**		**X**		**X**		**X**	**X**	**X**
	Information patient	**X**									
	Recueil du consentement	**X**									
	Biologie : fer, CST, ferritine, CRP, sodium, calcium, magnésium, NFS *(1 tube PST 4 mL, 1 tube SST 5 mL, 1 tube EDTA 4 mL)*		**X**¶	**X***	**X**¶	**X***	**X**¶	**X***	**X**	**X**	**X**
	Monitoring TAS, TAD, fréquence cardiaque, SaO_2 et température$^{\delta}$		**X**		**X**		**X**				
	Echelle Visuelle Analogique (EVA)			**X**		**X**		**X**			
	Auto-questionnaire MOS SF-12	**X**								**X**	**X**
Etude ancillaire No.1	Prélèvements de 15 mL au niveau de la poche de dialysat *(tube à centrifuger stérile 15 ml exempt de métaux lourds)*		H_0		H_0		H_0				
Etude ancillaire No.1	Prélèvements‡ de 15 mL du liquide de dialyse sur la ligne de l'effluent et 2x15 mL au niveau du sac d'effluent *(tube à centrifuger stérile 15 mL exempt de métaux lourds)*		H_1θ, H_2θ, H_fθ, H_fθ‡ *± 10 minutes*		H_1θ, H_2θ, H_fθ, H_fθ‡ *± 10 minutes*		H_1θ, H_2θ, H_4θ, H_fθ‡ *± 10 minutes*				
Etude ancillaire No.1	Prélèvements sanguins veineux *(tube d'analyse des oligo-éléments 6 mL)*	**X**	H_0	H_f					**X**	**X**	
Etude ancillaire No. 2	Prélèvements sanguins veineux *(3 tubes EDTA 4 mL et 1 tube secs 4 mL)*	**X**				**X**			**X**		**X**
	Recueil et notification EI	←	→	→	→	→	→	→	→	→	→

¶ ± 10 min. du début de la séance

* jusqu'à 10 min après la fin de la séance (H_f) d'Epuration ExtraCorporelle

δ A chaque séance : H_{0+2min}, H_{0+3min}, H_1, H_2, H_4 avant débranchement

θ au niveau de la ligne d'effluent (1 tube)

‡ au niveau du sac de l'effluent (2 tubes)

*Tableau **1** Tableau récapitulatif du déroulement de l'étude*

CONFIDENTIEL

6.3.2 Déroulement d'une séance d'Epuration ExtraCorporelle (S_1, S_2, S_3)

Avant la réalisation de la première séance (S_1) d'Epuration ExtraCorporelle, le choix de l'accès vasculaire et l'évaluation des vaisseaux disponibles sont réalisés par l'équipe du service de néphrologie et de réanimation en charge du patient.

Les séances d'Epuration ExtraCorporelle sont prévues sous forme de cycle unique de 3 séances successives qui sont réparties sur une seule semaine calendaire (délai de 48h +/- 12h entre deux séances). Les séances se déroulent dans le service de néphrologie, dialyse et nutrition rénale de l'Hôpital Lyon Sud sous la supervision de l'investigateur principal ou du coordinateur scientifique (tous deux médecins réanimateurs) avec une prise en charge adaptée si nécessaire. Il sera assisté d'une infirmière de recherche clinique formée à la technique d'hémodialyse continue. Les 3 séances d'Epuration ExtraCorporelle (S_1, S_2 et S_3) suivent un déroulement identique à savoir :

a. Bilan biologique réalisé avant le jour de la séance (J-1 ou J-2), à la convenance du patient (prélèvement par IDE à domicile ou laboratoire de ville) pour réalisation d'une NFS
b. Examen clinique par l'investigateur principal ou un co-investigateur.
c. En cas de thrombopénie <50 G/L identifiée sur le bilan pré-séance, une transfusion plaquettaire sera réalisée avant de débuter la séance
d. Bilan biologique « pré-séance » (S_1H_0, S_2H_0, S_3H_0) [± 10 min. du début de la séance] :
   - NFS : 1 tube EDTA 4 mL
   - Fer, ferritine, CST : 1 tube SST 5 mL
   - Ionogramme sanguin (sodium, potassium, calcium, magnésium), CRP : 1 tube PST 4 mL
e. Branchement du dispositif d'épuration avec double ponction en veine humérale par un cathéter périphérique sur chaque bras (18G en prélèvement, 20G en retour patient) et prise des constantes vitales (pression artérielle, fréquence cardiaque, saturation en oxygène, température).Séances d'Epuration ExtraCorporelle (S_1, S_2 et S_3) réalisées sur une machine d'hémodialyse (multiFiltratePRO, Fresenius Medical Care) dédiée exclusivement et paramétrée spécifiquement pour l'IC :
   - Le dialysat de traitement MEX-CDA est préparé en utilisant 2L de solution multiBic® reconstitue avec 2 doses (2 flacons) de 50 mL de solution MEX-CD1 (Brochure Investigateur)
   - Injection dans le circuit d'une anticoagulation préventive (HEPARINE CHOAY 5000 UI/1 ml) en perfusion continue (IVSE) avec un schéma adapté au taux de plaquette : 10 UI/kg/h chez les patients ayant un taux de plaquettes supérieur à 100 G/L ; 5 UI/kg/h chez les patients ayant un taux compris entre 50 et 100 G/L
   - Réglage du débit sanguin à 70 mL/min et du débit du dialysat MEX-CD1 à 600 mL/h.
   - Durée maximale de la séance réglée sur 3h20 sur moniteur d'hémodialyse, dialyseur pédiatrique AVpaed (Fresenius Medical Care) (0,2 m^2, 30 kDa, QBmin 10-100 mL/min).
   - Monitoring rapproché des paramètres vitaux (pression artérielle, fréquence cardiaque, température) durant toute la durée de la séance d'Epuration ExtraCorporelle : H_{0+2min}, H_{0+3min}, H_1, H_2, H_3 et avant débranchement.
   - Débranchement du dispositif d'épuration.
   - Cotations d'EVA (Echelle Visuelle Analogique) sur la tolérance de la séance [10 : normal - 0 : très fatigué ou malaise intense] et de la douleur ressentie à l'issue de la séance [10 : douleur très forte - 0 : pas de douleur ressentie].
   - ***Etude ancillaire No.1*** : A plus ou moins 10 minutes du début de la séance (H_0) et à la fin (H_f) de la séance d'Epuration ExtraCorporelle 1 (S_1) uniquement, un prélèvement sanguin veineux (1 tube d'analyse des oligo-éléments 6 mL) est réalisé avant d'être acheminé par un réseau pneumatique au laboratoire d'Analyse de Traces et Métaux Toxiques (Dr François PARANT) sur l'Hôpital Lyon Sud pour y être réceptionné et stocké avant d'être récupéré pour des analyses complémentaires : profil multi minéral (sous la responsabilité du Dr Olivier TILLEMENT). Au cours de chaque séance, des prélèvements (tube à centrifuger stérile 15 mL exempt de métaux lourds - Labcon / Dutscher Référence 949892) sont réalisés au niveau de la poche de dialysat (1 tube de 15 mL à H_0), sur la ligne de l'effluent (1 tube de 15 mL à H_1, H_2, H_f) et du sac de l'effluent (2 tubes de 15 mL à H_f) afin de mesurer la quantité de fer

CONFIDENTIEL

chélaté par la solution de MEX-CD1 et la quantité des autres métaux chélatés par la solution de MEX-CD1. Les prélèvements sont acheminés par un réseau pneumatique au laboratoire d'Analyse de Traces et Métaux Toxiques (Dr François PARANT) sur l'Hôpital Lyon Sud pour y être réceptionnés, une partie analysée et une autre aliquotée en 1 tube Eppendorf (1mL) et récupéré par l'institut lumière pour des analyses complémentaires : profil multi minéral (sous la responsabilité du Dr Olivier TILLEMENT). L'un des prélèvements réalisés au niveau du sac de l'effluent est stocké à -20°C avant d'être adressés à la fin de la troisième séance S_3 à MexBrain pour une analyse centralisée.

f. Bilan biologique « post-séance » (S_1H_f, S_2H_f, S_3H_f) [jusqu'à 10 min. après la fin de la séance] :
   - NFS : 1 tube EDTA 4 mL
   - Fer, ferritine, CST : 1 tube SST 5 mL
   - Ionogramme sanguin (sodium, potassium, calcium, magnésium), CRP : 1 tube PSST 4 mL
   - ***Etude ancillaire No.2 :*** Des prélèvements sanguins (3 tubes EDTA 4 mL et 1 tube sec 4 mL) sont réalisés lors de la séance S_2 avant d'être acheminés par un réseau pneumatique au Centre de Ressources Biologiques de l'Hôpital Lyon Sud pour y être réceptionnés, pré-techniqués, aliquotés et stockés (-80°C) sous 2 cryotubes (PBMC), 2 cryotubes (Plasma EDTA) et 2 autres cryotubes (sérum).

g. Une supplémentation en oligoéléments (ampoule JUNIMIN, 10 mL) est injectée au patient après chaque séance d'épuration, une fois que les différents prélèvements sont réalisés.

Pour des raisons organisationnelles et de sécurité, les séances d'Epuration ExtraCorporelle ne peuvent concerner plus d'un patient inclus et traité sur une même semaine.

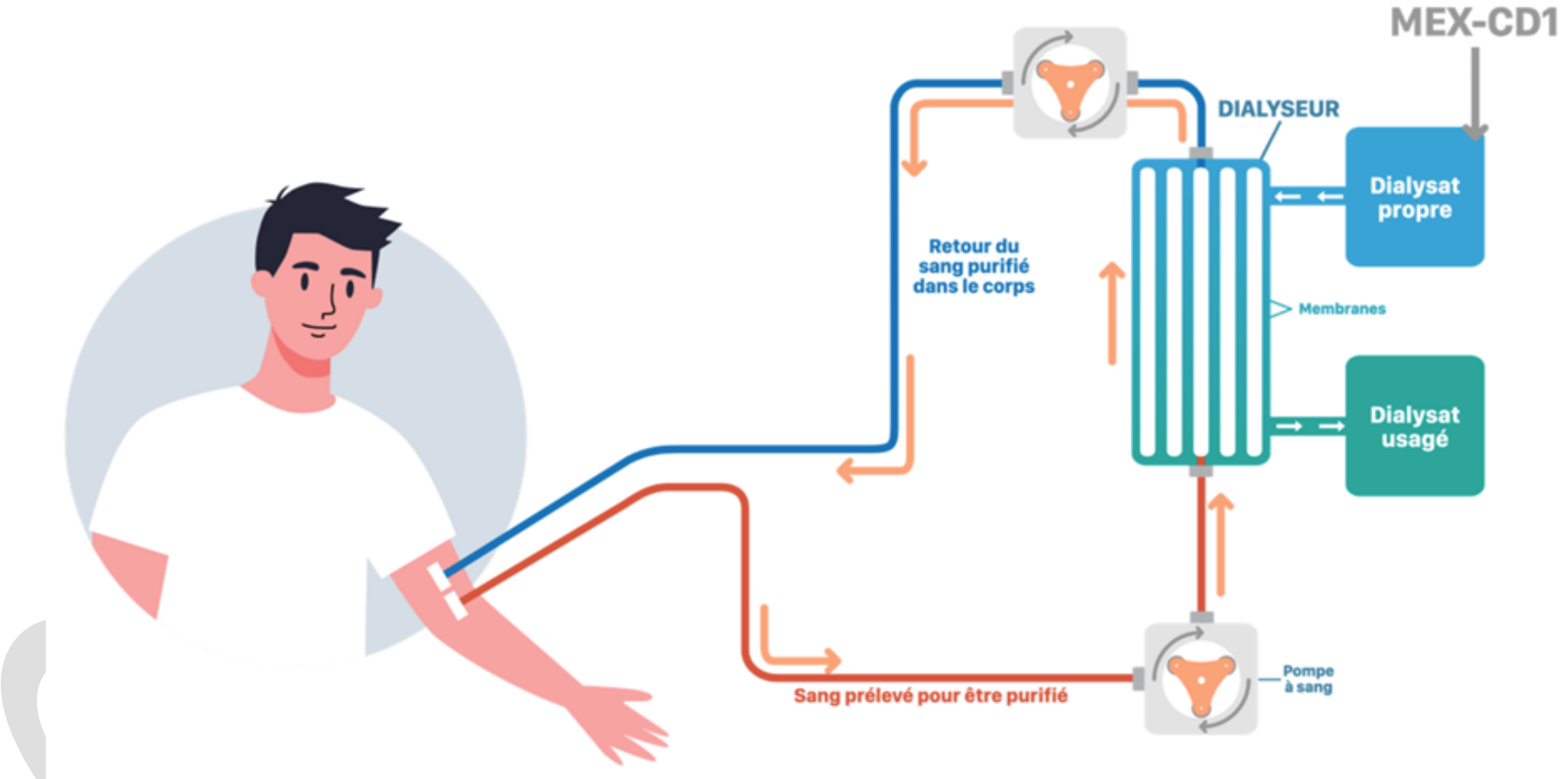

*Figure **3** Schéma de fonctionnement d'une séance d'Epuration ExtraCorporelle*

6.3.3 Visites de suivi (V_1, V_2)

Deux visites de suivi sont réalisées à distance des séances à 7 jours [± 2 jours] (V_1) et à 28 jours [± 2 jours] (V_2) de la 3ème séance (S_3) d'Epuration ExtraCorporelle. Les visites sont réalisées dans le service d'Hématologie Clinique de l'Hôpital Lyon Sud et comprennent :

- Examen clinique et recueil de données cliniques et biologiques.
- Bilan biologique :
  - NFS : 1 tube EDTA 4 mL
  - Fer, ferritine, CST : 1 tube SST 5 mL
  - Ionogramme sanguin (sodium, potassium, calcium, magnésium), CRP : 1 tube PTS 4 mL
  - ***Etude ancillaire No.1*** : Un prélèvement sanguin veineux (1 tube d'analyse des oligo-éléments 6 mL) est réalisé lors de la visite de suivi V1 et V2 avant d'être acheminé par un réseau pneumatique au Laboratoire d'Analyse de Traces et Métaux Toxiques (Dr François Parant) sur l'Hôpital Lyon pour y être réceptionné et stocké avant d'être récupéré par l'institut lumière

pour des analyses complémentaires : profil multi minéral (sous la responsabilité du Dr Olivier TILLEMENT).
  - ***Etude ancillaire No.2*** : Des prélèvements sanguins (3 tubes EDTA 4 mL et 1 tubes secs 4 mL) sont réalisés lors de la **visite V_1** avant d'être acheminés par un réseau pneumatique au Centre de Ressources Biologiques de l'Hôpital Lyon Sud pour y être réceptionnés, pré-techniqués, aliquotés et stockés (-80°C) sous 2 cryotubes (PBMC), 2 cryotubes (Plasma EDTA) et 2 autres cryotubes (sérum).
- Recueil de l'autoquestionnaire MOS SF-12 complété par le patient (uniquement V_2).

6.3.4 Visite de fin de la recherche (V_3)

A l'issue de la visite de fin d'étude (V_3) programmée 90 jours [± 7 jours] après la dernière séance (S_3) d'Epuration ExtraCorporelle, le patient a terminé sa participation à l'étude. Lors de cette dernière visite au sein du service d'Hématologie Clinique de l'Hôpital Lyon Sud, sont réalisés :
- Examen clinique et recueil de données cliniques et biologiques
- Bilan biologique :
  - NFS : 1 tube EDTA 4 mL
  - Fer, ferritine, CST : 1 tube SST 5 mL
  - Ionogramme sanguin (sodium, potassium, calcium, magnésium), CRP : 1 tube PTS 4 mL
  - ***Etude ancillaire No.2*** : Des prélèvements sanguins (3 tubes EDTA 4 mL et 1 tube sec 4 mL) sont réalisés avant d'être acheminés par un réseau pneumatique au Centre de Ressources Biologiques de l'Hôpital Lyon Sud pour y être réceptionnés, pré-techniqués, aliquotés et stockés (-80°C) sous 2 cryotubes (PBMC), 2 cryotubes (Plasma EDTA) et 2 autres cryotubes (sérum)
- Recueil de l'auto-questionnaire MOS SF-12 complété par le patient.

6.4 Procédures cliniques

**Procédures effectuées au cours de l'IC**	**Procédures effectuées dans le cadre du soin**	**Procédures ajoutées du fait de l'investigation**
Traitements	/	Anticoagulation préventive (HEPARINE CHOAY 5000 UI/1 ml) en perfusion continue (IVSE) avec un schéma adapté au taux de plaquette : 10 UI/kg/h chez les patients ayant un taux de plaquettes supérieur à 100 G/L ; 5 UI/kg/h chez les patients ayant un taux compris entre 50 et 100 G/L aux séances d'Epuration Extracorporelle (S_1, S_2, S_3)
	/	Supplémentation préventives en Zinc, Cuivre et Sélénium (spécialité JUNIMIN, ampoule 10mL injectable :6970 µg de gluconate de zinc, 1428 µg de gluconate de cuivre, 40,52 µg de gluconate de manganèse, 13,08 µg de iodure de potassium, 43,81 µg de sélénite de sodium aux séances d'Epuration Extracorporelle (S_1, S_2, S_3)
	Transfusion sanguine dans le service de néphrologie, dialyse et nutrition rénale (Hôpital Lyon Sud)	Séances d'Epuration Extracorporelle (S_1, S_2, S_3) avec le DM MEX-CDI (non marqué CE) dans le service de néphrologie, dialyse et nutrition rénale (Hôpital Lyon Sud) sous la supervision de l'investigateur principal ou du coordinateur scientifique.

CONFIDENTIEL

Consultations	/	- Visite d'inclusion et examen clinique (service d'Hématologie Clinique de l'Hôpital Lyon Sud)<br>- Visites de suivi (V_1, V_2 et V_3) et examen clinique (service d'Hématologie Clinique de l'Hôpital Lyon Sud)
Prélèvements sanguins (4 mL à l'inclusion)		- Dosage hCG à l'inclusion (tube PST 4 mL)
Prélèvements sanguins (26 mL)	/	A l'inclusion :<br>- Fer, CST, ferritine (tube SST 5 mL)<br>En début (H_0¶) et fin (H_f*) de chaque séance d'Epuration Extracorporelle (S_1, S_2, S_3) ainsi que lors des visites de suivi V_1, V_2 V_3 :<br>- CRP et Ionogramme : sodium, potassium, calcium, magnésium (tube PST 4 mL),<br>- NFS (tube EDTA 4mL)<br>- Fer, CST, ferritine (tube SST 5 mL)
Questionnaire - échelle	/	- Auto-passation d'une EVA en fin de séance d'Epuration ExtraCorporelle (S_1, S_2, S_3)<br>- Auto-questionnaire de qualité de vie MOS SF-12 à l'inclusion (J_0), V_2 et V_3
**Etude ancillaire No. 1**<br>Prélèvement sanguin veineux (6 mL)		Prélèvement veineux (tube d'analyse des oligo-éléments 6 mL) à l'inclusion J_0, au début et à la fin de la première séance d'Epuration ExtraCorporelle (S_1H_0, S_1H_f), lors de la visite de suivi V_1 et V_2
**Etude ancillaire No. 1**<br>Prélèvement au niveau de la poche de dialysat (15 mL)		A chaque début de séance (S_1H_0, S_2H_0 et S_3H_0) prélèvement au niveau de la poche de dialysat d'1 tubes à centrifuger stérile 15 mL exempt de métaux lourds - Labcon / Dutscher Référence 949892
**Etude ancillaire No. 1**<br>Prélèvement au niveau de la ligne d'effluent (45 mL)	/	A chaque séance (S_1, S_2 et S_3) prélèvement sur la ligne de l'effluent toutes les heures (H_1, H_2, H_f) d'1 tube à centrifuger stérile 15 mL exempt de métaux lourds - Labcon / Dutscher Référence 949892
**Etude ancillaire No. 1**<br>Prélèvement au niveau du sac d'effluent (30 mL)		A chaque fin de séance (S_1H_f, S_2H_f et S_3H_f) prélèvement au niveau du sac de l'effluent de 2 tubes à centrifuger stérile 15 mL exempt de métaux lourds - Labcon / Dutscher Référence 949892
**Etude ancillaire No. 2**$^{\Delta}$<br>Constitution d'une biocollection $^{\Delta}$	/	Pour chaque patient inclus, sont prélevés à l'inclusion, S_2, V_1 et V_3 :<br>- Sang total : 3 tubes EDTA (total :12 mL)<br>- Sang total : 1 tube sec (total : 4 mL)

$^{\Delta}$ Sur la totalité des patients inclus et ayant bénéficié d'au moins deux séances d'Epuration ExtraCorporelle.¶ ± 10 min. du début de la séance

* Jusqu'à 10 min après la fin de la séance (H_f) d'Epuration ExtraCorporelle

6.5 Collection d'échantillon biologique (étude ancillaire No. 2)

Un consentement en vue de constituer une biocollection à partir des prélèvements sanguins veineux et de dialysat sera recueilli auprès du patient (case à cocher sur le consentement de l'étude principale).

L'objectif de cette collection est de disposer d'échantillons biologiques (sang veineux) pour estimer l'effet de la déplétion martiale (fer) des séances d'Epuration Extracorporelle sur les programmes

CONFIDENTIEL

transcriptomiques des éléments matures circulant (différenciation) afin de répondre aux objectifs de l'étude.

En cours d'étude, aucune mise à disposition ou cession des échantillons collectés n'est permise.

Cette collection est hébergée au Centre de Ressources Biologiques (Hôpital Lyon Sud), sous la responsabilité du Pr Alexandra TRAVERSE-GLEHEN.

Les visites concernées par cette collection biologique et la nature des échantillons sont :

- Visite d'inclusion (J_0) : prélèvements sanguins veineux (3 tubes EDTA 4 mL et 1 tube sec 4 mL) ;
- EEC- séance S_2 : prélèvements sanguins veineux (3 tubes EDTA 4 mL et 1 tube sec 4 mL) ;
- Visite de suivi (V_1) : prélèvements sanguins veineux (3 tubes EDTA 4 mL et 1 tube sec 4 mL) ;
- Visite fin d'étude (V_3) : prélèvements sanguins veineux (3 tubes EDTA 4 mL et 1 tube sec 4 mL).

Les échantillons seront codés (numéro d'inclusion du patient – temps de prélèvement) et apportés en main propre par l'infirmière de recherche clinique au CRB lequel doit être prévenu en amont des visites/séances.

A réception, les échantillons sont pré-techniqués, aliquotés et stockés (congélation dans l'azote des cryotubes PBMC et à -80°C des cryotubes de plasma et sérum) jusqu'à 1 an après la fin de l'étude (cf. paragraphe 2.1.3).

6.6 Plan de surveillance / Contrôle qualité

Un ARC mandaté par le promoteur s'assurera de la bonne réalisation de l'investigation clinique, du recueil des données générées par écrit, de leur documentation, enregistrement et rapport, en accord avec les POS mises en application au sein de la DRS des Hospices Civils de Lyon et conformément aux BPC ainsi qu'aux dispositions législatives et réglementaires en vigueur.

L'investigateur et les membres de son équipe acceptent de se rendre disponibles lors des visites de Contrôle de Qualité effectuées à intervalles réguliers par l'ARC mandaté par le Promoteur. Lors de ces visites, les éléments suivant pourront être revus en fonction du niveau de monitoring adapté à l'étude et déterminé conformément aux POS du promoteur, selon le plan de monitoring :

- Consentement éclairé ;
- Respect du protocole de l'investigation clinique et des procédures qui y sont définies ;
- Qualité des données recueillies dans le cahier d'observation : exactitude, données manquantes, cohérence des données avec les documents sources ;
- Réalisation de la procédure expérimentale ;
- Déclaration des événements indésirables graves et des défectuosités.

Toute visite fera l'objet d'un rapport de monitoring et d'un suivi des actions adressé à l'investigateur du centre visité et à la structure de coordination de la recherche.

D'autre part, les investigateurs s'engagent à accepter les audits d'assurance qualité effectués par des personnes mandatées par le promoteur, ainsi que les inspections effectuées par les Autorités Compétentes. Toutes les données, tous les documents et rapports peuvent faire l'objet d'audits et d'inspections réglementaires sans que puisse être opposé le secret médical.

7 CONSIDERATIONS STATISTIQUES

7.1 Justification de la taille de l'échantillon

Il s'agit d'une étude pilote, monocentrique, chez des patients présentant une surcharge en fer, nécessitant une stratégie de déplétion en fer, quelle que soit l'hémopathie, l'hémoglobinose ou toute autre pathologie d'origine immuno-hématologique sous-jacente, portant sur un nombre limité de 10 patients (avec cycle complet de 3 séances d'Epuration ExtraCorporelle) qui doit permettre d'estimer la faisabilité logistique de l'intervention proposée, d'obtenir des premières données cliniques et biologiques du traitement, sa sécurité et la quantité de fer extraite au cours des séances d'Epuration ExtraCorporelle chez des patients présentant une surcharge martiale nécessitant une stratégie de

CONFIDENTIEL

déplétion associée à une intolérance, une contre-indication ou une inefficacité à un traitement chélateur oral ou sous cutané. Pour palier d'éventuelles sorties prématurées, nous prévoyons d'inclure 13 patients.

7.2 Population d'analyse

La population d'analyse correspondra à tous les patients qui auront eu au moins une séance de dialyse.

7.3 Méthode statistiques

Les caractéristiques des patients, celle de la maladie et la biologie à l'inclusion seront décrites selon les statistiques usuelles définies ci-dessous.

Les données quantitatives seront décrites pour l'ensemble de la population, à l'aide des statistiques descriptives suivantes : l'effectif, le nombre de valeurs manquantes, la moyenne, l'écart type, la médiane, le premier et le troisième quartile ainsi que le minimum et le maximum. Ces statistiques seront considérées comme les statistiques usuelles pour l'analyse de variables quantitatives. Les variables quantitatives pourront être catégorisées selon un *cut-off* prédéfini. Les intervalles de confiance fournis seront des intervalles de confiance bilatéraux à 95%.

Les variables qualitatives seront résumées pour l'ensemble de la population, à l'aide des statistiques descriptives suivantes : l'effectif et le pourcentage de valeurs manquantes, les fréquences et les pourcentages pour chaque niveau de la variable (les valeurs manquantes ne seront pas inclues dans le dénominateur du calcul des fréquences). Ces statistiques seront considérées comme les statistiques usuelles pour l'analyse de variables qualitatives. Lorsque nécessaire, les intervalles de confiance pour les proportions seront calculés à partir d'une loi binomiale exacte.

Une description graphique longitudinale des biomarqueurs sera effectuée.

7.3.1 Critère de jugement principal

La quantité de fer libre chélaté à la fin des trois séances d'Epuration ExtraCorporelle sera estimé par la formule suivante : [fer (µg/L) du sac d'effluent (H_f) - fer (µg/L) de la poche de dialysat (H0)] x volume d'effluent (L). Cette quantité sera décrite selon la moyenne, écart-type, médiane, valeur maximale et minimale.

7.3.2 Critères de jugements secondaires

1) La cinétique de la chélation du fer libre sur la ligne d'effluent (H_1, H_2, H_f) au cours de chaque séance d'Epuration ExtraCorporelle (S_1, S_2, S_3) sera représentée graphiquement et décrite selon leur moyenne, écart-type, médiane, valeur maximale et minimale

2) L'effet de l'Epuration ExtraCorporelle du MEX-CD1 sur les paramètres du bilan martial à court terme sera réalisée par l'estimation des variations de fer libre, ferritine, CST mesurées entre le début et la fin de chaque séance d'EEC (S_1, S_2, S_3) en différence absolue ainsi qu'en pourcentage de variation. Un modèle à effet mixte sera réalisé avec un intercept aléatoire pour estimer la moyenne en prenant en compte les données répétées. Un second modèle sera réalisé en ajoutant l'effet séance en effet fixe. Les variations des valeurs de biomarqueurs (fer libre, ferritine, CST) entre le début de la 1ère séance (S_1H_0) et la fin de la 3ème séance (S_3H_f) seront analysées en différence absolue ainsi qu'en pourcentage de variation. Les variations de fer libre et CST mesurées entre la fin de la dernière séance (S_3H_f) et V_1 (S_3 +J_7) / V_2 (S_3 +J_{28}) / V_3 (S_3 +J_{90}) seront analysées en différence absolue ainsi qu'en pourcentage de variation. Un modèle linéaire à effets mixtes sera réalisé, afin de prendre en compte un effet patient et temps. Le modèle inclura, en effet fixe, le temps (date de la visite) et, en effets aléatoires, un intercept et une pente aléatoire. L'ampleur d'effet de l'intervention sera testée par un test de Wald ; l'intervalle de confiance à 95% de la différence de moyenne sera fourni. Une transformation éventuelle des données pourra être effectuée afin de satisfaire les conditions de normalité du modèle.

3) La tolérance des séances d'Epuration ExtraCorporelle par le MEX-CD1 sera évaluée avec le nombre d'évènements indésirables de tout type et notamment les Evènements d'Intérêt Particulier (EIP), en nombre total, en % d'évènements, ainsi qu'en patients ayant eu au moins un évènement. Les

CONFIDENTIEL

évènements imputables seront décrits spécifiquement. Une description par grade d'intensité, par SOC et PT, sera effectuée.

4) L'effet de l'Epuration ExtraCorporelle avec le MEX-CD1 sur les besoins transfusionnels sera évalué avec le nombre de CGR transfusés décrit par la moyenne, écart-type, médiane, valeur maximale et minimale ou en répartition selon la distribution du nombre de transfusions

5) La faisabilité du traitement expérimental sera évaluée par la proportion de patients ayant réalisé la première séance d'Epuration ExtraCorporelle dans son entièreté, la proportion de patients ayant réalisé un cycle complet de 3 séances d'Epuration ExtraCorporelle consécutives, le ratio temps réel (heure) sous traitement expérimental / temps programmé (3h20) et le volume total (litre) de sang traité (donnée fournie par l'équipement de dialyse). Les données quantitatives seront décrites selon leur moyenne, écart-type, médiane, valeur maximale et minimale

6) La tolérance globale de la séance ressentie par le patient sera mesurée par 2 échelles EVA : Asthénie/malaise ressenti et Douleur ressentie. Les scores seront décrits selon leur moyenne, écart-type, médiane, valeur maximale et minimale à chaque séance. L'évolution du score entre les séances sera représentée graphiquement pour chaque patient.

7) L'évolution de la qualité de vie ressentie par le patient distance du cycle de séances d'Epuration ExtraCorporelle sera mesurée par l'auto-questionnaire MOS SF-12 à l'inclusion, V_2 et V_3. Le score total sera calculé. Les scores seront décrits selon leur moyenne, écart-type, médiane, valeur maximale et minimale à chaque visite. L'évolution du score entre les visites sera représentée graphiquement pour chaque patient.

7.4 Analyses intermédiaires

Il n'y a pas d'analyse intermédiaire prévue.

7.5 Méthode de prise en compte des données manquantes

Il n'est pas prévu d'imputation pour les données manquantes

Les données aberrantes feront l'objet d'une demande de confirmation auprès du centre investigateur. En cas de confirmation, leur valeur ne sera pas modifiée, et sera prise en compte telle quelle lors de l'analyse.

7.6 Gestion des modifications apportées au plan d'analyse

Un plan d'analyse statistique détaillé sera rédigé avant le gel de la base des données. Il tiendra compte de toute modification du protocole ou de tout événement inattendu survenu au cours de l'investigation clinique et ayant un impact sur les analyses présentées ci-dessus. Les analyses planifiées pourront être complétées en cohérence avec les objectifs de l'étude.

Toute modification apportée par la suite au plan d'analyse statistique devra être justifiée et donnera lieu à une nouvelle version du document. Ces déviations au plan d'analyse seront reportées dans le rapport final de l'étude. L'ensemble des documents sera conservé dans le dossier de l'étude.

7.7 Responsable des analyses

Les analyses seront réalisées par le service de Biostatitiques et Bioinformatique du Pôle Santé Publique des Hospices Civils de Lyon du Pr Delphine MAUCORT-BOULCH (162 Avenue Lacassagne 69424 LYON Cedex).

8 DROITS D'ACCES AUX DONNEES ET DOCUMENT SOURCES

8.1 Accès aux données

- Le promoteur est chargé d'obtenir le consentement de l'ensemble des parties impliquées dans la recherche afin de garantir l'accès direct à tous les lieux de déroulement de l'investigation, aux

CONFIDENTIEL

données sources, aux documents sources et aux rapports dans un but de contrôle de qualité et d'audit par le promoteur ;

- Les investigateurs mettront à disposition des personnes chargées du suivi, du contrôle de qualité ou de l'audit de la recherche, les documents et données individuelles strictement nécessaires à ce contrôle, conformément aux dispositions législatives et réglementaires en vigueur (articles L.1121-3 et R.5121-13 du code de la santé publique).

8.2 Documents sources

Les documents sources sont définis comme tout document ou objet original permettant de prouver l'existence ou l'exactitude d'une donnée ou d'un fait enregistrés au cours de l'IC. Ils seront conservés pendant 10 ans après la fin de la recherche par le promoteur et l'investigateur ou par l'hôpital s'il s'agit d'un dossier médical hospitalier. Sont considérés comme documents sources :

- Dossiers médicaux ;
- Comptes rendus de laboratoire ;
- Enregistrement de la machine de dialyse ;
- Echelle Visuelle Analogique ;
- Auto-questionnaire de qualité de vie MOS SF-12 ;
- Copie ou transcription authentifiée ;
- Echantillons biologiques.

8.3 Confidentialité des données / CNIL

Cette étude entre dans le cadre de la « Méthodologie de Référence » (MR-001) en application des dispositions de l'article 54 alinéa 5 de la loi n°78-17 du 6 janvier 1978 modifiée relative à l'informatique, aux fichiers et aux libertés. Ce changement a été homologué par décision du 5 janvier 2006 et modifiée le 21 juillet 2016. Les Hospices Civils de Lyon, promoteur de l'étude, ont signé un engagement de conformité à cette « Méthodologie de Référence ».

Conformément aux dispositions concernant la confidentialité des données auxquelles ont accès les personnes chargées du contrôle de qualité d'une recherche impliquant le personne humaine (article L.1121-3 du code de la santé publique), conformément aux dispositions relatives à la confidentialité des informations concernant notamment les IC, les personnes qui s'y prêtent et les résultats obtenus (article R. 5121-13 du code de la santé publique), les personnes ayant un accès direct aux données prennent toutes les précautions nécessaires en vue d'assurer la confidentialité des informations relatives aux investigations cliniques, aux personnes qui s'y prêtent et notamment en ce qui concerne leur identité ainsi qu'aux résultats obtenus.

Ces personnes, au même titre que les investigateurs eux-mêmes, sont soumises au secret professionnel (selon les conditions définies par les articles 226-13 et 226-14 du code pénal).

Pendant la recherche impliquant la personne humaine ou à son issue, les données recueillies sur les personnes qui s'y prêtent et transmises au promoteur par les investigateurs (ou tous autres intervenants spécialisés) sont codées.

Elles ne doivent en aucun cas faire apparaître en clair les noms des personnes concernées ni leur adresse.

Seules la première lettre du nom du sujet et la première lettre de son prénom sont enregistrées, accompagnées d'un numéro codé propre à l'étude indiquant l'ordre d'inclusion des sujets.

Le promoteur s'assure que chaque personne qui se prête à la recherche a donné son accord par écrit pour l'accès aux données individuelles la concernant et strictement nécessaires au contrôle de qualité de la recherche.

8.4 Conformité à la Norme IS0 14155

Considérant l'article 62 du Règlement européen du Dispositif médical (RDM) 2017/745 sur les dispositions de conformité régissant les investigations cliniques, le promoteur et l'investigateur, en signant ce protocole, s'engagent à ce que la présente investigation clinique soit conduite :

CONFIDENTIEL

- Conformément au Règlement européen du Dispositif médical (RDM) [(UE) 2017/745 relatif aux dispositifs médicaux] ainsi qu'aux dispositions prises pour son application en France ;
- Conformément au protocole ;
- Conformément aux recommandations du *Medical Device Coordination Group* (MDCG) applicables concernant les investigations cliniques et notamment concernant le contenu de la Brochure pour l'Investigateur ;
- Conformément aux BPC ICH-E6 dans leur dernière version en vigueur pour les parties applicables ;
- Conformément à la déclaration d'Helsinki de l'Association médicale mondiale sur les principes éthiques applicables à la recherche médicale impliquant des êtres humains ;
- Conformément au Règlement Général sur la Protection de Données (RGPD) et conformément à la Loi Informatique et Liberté (LIL) concernant le traitement des données ;
- Conformément aux dispositions législatives et réglementaires actuellement en vigueur en France ;
- Conformément à la norme ISO 9001, certification du promoteur depuis 2018.

Considérant ces engagements de respect de conformité, nous concluons que le niveau de conduite de l'étude sera mené avec un niveau équivalent à la norme ISO 14155 concernant la sécurité, la qualité et la robustesse des données.

9 GESTION ET CONSERVATION DES DONNEES

9.1 Gestion des modifications

En cas de modification substantielle apportée au protocole par l'investigateur, elle sera approuvée par le promoteur. Ce dernier devra obtenir préalablement à sa mise en œuvre un avis favorable du CPP et une autorisation de l'ANSM, dans le cadre de leurs compétences respectives.

9.2 Ecarts au protocole

Tout écart au protocole sera relevé et fera l'objet d'une action correctrice et sera pris en compte pour l'analyse finale.

9.3 Cahier d'observation (électronique)

Les données de l'étude seront recueillies dans un cahier d'observation électronique (eCRF). Cet eCRF, spécifique à l'étude, sera développé par un datamanager des Hospices Civils de Lyon sur le logiciel Ennov Clinical® 8.2.50. Ce logiciel respecte les recommandations de la FDA sur les systèmes informatisés pour la gestion des essais cliniques (Guidance for Computerized Systems Used in Clinical Trials) ainsi que les recommandations FDA sur la signature électronique (21CFR part 11).

L'eCRF comportera uniquement les données nécessaires à la réalisation du protocole, à l'analyse prévue au protocole et à la publication scientifique. Les autres données des patients, nécessaires à leur suivi en dehors de cette étude, seront collectées dans le dossier médical du patient.

Les données de l'étude seront informatisées de façon codée, conformément à la loi informatique et liberté. Les sujets/patients de l'étude seront identifiés par un numéro unique d'inclusion dans l'étude et par la première lettre de leur nom et de leur prénom. Une liste d'identification des participants à la recherche sera conservée dans le classeur investigateur de chaque centre.

Les données des patients seront saisies sur l'eCRF, au fur et à mesure de leur collecte, par les personnes autorisées (investigateur et personnes apparaissant sur la délégation de tâches) et disposant de leurs propres identifiants, conformément à la loi informatique et liberté.

Le remplissage du cahier d'observation via internet permettra au centre de coordination et au promoteur de l'étude de visualiser rapidement et à distance les données.

L'investigateur est responsable de l'exactitude, de la qualité et de la pertinence de toutes les données saisies. A ce titre, chaque page du CRF patient devra être signée électroniquement par l'investigateur, signifiant ainsi son accord et sa responsabilité vis-à-vis des données collectées.

CONFIDENTIEL

9.4 Gestion des données

Les données seront collectées par simple saisie dans l'eCRF par le personnel dédié à l'étude.

L'accès aux données sera sécurisé et restrictif selon les droits octroyés à chaque utilisateur. Les codes de connexion seront personnels et confidentiels et seront constitués par un identifiant et un mot de passe, dont la complexité respectera les recommandations de la CNIL en vigueur, et qui sera connu seulement de l'utilisateur.

Les données seront validées conformément au plan de validation des données.

Lors de la saisie des données, le système exécutera immédiatement des pré-contrôles de présence/ absence et de cohérence des données. Toute information manquante ou incohérente fera l'objet d'un message pop-up et/ou warning invitant l'utilisateur à vérifier et si besoin, corriger la donnée saisie directement sur l'eCRF.

En parallèle de ce système interactif, des tests programmés et définis dans le plan de validation des données seront périodiquement exécutés sur la base de données afin de détecter les erreurs résiduelles qui n'auraient pas été corrigées en temps réel. Dans ce cas, des demandes de correction seront éditées et mises en ligne sur l'eCRF à l'attention des centres investigateurs.

Tous les moyens nécessaires seront mis en œuvre pour récupérer les données manquantes notamment sur les critères de jugement. En cas de données définitivement manquantes, il sera demandé d'utiliser les codes de données manquantes prédéfinis dans le système à savoir :

- NA ou A (pour Non Applicable) pour les données non applicables ;
- NK ou K (pour Not Known) pour les données non connues ;
- ND ou D (pour Not Done) pour les mesures non faites.

Un guide de saisie précisera éventuellement les autres codes de données manquantes à utiliser et comment les utiliser.

Un audit trail intégré au logiciel, permettra de tracer toutes les interventions sur la base de données (saisie des données, correction des données, modification des droits etc.) et enregistrera la date et l'heure de l'intervention, la personne qui l'a effectuée, l'action effectuée, l'état avant et l'état après l'intervention. Pour toute modification, la raison de la modification est requise.

Des bilans d'avancement mis à jour à fréquence définie, seront proposés à l'équipe projet de manière à suivre l'avancement global du projet et détecter des éventuels problèmes dans le recueil des données.

Préalablement au transfert des données au statisticien pour analyse, et en amont du gel de la base, une réunion de revue des données réunissant l'investigateur principal, la coordination et les personnes en charge de la gestion et de l'analyse des données sera programmée. Cette réunion permettra d'effectuer un état de la base, de contrôler la qualité des données et de statuer sur d'éventuelles incohérences résiduelles. Dans certains cas, plusieurs réunions de revue des données pourront être nécessaires pour revoir les données.

Le gel de la base de données sera effectué sur données consolidées, après signature du plan d'analyse statistique et après accord de l'investigateur coordonnateur, du promoteur et des personnes en charge de la gestion et de l'analyse des données.

Après le gel de la base de données, aucune modification des données ne pourra être effectuée sans dégel de la base. Le dégel de la base de données ne sera possible que dans des circonstances extraordinaires. La demande devra être justifiée et les modifications précisées dans un document écrit, approuvé par le promoteur ou son représentant.

L'analyse statistique des données se fera sur données consolidées, après gel de base.

Les transferts de données se feront via le service de partage de fichiers des Hospices Civils de Lyon dans la mesure du possible.

- Pour les transferts intra-HCL, les données seront rendues disponibles uniquement à son destinataire, qui pourra les récupérer via son mot de passe HCL.
- Pour les transferts hors-HCL, les données seront chiffrées et sécurisées par mot de passe, lequel sera transmis au destinataire uniquement, indépendamment de l'envoi du lien du fichier.

CONFIDENTIEL

Les fichiers de données, exportés à partir de l'eCRF en vue des analyses statistiques, seront stockés sur un serveur sécurisé avec accès limité aux gestionnaires des données et statisticiens.

Les données de l'eCRF seront hébergées dans un Datacenter géré par la société OVH (prestataire d'Ennov Clinical). Une sauvegarde quotidienne des données sera réalisée la nuit sur un serveur dédié aux études Ennov Clinical des Hospices Civils de Lyon (HCL), et conservée pendant 4 semaines. Une copie de cette sauvegarde sera stockée sur un site géographique différent.

A la fin de l'étude, les eCRF des patients signés par l'investigateur en charge du patient seront imprimés en pdf pour archivage par le centre.

Le plan de data management décrivant tout le processus de gestion des données sera rédigé par le data manager en charge du projet en collaboration avec le promoteur, l'équipe de coordination, et/ou l'investigateur principal, le statisticien.

9.5 Plan de data management

Le plan de data management de l'étude sera rédigé par le data manager en charge du projet, conformément au plan suivant :

1 Abréviations
2 Généralités - Responsabilités
3 Informations sur le projet
4 Nature des données recueillies
5 Confidentialité et mot de passe
    5.1 Accès limité et contrôlé au logiciel pour le paramétrage de l'étude
    5.2 Accès limité et contrôlé à l'eCRF pour la saisie des données et le suivi de l'étude
    5.3 L'audit trail
    5.4 Les profils el les droits des utilisateurs
    5.5 Gestion des levées d'insu
6 Organisation et validation des données
    6.1 Organisation des données
    6.2 Conventions de saisie
    6.3 Contrôle de la base avant mise en production
    6.4 Contrôles des données
7 Alertes mails
8 Réconciliation des EIG
9 Exports - Revue des données - Gel de la base de données
    9.1 Export des données
    9.2 Revue des données
    9.3 Gel de la base
10 Archivage

Le plan de data management sera revu et validé par le promoteur, l'équipe de coordination, et/ou l'investigateur principal, le statisticien.

9.6 Archivage

Tous les documents de l'étude seront archivés pendant la durée prévue par le règlement européen du Dispositif médical (RDM) 2017/745 soit 10 ans après la fin de l'essai.

Le promoteur conservera tous les documents essentiels de l'essai (TMF). L'archivage promoteur sera organisé selon le système d'assurance qualité de celui-ci.

L'investigateur principal de chaque centre investigateur conservera pendant 10 ans après la notification de la fin de l'essai par le promoteur les documents suivants:

- Protocole et annexes, amendements éventuels,
- Formulaires d'information et consentements originaux signés
- Données individuelles (copies authentifiées de données brutes)
- Documents de suivi et courriers relatifs à la recherche
- Analyses statistiques
- Rapport final de l'étude

CONFIDENTIEL

Aucun déplacement ou destruction ne pourra être effectué sans l'accord du promoteur. Au terme des 10 ans, le promoteur sera consulté pour destruction.

Toutes les données, tous les documents et rapports pourront faire l'objet d'audit ou d'inspection.

10 CONSIDERATIONS ETHIQUES

10.1 Période d'exclusion et interdiction de participer à une autre étude clinique

A l'issue de l'étude, il n'est pas prévu de période d'exclusion avant participation éventuelle à une autre étude. Le cas échéant, un patient inclus dans l'IC MEXIRON ne doit pas participer simultanément à une autre recherche interventionnelle susceptible d'impacter les résultats de l'IC.

10.2 Indemnisation des sujets et inscription au fichier national des personnes se prêtant à une recherche interventionnelle

Il n'est pas prévu d'indemniser les volontaires qui accepteront de participer à l'étude. L'objet de la recherche étant en rapport avec l'état pathologique des sujets, l'inscription au fichier national des Volontaires se prêtant à une Recherche Biomédicale (VRB) n'est pas requise.

10.3 Remboursement des sujets

Les sujets qui ont accepté de participer à l'étude, sont remboursés des coûts relatifs à leurs déplacements (visites sur site en lien avec le protocole de l'étude) sur la base des barèmes et forfaits préétablis (tenant compte de la distance parcourue et du type de transport utilisé, véhicule personnel ou transport public).

10.4 Autorités compétentes

Le protocole, la notice d'information et le formulaire de consentement de l'étude ont été soumis pour avis au CPP.

La notification de l'avis favorable du CPP a été transmise au promoteur de l'étude et à l'ANSM. Une demande d'autorisation d'étude a été également adressée par le promoteur à l'ANSM.

Le promoteur s'est engagé à ce que le démarrage de l'étude ne se fasse qu'après obtention de l'avis favorable du CPP et de l'autorisation d'étude de l'ANSM.

10.5 Modifications substantielles

En cas de modification substantielle apportée au protocole par l'investigateur, elle sera approuvée par le promoteur. Ce dernier devra obtenir préalablement à sa mise en œuvre un avis favorable du CPP et une autorisation de l'ANSM si requise dans le cadre de leurs compétences respectives. Un nouveau consentement des personnes participant à la recherche sera recueilli si nécessaire.

11 RESPONSABILITES CONCERNANT LE DISPOSITIF

La Pharmacie à Usage Intérieur (PUI) de l'Hôpital Lyon Sud (Service de Pharmacie – Unité des Dispositifs Médicaux Stériles – Pharmacien Responsable : Dr Cédric BESNIER) est responsable du circuit d'approvisionnement et de la traçabilité sanitaire de la réception et de la dotation du dispositif médical expérimental au service de néphrologie, dialyse et nutrition rénale. La PUI n'est pas responsable de son utilisation dans le service.

11.1 Fabrication

Le dispositif MEX-CD1 de classe III est fabriqué par la société MexBrain (13 avenue Albert Einstein - 69100 Villeurbanne).

11.2 Conditionnement et étiquetage

Le produit (MEX-CD1) est conditionné sous forme de boite unitaire (Figure **3**) contenant :

CONFIDENTIEL

- 1 boîte de 20 flacons de MEX-CD1 (Figure **4**), solution polymérique stérile de 50 mL (dispositif médical expérimental),
- La brochure investigateur (BI).

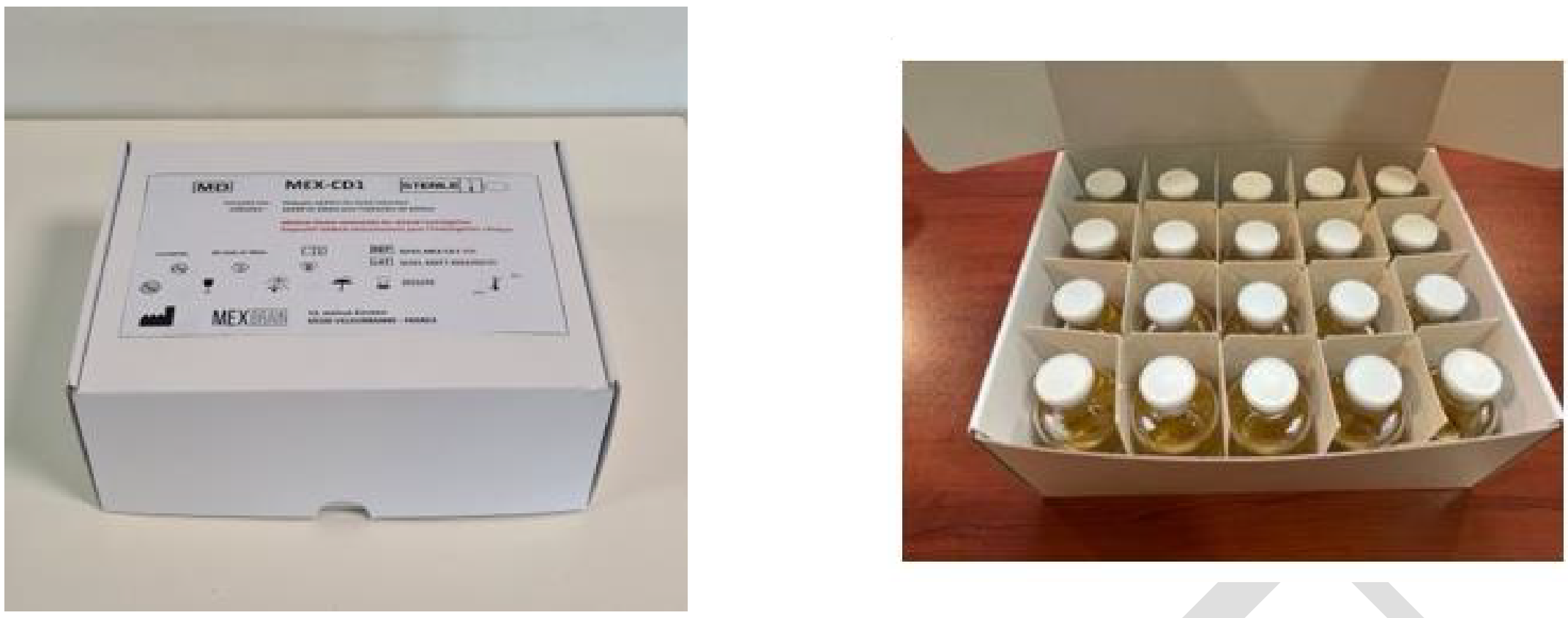

*Figure **4** Conditionnement du MEX-CDI (boîte)*

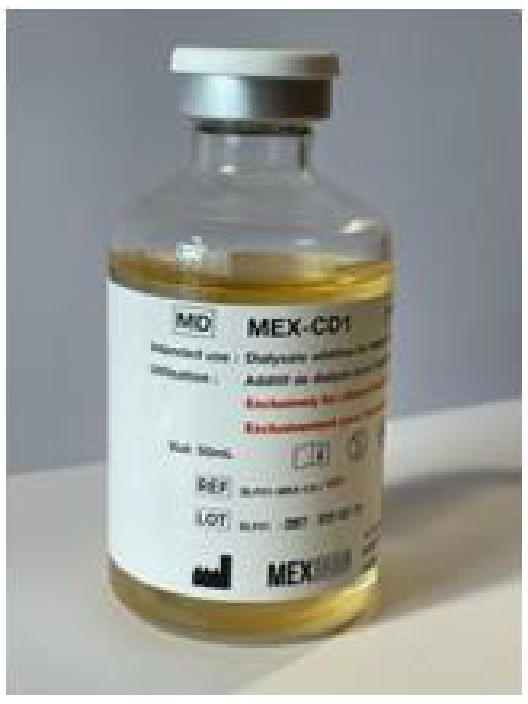

*Figure **5** Conditionnement du MEX-CDI (flacon)*

Chaque DM sera conditionné et étiqueté conformément à la réglementation des essais cliniques en vigueur, par l'Unité des Dispositifs Médicaux Stériles du service de Pharmacie (Bâtiment 3A, Hôpital Lyon Sud – Pharmacien Responsable : Dr Cédric Besnier).

11.3 Gestion du dispositif

Le dispositif MEX-CD1 est fourni par la société MexBrain au site investigateur. L'étude se déroulant uniquement dans des services de l'hôpital Lyon Sud, il n'y aucun autre site à approvisionner.

Un envoi initial de 3 boites de 20 flacons de MEX-CD1 réalisé en amont de la mise en place de l'IC, permet de réaliser les séances d'Epuration ExtraCorporelle pour les quatre premiers patients inclus. Une demande de réapprovisionnement de 3 boites de 20 flacons de MEX-CD1 est à prévoir après l'inclusion du quatrième patient puis après l'inclusion du huitième patient. Au total, 9 boites de 20 flacons de MEX-CD1 seront approvisionnés au cours de l'étude. Les demandes de réapprovisionnement sont à adresser directement au centre de coordination de l'étude qui relaiera la demande auprès de la société MexBrain.

Le centre de coordination de l'étude, en lien avec MexBrain, est en charge d'assurer le bon approvisionnement du centre.

L'Unité des Dispositifs Médicaux Stériles du service de Pharmacie de l'Hôpital Lyon Sud ((Bâtiment 3A, Hôpital Lyon Sud – Pharmacien Responsable : Dr Cédric BESNIER) est responsable de la comptabilité des distributions des produits, des demandes de réapprovisionnement, de la vérification de la date de péremption et des modalités de conservation du produit.

11.4 Dispensation du dispositif

Le dispositif MEX-CD1 est dispensé par l'Unité des Dispositifs Médicaux Stériles du service de Pharmacie de l'Hôpital Lyon Sud (Bâtiment 3A, Hôpital Lyon Sud – Pharmacien Responsable : Dr Cédric BESNIER). Il n'y a pas de stock d'avance mis à disposition dans le service clinique.

CONFIDENTIEL

La dispensation est réalisée par la prescription de MEX-CD1 suite à l'inclusion d'un patient dans le cadre de l'étude MEXIRON. Lors de chaque dispensation (1 dispensation par séance d'EEC), 2 flacons sont dispensés.

11.5 Stockage

Avant son utilisation, le dispositif médical expérimental est stocké à température ambiante (15-25°C), dans un endroit sec, propre et sûr, au sein de l'Unité des Dispositifs Médicaux Stériles du Service de Pharmacie (Bâtiment 3A, Hôpital Lyon Sud – Pharmacien Responsable : Dr Cédric BESNIER).

Un système d'enregistrement de la température alerte en cas d'excursion. Ne pas conserver à la lumière directe du soleil ou à proximité de sources de chaleur. Ne pas congeler.

11.6 Contrôle de l'accès au dispositif médical expérimental

Seules les personnes impliquées dans l'étude MEXIRON et mentionnées dans la liste de délégation des tâches auront accès au dispositif médical expérimental.

11.7 Suivi de l'utilisation dans le cadre de l'investigation

Chaque séance d'Epuration ExtraCorporelle se fera sous la supervision de l'investigateur principal ou du coordinateur scientifique de l'IC, assisté d'une infirmière de recherche clinique présents durant toute la durée de la séance.

11.8 Retour des dispositifs médicaux expérimentaux inutilisés, arrivés à expiration ou présentant un dysfonctionnement

Les dispositifs médicaux expérimentaux inutilisés ou arrivés à péremption seront restitués à MexBrain (13 avenue Albert Einstein - 69100 Villeurbanne) en fin d'IC et remplacés en cours d'étude dans le cas où un dispositif médical expérimental MEX-CD1 présenterait un dysfonctionnement ou un défaut. Dans l'hypothèse peu probable où l'on assisterait à une rupture de membrane, la toxicité et l'hémocompatibilité du composé ont pu être testé et n'ont pas révélé de points d'inquiétude, démontrant même une élimination rénale rapide.

12 DECLARATION DE CONFORMITE

Le promoteur et l'investigateur s'engagent à ce que cette recherche soit conduite :

- Conformément au protocole ;
- Conformément aux bonnes pratiques cliniques françaises et internationales actuellement en vigueur ;
- Conformément aux dispositions législatives et réglementaires actuellement en vigueur en France ;
- Conformément au RDM 2017/745 ;
- Conformément à la norme ISO 9001, certification du promoteur depuis 2018.

Respectant toutes les normes citées ci-dessus, le promoteur ne revendique pas la conformité à la norme 14155.

13 ÉVALUATION DE LA SÉCURITÉ

13.1 Définitions de la vigilance des investigations cliniques

Selon l'article R1123-46 du code de la santé publique, le règlement européen sur le DM 2017/745, le MDCG 2020-10/1 et l'avis au Promoteur.

13.1.1 Evénement indésirable (EI)

Toute manifestation nocive, toute maladie ou blessure non intentionnelle ou tout signe clinique malencontreux, y compris un résultat de laboratoire anormal, chez des participants, des utilisateurs ou

CONFIDENTIEL

d'autres personnes, dans le cadre d'une investigation clinique, liée ou non au(x) dispositif(s) médical(caux) d'une investigation clinique.

Pour les utilisateurs ou autres personnes, cela est limité aux événements liés au dispositif médical expérimental.

Note :

a. Cette définition inclut les évènements attendus comme les inattendus.
b. Cette définition inclut les événements survenant dans le cadre d'une investigation clinique liés au dispositif expérimental, au comparateur ou aux procédures impliquées.

13.1.2 Effet indésirable du dispositif

Evènement indésirable relié à l'utilisation du DM expérimental ou au comparateur.

13.1.3 Evénement ou effet indésirable grave

Tout événement ou effet indésirable :

a) La mort ;

b) Une dégradation grave de l'état de santé du participant, laquelle est à l'origine :

i. d'une maladie ou blessure mettant en danger la vie du patient ;
ii. d'une déficience permanente d'une structure ou fonction anatomique ;
iii. d'une hospitalisation ou de la prolongation de l'hospitalisation du patient ;
iv. d'une intervention médicale ou chirurgicale visant à prévenir toute maladie ou blessure mettant en danger la vie du patient ou toute déficience permanente d'une structure ou fonction anatomique ;
v. d'une maladie chronique ;

c) Une souffrance foetale, la mort du foetus, des déficiences physiques ou mentales congénitales ou une malformation congénitale.

Le terme EIG inclut toute défectuosité d'un dispositif qui aurait pu déboucher sur un événement indésirable grave en l'absence de mesures appropriées ou d'une intervention ou si les circonstances avaient été moins favorables.

Se référer au paragraphe 13.2.6 pour les événements indésirables graves ne nécessitant pas une notification sans délai au promoteur et les évènements d'intérêt particulier.

13.1.4 Défectuosité du dispositif médical expérimental

Tout défaut en matière d'identité, de qualité, de durabilité, de fiabilité, de sécurité ou de performances du dispositif médical expérimental faisant l'objet d'une investigation, y compris tout dysfonctionnement, toute erreur d'utilisation ou tout défaut dans les informations fournies par le fabricant.

13.1.5 Classification des niveaux de causalité

La relation de causalité entre l'utilisation du dispositif médical expérimental (y compris l'intervention médico-chirurgicale) et la survenue de chaque événement indésirable doit être évaluée et classée.

A des fins d'harmonisation des déclarations, chaque EIG sera classée selon quatre niveaux de causalité différents :

1. **Non lié :** la relation de causalité avec le dispositif médical expérimental ou les procédures peut être exclue lorsque :
   - l'événement n'a pas de relation temporelle avec l'utilisation du dispositif médical expérimental ou les procédures liées à l'application du dispositif expérimental ;
   - l'événement indésirable grave ne suit pas un mécanisme d'action connu au dispositif médical expérimental (s'il est connu au préalable) et est biologiquement invraisemblable ;
   - l'arrêt de l'utilisation du dispositif médical ou la réduction du niveau d'activation/exposition lorsque cela est cliniquement possible et sa ré-utilisation (ou l'augmentation du niveau d'activation/exposition), n'ont pas d'impact sur l'événement indésirable grave ;

CONFIDENTIEL

- l'événement implique un endroit du corps ou un organe qui ne peut pas être affecté par le dispositif ou l'intervention ;
- l'événement indésirable grave peut être attribué à une autre cause (par exemple, une maladie/état clinique sous-jacent ou concomitant, un effet d'un autre dispositif, médicament, traitement ou d'autres facteurs de risque) ;
- l'événement ne dépend pas d'un faux résultat donné par le dispositif médical d'investigation utilisé pour le diagnostic, le cas échéant.

Afin d'établir l'absence de lien, tous les critères énumérés ci-dessus peuvent ne pas être remplis en même temps, selon le type de dispositif/de procédures et l'événement indésirable grave.

2. **Possible :** la relation de causalité avec l'utilisation du dispositif médical expérimental, ou la relation avec les procédures, est faible mais ne peut être totalement exclue. Des causes alternatives sont également possibles (par exemple, une maladie/état clinique sous-jacent ou concomitant ou/et un effet d'un autre appareil, médicament ou traitement). Les cas où le lien ne peut pas être évalué ou où aucune information n'a été obtenue doivent également être classés dans la mesure du possible.
3. **Probable :** la relation de causalité avec l'utilisation du dispositif médical expérimental, ou la relation avec les procédures, semble pertinente et/ou l'événement ne peut pas être raisonnablement expliqué par une autre cause.
4. Lien de causalité (**certain**)

Un événement indésirable qui est évalué avec une relation causale possible, probable ou certaine est considéré comme un effet.

13.1.6 Effet indésirable grave inattendu

Effet indésirable grave considéré comme inattendu si sa nature, son incidence, sa sévérité ou son évolution n'est pas identifié dans le document d'évaluation du risque en vigueur.

13.2 Responsabilité de l'investigateur

13.2.1 Modalités de détection et de recueil des événements indésirables

De la première visite (inclusion J_0) jusqu'à la fin de l'étude et jusqu'à leur résolution, l'investigateur recherche, rapporte, enregistre, traite et évalue dans l'eCRF :

a. **Tout événement indésirable** défini dans le protocole comme déterminant pour l'évaluation des résultats de l'investigation clinique ;
b. **Tout évènement indésirable grave** ;
c. **Toute défectuosité d'un DM** qui aurait pu déboucher sur un événement indésirable grave en l'absence de mesures appropriées ou d'une intervention, ou si les circonstances avaient été moins favorables ;
d. Tout nouvel élément concernant un événement visé aux points *a)* à *c)*.

Les événements suivants ne sont pas considérés comme des événements indésirables (ils ne sont donc pas à collecter dans le eCRF) :

- Admission pour raisons sociale ou administrative ;
- Hospitalisation prédéfinie par le protocole ;
- Passage en hôpital de jour programmé dans le cadre du suivi de la pathologie étudiée ou d'une maladie intercurrente déjà connue à l'inclusion.

L'intensité des événements indésirables sera déterminée de la façon suivante (CTCAE version 5.0) :

- **Légère** (grade 1) : symptomatique ou symptômes légers ; diagnostic à l'examen clinique uniquement ; ne nécessitant pas de traitement ;
- **Modérée** (grade 2) : nécessitant un traitement minimal, local ou non-invasif ; interférant avec les activités instrumentales de la vie quotidienne ;

CONFIDENTIEL

- **Sévère** ou médicalement significatif mais sans mise en jeu immédiate du pronostic vital (grade 3) : Indication d'hospitalisation ou de prolongation d'hospitalisation ; invalidant ; interférant avec les activités élémentaires de la vie quotidienne ;
- **Mise en jeu du pronostic vital** (grade 4) : nécessitant une prise en charge en urgence ;
- **Décès lié à l'évènement indésirable** (grade 5).

Tous les évènements indésirables doivent être gradés.

Tous les événements indésirables d'intensité sévère, menace du pronostic vital et décès (grade supérieur ou égal à 3) sont considérés comme GRAVES et doivent être notifiés sans délai au promoteur [sauf s'ils sont décrits dans le paragraphe 13.2.6 comme ne devant pas faire l'objet d'une notification sans délai auprès du promoteur].

Chaque grade d'événement indésirable doit être signalé séparément dans le eCRF (une ligne par grade si le grade change pour le même EI).

La date de fin d'un EIG due à une hospitalisation ou à une prolongation de l'hospitalisation ou à une mise en jeu du pronostic vital est la première date survenant entre la date de fin de l'hospitalisation ou la date du retour au grade précédent avant la survenue de l'EIG, sauf si l'investigateur juge qu'une autre date est cliniquement pertinente.

La date de fin d'un EIG due à un événement médicalement important est la date du retour au grade précédent.

Si un EI concerne des paramètres biologiques, la date de début et de fin doit être basée sur la date d'échantillonnage du sang et non sur la date du rapport de laboratoire.

13.2.2 Notification immédiate des Evénements Indésirables Graves (EIG) au promoteur

L'investigateur évalue chaque événement indésirable au regard de sa gravité.

L'investigateur doit notifier au promoteur, sans délai et au plus tard **dans les 3 jours calendaires** à compter du jour où il en a connaissance, tous les événements indésirables graves survenus dans l'essai et toute défectuosité d'un dispositif qui aurait pu déboucher sur un événement indésirable grave en l'absence de mesures appropriées ou d'une intervention ou si les circonstances avaient été moins favorables, à l'exception de ceux qui sont recensés dans le protocole ou dans la brochure pour l'investigateur comme ne nécessitant pas une notification sans délai (paragraphe 13.2.6).

Cette notification initiale fait l'objet d'un rapport et doit être suivie par un ou des rapport(s) complémentaire(s) détaillé(s) dans les 8 jours suivant la première notification.

L'investigateur valide le formulaire de notification d'EIG le date, et l'envoie par mail via l'eCRF à drci.eig-vigilance@chu-lyon.fr, dès lors qu'il a les 4 éléments minimum pour notifier un EIG :

- Un notificateur,
- Un sujet,
- Un dispositif expérimental (si applicable),
- Un événement indésirable grave.

L'investigateur doit documenter au mieux l'événement (grâce aux copies des résultats de laboratoires ou des comptes rendus d'examens ou d'hospitalisation renseignant l'événement grave, y compris les résultats négatifs pertinents, **sans omettre de rendre ces documents anonymes** et d'inscrire le numéro et le code du patient), le diagnostic médical et établir **un lien de causalité** entre l'événement indésirable grave et le(s) dispositif(s) médical(aux) et le geste de mise en oeuvre.

L'investigateur doit suivre le patient ayant présenté un EIG jusqu'à sa résolution, une stabilisation à un niveau jugé acceptable par l'investigateur ou le retour à l'état antérieur, même si le patient est sorti de l'investigation et informer le promoteur en complétant l'eCRF, puis en envoyant par mail à drci.eig-vigilance@chu-lyon.fr (remplir la partie FU [Follow Up] avec nom, date et signature sur l'eCRF).

Si l'eCRF est indisponible, l'investigateur peut scanner le formulaire « papier » de notification d'EIG signé et daté et l'envoyer par mail à drci.eig-vigilance@chu-lyon.fr. Il doit préciser dans l'objet du mail « MEXIRON-Critère de gravité*-N°centre-patient ».

*à remplir fonction du critère de gravité sélectionné dans le formulaire de notification d'EIG.

13.2.3 Evaluation de la causalité des EI

L'investigateur doit évaluer, distinguer et hiérarchiser le lien de causalité des événements indésirables avec le dispositif médical expérimental faisant l'objet de l'IC et/ou la procédure d'investigation. La présence de facteurs confondants, tels que les traitements concomitants, les antécédents médicaux ou autres facteurs de risques doivent être considérés.

Chaque EIG sera évalué selon 4 niveaux de causalité : non relié, possible, probable ou certain.

L'évaluation de la causalité doit être effectuée distinctement pour les procédures d'investigation.

13.2.4 Période de notification des EIG relié à la procédure d'investigation

L'investigateur doit notifier sans délai au promoteur les évènements indésirables graves :

- **Dès l'inclusion du patient** (date de signature du 1er consentement)
- Jusqu'à **la fin de la participation du patient dans l'étude**
- Sans limite de durée pour les évènements indésirables graves reliés à la recherche (par exemple : cancers, malformations congénitales survenant à long terme après exposition au médicament expérimental…).

13.2.5 Evènement d'intérêt particulier

Certains événements nécessitent d'être surveillés particulièrement et seront notifiés comme un EI grave même s'ils ne répondent pas aux critères de gravité ou si l'intensité est < Grade 3 parmi lesquels :

- Défectuosité d'un dispositif causant un EIG ;
- Défectuosité d'un dispositif qui aurait pu déboucher sur un événement indésirable grave en l'absence de mesures appropriées ;
- Réaction anaphylactique, manifestations de type allergiques quel que soit le grade pendant les séances ;
- Réactions fébriles supérieure à 38,5°C et chocs pyrogènes pendant les séances;
- Hémolyse intravasculaire ;
- Allo-immunisation ;
- Événements hémorragiques dans les 48h suivant la séance d'Epuration ExtraCorporelle et nécessitant une transfusion ;
- Changement de grades CTCAE des cytopénies, plus particulièrement de l'anémie ;
- Troubles du rythme cardiaque ;
- Infections nécessitant une antibiothérapie oral et/ou IV ;
- Hypotension artérielle
- Colite ischémique.

13.2.6 Restriction des EIG (formulaire de notification des EIG à ne pas envoyer au secteur vigilance mais à recueillir uniquement dans l'eCRF au plus tard à la fin de participation à l'étude) :

- Hospitalisation pour traitement médical ou chirurgical programmé avant la recherche ;
- Admission aux urgences de moins de 24 heures (non liée au traitement) ;
- Neutropénie fébrile (grade 3) ;
- Transfusions liées au soin courant et à la prise en charge du patient habituelle.

13.3 Responsabilités du promoteur

13.3.1 Déclaration aux autorités compétentes

Conformément à l'article R1123-55 du CSP, au règlement européen sur le DM 2017/745 et au MDCG 2020-10/1, le promoteur doit déclarer aux autorités compétentes les « événements reportables » suivants :

a. **Tout événement indésirable grave** entretenant avec le dispositif faisant l'objet de

l'investigation, le dispositif comparateur ou la procédure d'investigation qui le précède, **un lien de causalité avéré** ou raisonnablement envisageable;

b. **Toute défectuosité d'un dispositif qui aurait pu déboucher sur un événement indésirable grave** en l'absence de mesures appropriées ou d'une intervention, ou si les circonstances avaient été moins favorables,

c. Tout nouvel élément concernant un événement visé aux points a) et b).

Seul le niveau de causalité 1 (c'est-à-dire « non lié ») est exclu de la déclaration.

Délais de déclaration	Type d'événements « reportables »
**Sans délai** (immédiatement), et pas plus tard que **2 jours** calendaires à compter du jour où le promoteur a eu connaissance de l'évènement à déclarer ou d'une nouvelle information<br>concernant un événement déjà déclaré.	**Tous les événements ayant entraîné la mort ou un risque de mort imminente, une blessure ou maladie grave** et qui requièrent une action corrective rapide pour les participants/patients, utilisateurs ou d'autres personnes, ou toute nouvelle information sur ces cas.<br>*Cela inclut des événements pertinents et inattendus lorsqu'ils deviennent un danger potentiel pour la santé publique. Cela inclut également la possibilité de décès multiples survenant à de courts intervalles.*
**Sans délai** (immédiatement), et pas plus tard que **7 jours** calendaires à compter du jour où le promoteur a eu connaissance de l'évènement à déclarer ou d'une nouvelle information<br>concernant un événement déjà déclaré.	**Les autres évènements « reportables »** ou toute nouvelle information/actualisation les concernant.

13.3.2 Documents d'évaluation du risque pour l'évaluation du caractère attendu/inattendu

13.3.2.1 Risques liés au dispositif médical expérimental

Dispositif médical expérimental n°1 : MEX-CD1 (MexBrain).
Document de référence : paragraphe 4.2 « Adverse Device effects » de la Brochure Investigateur.

13.3.2.2 Risques liés aux procédures d'investigation (pour la mise en oeuvre du dispositif médical expérimental)

Les risques potentiels liés aux séances d'Epuration ExtraCorporelle sont :

- Douleur au point(s) de ponction veineuse (nécessitant des antalgiques additionnels) ;
- Hématome au(x) point(s) de ponction(s) veineuse(s) (avec retentissement fonctionnel et/ou perte sanguine significative) ;
- Hypotension pendant la(les) séance(s) d'Epuration Extra Corporelle (diminution PAS >20 mmHg ou PAM >10mmHg par rapport à la PA pré épuration (N.B = définition de l'hypoTA perdialyse)) ;
- Malaises, frissons, inconfort ressenti par le patient ;
- Veinite au(x) point(s) de ponction(s) veineuse(s) ;
- Thrombose veineuse superficielle / profonde aux membre supérieurs ;
- Nausées, vomissements ;
- Douleurs thoracique/dyspnée ;
- Céphalées ;
- Embolies gazeuses ;
- Septicémie ;
- Crampes/spasmes musculaires ;
- Syncopes, convulsions ;
- Fièvre ;
- Fatigue, asthénie ;
- Hypertension ;
- Hypotension ;
- Syndrome de déséquilibre : céphalées, photophobie, nausées ;

- Troubles digestifs ;
- Hypercalcémie, alcalose métabolique ;
- Hémorragies digestives non sévères ;
- Troubles du rythme cardiaque ;
- Angor, infarctus du myocarde ;
- Manifestation de type allergique : gêne respiratoire à composante bronchospasmodique, toux quinteuse, écoulement nasal, conjonctivite, impression de chaleur généralisée, érythème cutané prurigineux avec parfois chute de la tension artérielle, réaction anaphylactique ou anaphylactoïde ;
- Hémolyse intravasculaire (augmentation de la bilirubine) ;
- Infection ;
- Colite ischémique.

13.3.2.3 Description des effets indésirables reliés aux autres procédures additionnelles

Les effets indésirables attendus pouvant être reliés aux prélèvements pour réaliser les bilans biologiques sont :

- Douleur au point(s) de ponction veineuse ;
- Hématome au(x) point(s) de ponction(s) veineuse(s) ;
- Hémorragie ;
- Asthénie.

Les effets indésirables attendus pouvant être reliés à l'anticoagulation préventive (ENOXAPARINE 2000 UI) sont : se référer à la partie 4.8 du RCP du LOVENOX (https://base-donnees-publique.medicaments.gouv.fr/affichageDoc.php?specid=60755562&typedoc=R)

13.3.2.4 Description des événements indésirables reliés à la maladie étudiée

Ces événements indésirables seront considérés comme reliés à la maladie étudiée et non au dispositif médical expérimental faisant l'objet de l'investigation clinique, ni à la procédure d'investigation.

- Progression ou rechute de l'hémopathie, hémoglobinopathie ou de la pathologie d'origine immuno-hématologique sous-jacente
- Rechute pré-allogreffe des patients suivis pour une leucémie aigue en attente d'allogreffe
- Acutisation en Leucémie Aiguë Myéloïde (LAM) pour les patients suivis pour un syndrome myélodysplasique, myéloprolifératif ou une aplasie médullaire idiopathique;
- Dégradation des cytopénies (anémie, thrombopénie, neutropénie) ;
- Apparition de cytopénies (anémie, thrombopénie, neutropénie) ;
- Rupture splénique (myélofibrose primitive) ;
- Hémorragie ;
- Asthénie ;
- Complications infectieuses (pneumonie, septicémie).

13.3.2.5 Description des événements indésirables reliés au soin

Ces événements indésirables seront considérés comme reliés au soin courant et à la prise en charge médicale habituelle pour ces patients :

- Complications liées aux transfusions (i.e. oedèmes aigus pulmonaires, hémochromatose secondaire).

13.3.2.6 Description des événements indésirables à ne pas déclarer à l'ANSM

- Les complications dues aux traitements concomitants, qui ne sont donc pas imposés par le protocole de l'IC, ne peuvent être considérées comme des événements reliés au DM, au comparateur ou à la procédure d'investigation. Ils doivent être transmis au centre régional de pharmacovigilance.
- Les complications liées aux procédures de routine (diagnostiques ou thérapeutiques) mises en œuvre dans le cadre de la **prise en charge habituelle** du patient en dehors du protocole d'IC.

CONFIDENTIEL

13.4 Comité de surveillance indépendant

Le Comité de Surveillance Indépendant (CSI) est un comité consultatif chargé de donner au Promoteur d'un essai clinique, un avis sur la conduite de l'essai. A cet effet, il examine l'ensemble des problèmes pouvant apparaître dans l'essai, notamment scientifiques, éthiques et de tolérance, susceptibles de modifier le rapport bénéfice/risque. Suite à cet examen, il transmet par écrit ses recommandations au Promoteur. Ces recommandations peuvent concerner notamment la poursuite, la modification ou l'arrêt de l'essai.

Le Promoteur reste décisionnaire des mesures à mettre en œuvre, suite aux recommandations du CSI.

Les modalités de fonctionnement de ce CSI sont décrites dans une charte signée par les membres du CSI en début d'étude.

Il est composé de trois membres dont un expert clinicien des pathologies étudiées, un expert de la procédure étudiée et un méthodologiste (cf. 1.12).

Dans le cadre de cette IC, le CSI sera sollicité après la visite de suivi V1 du premier patient inclus puis tous les 3 patients inclus après leur suivi V_2 avec les informations de sécurité pertinentes (décès, EIG, EIP). Les inclusions seront suspendues uniquement pour le premier patient le temps que le CSI rende ses recommandations sur la base des informations de sécurité (décès, EIG, EIP) et sur les conditions de déroulement de l'essai qui lui aura été communiquées par e-mail.

14 SUIVI DES PARTICIPANTS APRES LA FIN DE L'IC

14.1 Arrêt de participation d'une personne à la recherche

Les sujets peuvent retirer leur consentement et demander à sortir de l'étude à n'importe quel moment et quelle qu'en soit la raison. En cas de sortie prématurée, l'investigateur doit en documenter les raisons de façon aussi complète que possible.

L'investigateur peut interrompre temporairement ou définitivement la participation d'un sujet à l'étude pour toute raison qui servirait au mieux les intérêts du sujet en particulier en cas d'événements indésirables graves.

La mise en évidence d'un ou plusieurs critères de sortie d'étude tels que définis précédemment peut également amener l'investigateur à arrêter la participation du sujet à la recherche.

En cas de sujet perdu de vue, l'investigateur met tout en œuvre pour reprendre contact avec la personne pour recueillir si possible la raison de la perte de vue.

En cas de retrait de consentement, les données recueillies jusqu'à la date du retrait sont analysées.

Si la sortie d'étude s'effectue avant que le sujet ait pu bénéficier d'un cycle complet de 3 séances d'Epuration ExtraCorporelle consécutives, celui-ci sera remplacé.

14.2 Arrêt d'une partie ou de la totalité de la recherche

L'étude peut être interrompue prématurément en cas de survenue d'événements indésirables inattendus, graves nécessitant une revue du profil de la stratégie. Ainsi, tout effet indésirable grave conduisant à une hospitalisation non programmée (par exemple, réaction anaphylactique ou anaphylactoide, hyperthermie prolongée) conduira à la programmation d'une réunion systématique du CSI qui décidera de la poursuite de l'essai. De même, des événements imprévus, les recommandations du CSI ou de nouvelles informations relatives à la méthode d'investigation, au vu desquels les objectifs de l'étude ne seraient vraisemblablement pas atteints, pourraient amener le promoteur à interrompre prématurément l'IC.

En tant que promoteur de l'IC, les Hospices Civils de Lyon se réservent le droit d'interrompre l'étude à tout moment, s'il s'avère que les objectifs d'inclusion ne sont pas atteints.

En cas d'arrêt prématuré de l'étude pour des raisons de sécurité, l'information sera transmise par le promoteur sans délai à l'ANSM et au CPP.

CONFIDENTIEL

15 PRISE EN CHARGE DES PARTICIPANTS

Après la fin de l'IC, les patients sont pris en charge selon les modalités habituelles. Aucun soin supplémentaire n'est nécessaire du fait de leur participation.

16 RAPPORT D'INVESTIGATION CLINIQUE

Les communications et rapports scientifiques correspondant à cette étude seront réalisés sous la responsabilité de l'investigateur principal de l'investigation clinique avec l'accord des investigateurs associés. Les coauteurs du rapport et des publications seront les investigateurs et les cliniciens impliqués, au prorata de leur contribution à l'étude, ainsi que les méthodologistes, le biostatisticien et les chercheurs associés.

Les règles de publications suivront les recommandations internationales (N Engl J Med, 1997; 336 :309-315).

L'étude sera enregistrée sur un registre des essais cliniques en libre accès (clinicaltrials.gov) avant l'inclusion du premier patient.

17 REFERENCES BIBLIOGRAPHIQUES*

1. Coates, T.D., *Physiology and pathophysiology of iron in hemoglobin-associated diseases.* Free Radic Biol Med, 2014. **72**: p. 23-40.
2. Hebbel, R.P., *Auto-oxidation and a membrane-associated 'Fenton reagent': a possible explanation for development of membrane lesions in sickle erythrocytes.* Clin Haematol, 1985. **14**(1): p. 129-40.
3. Le Lan, C., et al., *Redox active plasma iron in C282Y/C282Y hemochromatosis.* Blood, 2005. **105**(11): p. 4527-31.
4. Fung, E.B., et al., *Morbidity and mortality in chronically transfused subjects with thalassemia and sickle cell disease: A report from the multi-center study of iron overload.* Am J Hematol, 2007. **82**(4): p. 255-65.
5. Westhofen, G., et al., *Comprehensive Genomic Analysis Provides Further Evidence That Iron Overload Can Induce Genetic Instability in Myelodysplastic Syndromes* Blood, 2015. **126**(23): p. 2842.
6. Westhofen, G., et al., *Comprehensive Genomic Analysis Provides Further Evidence That Iron Overload Can Induce Genetic Instability in Myelodysplastic Syndromes.* Blood, 2015. **126**(23).
7. Cohen, A.R., E. Glimm, and J.B. Porter, *Effect of transfusional iron intake on response to chelation therapy in beta-thalassemia major.* Blood, 2008. **111**(2): p. 583-7.
8. Taher, A., et al., *Improved treatment satisfaction and convenience with deferasirox in iron-overloaded patients with beta-Thalassemia: Results from the ESCALATOR Trial.* Acta Haematol, 2010. **123**(4): p. 220-5.
9. Hoffbrand, A.V., A. Taher, and M.D. Cappellini, *How I treat transfusional iron overload.* Blood, 2012. **120**(18): p. 3657-69.
10. Di Maggio, R. and A. Maggio, *The new era of chelation treatments: effectiveness and safety of 10 different regimens for controlling iron overloading in thalassaemia major.* Br J Haematol, 2017. **178**(5): p. 676-688.
11. Lai, M.E., et al., *Increased survival and reversion of iron-induced cardiac disease in patients with thalassemia major receiving intensive combined chelation therapy as compared to desferoxamine alone. .* Blood Cells Mol Dis., 2010. **45**(2): p. 136-9.
12. Lai, M.E., et al., *Increased survival and reversion of iron-induced cardiac disease in patients with thalassemia major receiving intensive combined chelation therapy as compared to desferoxamine alone.* Blood Cells Mol Dis, 2010. **45**(2): p. 136-9.
13. Shammo, J.M. and R.S. Komrokji, *Clinical consequences of iron overload in patients with myelodysplastic syndromes: the case for iron chelation therapy.* Expert Rev Hematol, 2018. **11**(7): p. 577-586.

CONFIDENTIEL

14. Armand, P., et al., *Iron overload in patients with acute leukemia or MDS undergoing myeloablative stem cell transplantation.* Biol Blood Marrow Transplant, 2011. **17**(6): p. 852-60.
15. Malcovati, L., et al., *Prognostic factors and life expectancy in myelodysplastic syndromes classified according to WHO criteria: a basis for clinical decision making.* J Clin Oncol, 2005. **23**(30): p. 7594-603.
16. Sanz, G., et al., *Independent Impact of Iron Overload and Transfusion Dependency on Survival and Leukemic Evolution in Patients with Myelodysplastic Syndrome.* Blood, 2008. **112**(11).
17. Schafer, A.I., et al., *Clinical consequences of acquired transfusional iron overload in adults.* N Engl J Med, 1981. **304**(6): p. 319-24.
18. Takatoku, M., et al., *Retrospective nationwide survey of Japanese patients with transfusion-dependent MDS and aplastic anemia highlights the negative impact of iron overload on morbidity/mortality.* Eur J Haematol, 2007. **78**(6): p. 487-94.

CONFIDENTIEL

18 LISTE DES ANNEXES

Annexe 1 : Brochure Investigateur MEX-CD1 (R-DND-12 IB MEX-CD1)

Annexe 2 : Auto-questionnaire MOS SF-12
Référence : Gandek B et al. J Clin Epidemiol. 1998 Nov;51(11):1171-8.

CONFIDENTIEL
