# Supplementary material for: Protocol for the MEXIRON study: a first-in-disease phase I–II investigation into the safety, tolerability and mechanistic proof-of-concept of extracorporeal iron depletion using the MEX-CD1 device in patients with transfusional iron overload
Source: BMJ Open. 2026 Oct 27;16(10):e121216. doi: 10.1136/bmjopen-2026-121216 (PMC13723837; doi:10.1136/bmjopen-2026-121216)
Supplement: Supplementary file 2 [file bmjopen-2026-121216.reviewer_comments.pdf]

PEER REVIEW HISTORY

BMJ Open publishes all reviews undertaken for accepted manuscripts. Reviewers are asked to complete a checklist review form and are provided with free text boxes to elaborate on their assessment. These free text comments are reproduced below.

ARTICLE DETAILS

Title (Provisional)

Protocol for the MEXIRON Study: A First-in-Disease Phase I–II Investigation into the Safety, Tolerability, and Mechanistic Proof-of-Concept of Extracorporeal Iron Depletion Using the MEX-CD1 Device in Patients with Transfusional Iron Overload.

Authors

Dargent, Auguste; Heiblig, Mael; VILLENEUVE, LAURENT; Alves Ferreira, Marine; Bonjour, Maxime; Lux, Francois; Tillement, Olivier; Allaouchiche, Bernard

---

VERSION 1 - REVIEW

**Reviewer**	**1**
**Name**	**Słomka, Artur**
**Affiliation**	**Nicolaus Copernicus University, Collegium Medicum, Department of Pathophysiology**
**Date**	**13-Jul-2026**
**COI**	**None**

This is an interesting and well-prepared study protocol addressing an important clinical problem. The proposed extracorporeal approach is innovative, and the safety-oriented proof-of-concept design is appropriate. The protocol is generally clear, but several points require clarification before publication.

1. The Introduction would benefit from a more precise discussion of iron regulation in MDS and the biological significance of labile iron. The authors should consider citing the following relevant publications: DOI: 10.3390/cancers16020332 and DOI: 10.3389/fphar.2014.00045.

2. The terms NTBI, LPI, free iron, and labile iron should be clearly defined and used consistently. Total iron measured in protein-free effluent should not automatically be described as LPI without appropriate analytical validation.

3. The analytical procedure for the primary endpoint requires more detail. Please report calibration, limits of detection and quantification, recovery, reproducibility, blank measurements, contamination control, and correction for polymer or membrane adsorption.

4. The analysis populations should be clearly defined. The protocol refers to 10 evaluable patients, 13 recruited patients, and an intention-to-treat analysis. Please specify the safety, full-analysis, and evaluable populations and explain how incomplete sessions will be handled.

5. The proposed mixed-effects models may be too complex for only 10 evaluable participants. The authors should justify the random-effects structure and provide a simplified analytical approach if the models do not converge. The analysis should remain primarily descriptive.

6. The inclusion of both MDS and myelofibrosis should be justified. These populations may differ in disease biology, transfusion burden, and iron metabolism. Results should be reported separately where possible. The exclusion of patients receiving azacitidine, luspatercept, or erythropoietin should also be explained.

7. Several protocol details should be harmonised. These include "first-in-man" versus "first-in-disease," intermittent haemodialysis versus CVVHD, a session duration of 200 minutes versus 4 hours, and the platelet threshold of ≥50 G/L versus mandatory transfusion at ≤50 G/L. Recruitment dates, current study status, and prespecified stopping criteria should also be provided.

**Reviewer**	**2**
**Name**	**Arat, Mutlu**
**Affiliation**	**Grup Florence Nigtingale Hospital Turkey, Turkey**
**Date**	**28-Jul-2026**
**COI**	**None**

I congratulate the authors for this novel proposal and study. Having a tool for immediate labile iron removal in any transfusion dependent hematological entitiy is a dream, esp transplant physicians. I share my comments below.

Major:

1- P8L28 WHy not only MDS for more focus and homogenity and including MF as well. Please clarify

2- P8L39 You are planning to have patients accoriidng to liver concentrations. Having hifh Ferritin levels and high liver concentrations are 2 different scenarios. End organ involvement is and end effect. You should define exactly your very limited 10 patient pool. You have to slect etiher >10 transfusion already and high Ferritin but no liver accumulation, or >20 transfusions and lver iron concenration high. The inclsuion criteria should be accurate and well defined, you are focusing more to mechanistics and efficiency, but clinicans will be demanding on the efficnecy in ceratin patient population

3- COncomitatnt teratment excluded, please define: At the time of the procedure week, acceptable, but excluding all the patients on Rx will be not wise and fair.

4- Why allo Tx reciepeint candidates excluded? Any specific reason, these are the patients in urgent need.

5- Share your expactation for iron removal for each session and add an proposed analysis for a standart candiate with a certain Ferritin and labile iron.

Minor:

1- P8L42 define chelation failure by refrence, exact time ans agent use

2- P8L53 Capacity, needs rewording

3- P8L48, ... use not G but metrics, 10e9/L

4- Why sessions are 200 mins, please xplain

5- Why a dense program like HeD sessions, not every week. Any sicientific or regularoty or ethical raason, please clarify.

**** The reviewer provided a marked copy with additional comments. Please contact the publisher for full details. ****

VERSION 1 - AUTHOR RESPONSE

Reviewer 1 — Dr Artur Słomka

We thank Reviewer 1 for a rigorous and technically precise review. The comments on terminology, analytical validation and the analysis populations identified real weaknesses in the submitted version, and addressing them has materially improved the protocol.

> ***1.*** *The Introduction would benefit from a more precise discussion of iron regulation in MDS and the biological significance of labile iron. The authors should consider citing the following relevant publications: DOI: 10.3390/cancers16020332 and DOI: 10.3389/fphar.2014.00045.*

Response. We agree and have added two paragraphs to the Background. The first addresses iron regulation specifically in myelodysplastic syndromes: ineffective erythropoiesis drives erythroferrone and GDF-15, which suppress hepatic hepcidin transcription, so that intestinal absorption and macrophage iron release continue despite an expanded iron pool. We note, following the systematic review by Słomka et al., that reported hepcidin concentrations in MDS are heterogeneous and vary with WHO subtype, transfusion history and molecular background, notably SF3B1 status, which limits hepcidin's value as a stand-alone biomarker but establishes the central point for this protocol: the transfused patient accumulates iron by an endogenous as well as an exogenous route, which is why iron overload in MDS develops within months and is not reversed by interrupting transfusion. The second paragraph addresses the biological significance of labile iron and is described under comment 2. Both suggested references have been cited.

> **Change in the manuscript.** Background, two new paragraphs following the opening paragraph.

__2.__ The terms NTBI, LPI, free iron, and labile iron should be clearly defined and used consistently. Total iron measured in protein-free effluent should not automatically be described as LPI without appropriate analytical validation.

Response. This is an important comment, and we accept it without reservation. The submitted manuscript used 'free iron', 'labile iron' and 'LPI' interchangeably, and — as the reviewer says — it described total iron in a protein-free effluent as labile iron on the basis of an argument about membrane cut-off that establishes only that the iron was not protein-bound. Two changes have been made.

First, a new Background paragraph defines the terms explicitly.

Second, the primary outcome has been renamed and its interpretation bounded. It is now reported throughout as dialysable NTBI. The Rationale subsection states the limit of the inference explicitly: total iron measured in a protein-free effluent establishes that the iron was dialysable and therefore non-transferrin-bound, but it does not by itself establish that it was redox-active, and it is therefore not equivalent to LPI as measured by a validated chelator-based fluorimetric assay. The protocol now states in terms that no claim that the device specifically removes the redox-active subset of NTBI will be made on the basis of this measurement.

Change in the manuscript. Background (new terminology paragraph); Primary outcome; Rationale for outcome selection and analytical considerations; Abstract; Strengths and limitations.

__3.__ The analytical procedure for the primary endpoint requires more detail. Please report calibration, limits of detection and quantification, recovery, reproducibility, blank measurements, contamination control, and correction for polymer or membrane adsorption.

Response. A new subsection, 'Analytical methods for iron quantification', has been added.

Change in the manuscript. New subsection 'Analytical methods for iron quantification' in Methods and analysis; two of the Strengths and limitations bullets now reflect these points.

__4.__ The analysis populations should be clearly defined. The protocol refers to 10 evaluable patients, 13 recruited patients, and an intention-to-treat analysis. Please specify the safety, full-analysis, and evaluable populations and explain how incomplete sessions will be handled.

Response. The reviewer is right that the submitted version was unclear. Reviewing this point also brought to light an error in the submitted manuscript, which we correct here: the parenthesis defining evaluable patients as 'patients who complete the full cycle of 3 sessions' did not reflect the protocol. Evaluability requires the completion of at least one full dialysis session. A new subsection, 'Analysis populations', now defines two populations and the relationship between the figures of 13 and 10:

Safety population: all included patients in whom the procedure was initiated, that is, in whom cannulation was attempted, irrespective of whether a session was subsequently delivered; analysed as treated; the population for all adverse events, AESI, vital signs and biological safety data.

Evaluable population: patients who completed at least one full dialysis session, that is, a session delivered to its programmed 200-minute term; the population for the analysis of iron extraction, each patient contributing the sessions actually completed. This is what the target of 10 refers to.

Change in the manuscript. New subsection 'Analysis populations'; Statistical power and sample size; Data collection.

5. The proposed mixed-effects models may be too complex for only 10 evaluable participants. The authors should justify the random-effects structure and provide a simplified analytical approach if the models do not converge. The analysis should remain primarily descriptive.

Response. We acknowledge that the random-effects structure initially proposed may be over-parameterized for the sample size of this pilot study, and we have revised the statistical analysis section accordingly.

We clarified that the analysis of this pilot trial is primarily descriptive. Individual and aggregate trajectories of free iron, ferritin, and TSC will be presented graphically, together with summary statistics at each time point. Any modelling is secondary and exploratory, intended to estimate the magnitude and precision of the effect for the design of a subsequent and adequately powered trial, not to provide confirmatory hypothesis testing.

We simplified and justified the random-effects structure:

- Session is retained as a fixed effect with three levels (the session-level variance has been removed)
- The random effect structure is therefore limited to a random intercept per patient, accounting for between-patient heterogeneity in baseline biomarker parameters and for the correlation between repeated measurements within the same patient.
- A random intercept for session nested within patient will be considered only for the primary endpoint and only if the model converges.
- A random slope for time will not be fitted.

All decisions regarding model reduction will be documented in the statistical analysis plan and reported transparently in the final publication.

Change in the manuscript. Statistical analysis plan, rewritten.

6. The inclusion of both MDS and myelofibrosis should be justified. These populations may differ in disease biology, transfusion burden, and iron metabolism. Results should be reported separately where possible. The exclusion of patients receiving azacitidine, luspatercept, or erythropoietin should also be explained.

Response. We are grateful for this comment, and we must be transparent about how the protocol has evolved, because the direction it has taken is the opposite of the one the reviewer's comment might suggest.

Reviewer 2 raised the same criterion from the other side, proposing that eligibility be extended to patients with an indication for allogeneic transplantation. Independently of this review, and in the light of the first months of screening, the study scientific committee reconsidered the target population and decided to broaden rather than to narrow it. Eligibility is now defined by the physiological state under investigation — documented transfusional iron overload — rather than by the underlying diagnosis, which may be a haematological malignancy, a haemoglobinopathy, or another condition of immuno-haematological origin.

We set out the reasoning candidly, because we recognise it runs against the reviewer's instinct.

The mechanism under investigation is not disease-specific. The device removes low-molecular-weight, NTBI from plasma by diffusion into a chelating dialysate. What determines the quantity available for removal is transferrin saturation and the size of the circulating NTBI pool, not the marrow disorder that generated the transfusion requirement.

The primary outcome is a physicochemical quantity measured in the effluent. There is no biological rationale for expecting it to differ between a myelodysplastic syndrome and, say, a transfusion-dependent thalassaemia at equivalent transferrin saturation. Restricting the study to a single diagnosis would therefore not have made the primary outcome more homogeneous — which is the concern the reviewer is raising — while it would have imposed a real cost.

That cost is recruitment. Patients who are simultaneously transfusion-dependent, iron-overloaded, intolerant of or refractory to chelation, above 50 kg, above 50 × 10^9/L platelets and endowed with adequate peripheral venous access constitute a very small pool in a single centre. Restriction by diagnosis was, in practice, the binding constraint on completing a ten-patient mechanistic study.

We nevertheless accept the substance of the concern about heterogeneity, and we address it analytically rather than by restriction. With ten evaluable patients, no inferential comparison between diagnostic subgroups is planned, and none would be interpretable; we say so in the manuscript rather than promising subgroup analyses that the sample size cannot support. What we do commit to is full individual patient-level reporting: each participant's diagnosis and iron-status descriptors will be presented alongside their extraction data, so that any disease-related pattern is directly visible to the reader. We believe this satisfies the reviewer's request that results be reported separately where possible, in the only form that ten patients permit.

On concomitant treatments: azacitidine and other repeated-schedule cytotoxic agents alter erythropoiesis, transfusion requirement and iron kinetics over precisely the time frame of the intervention and its 90-day follow-up, and with ten evaluable patients that variability could not be distinguished from the effect of the device. Luspatercept and erythropoiesis-stimulating agents remain excluded on the same ground, both acting directly on erythropoiesis and on the transfusion requirement; patients on stable background JAK1/2 inhibition are, by contrast, eligible.

> **Change in the manuscript.** Title and Abstract (population broadened); Eligibility criteria (inclusion and exclusion rewritten); new subsection 'Rationale for the eligibility criteria'.

> ***7.** Several protocol details should be harmonised. These include 'first-in-man' versus 'first-in-disease', intermittent haemodialysis versus CVVHD, a session duration of 200 minutes versus 4 hours, and the platelet threshold of ≥50 G/L versus mandatory transfusion at ≤50 G/L. Recruitment dates, current study status, and prespecified stopping criteria should also be provided.*

Response. All of these have been corrected. Taking them in turn:

First-in-man versus first-in-disease. 'First-in-disease' is the accurate term and is now used throughout; The Aim of the study now states plainly that MEX-CD1 has previously been administered to humans in other indications, including Wilson's disease (MEXWILS, NCT05917327, publication pending), so that this is not strictly a first-in-human study of the device but the first use of the approach in transfusional iron overload.

Intermittent haemodialysis versus CVVHD. The modality is CVVHD: the new 'Rationale for the dialysis prescription' subsection states that a CRRT platform was chosen because it can deliver the low, precisely controlled dialysate flow (600 mL/h) required to work with a fixed 2 L volume of chelating dialysate, which a conventional haemodialysis monitor designed for 300–800 mL/min cannot do. The term 'intermittent haemodialysis' has been removed.

200 minutes versus 4 hours. 200 minutes is correct, and the '4 hours' in the feasibility outcome was an approximation, now corrected. The manuscript now explains that 200 minutes is 2,000 mL ÷ 600 mL/h, the time required to deliver the entire prepared volume of chelating dialysate exactly once in single-pass mode.

Platelet threshold: we corrected the typo of the “below or equal sign”. Transfusion is mandated if platelet count is $< 50 \times 10^9$/L (not ≤)

Recruitment dates, status and stopping criteria. New subsections, 'Trial status' and 'stopping rules', have been added.

Units. All platelet counts are now expressed in $\times 10^9$/L rather than Giga/L (see also Reviewer 2, minor comment 3).

Change in the manuscript. Title; Aim of the study; Study design; Interventions; new subsections 'Rationale for the dialysis prescription' and 'Trial status and stopping rules'; Feasibility outcomes.

Reviewer 2 — Dr Mutlu Arat

We thank Reviewer 2 for the encouraging assessment and for accurate comments. Several of them have led to substantive changes.

Major comments

> ***Major 1.*** *P8L28 Why not only MDS for more focus and homogeneity, and including MF as well? Please clarify.*

Response. The study scientific committee has in fact taken the opposite direction and broadened eligibility to transfusion-dependent patients with a haematological malignancy or another condition of immuno-haematological origin. The full reasoning is set out under Reviewer 1, comment 6, and in the new 'Rationale for the eligibility criteria' subsection: in brief, the mechanism and the primary outcome are physicochemical and not disease-specific, so restriction by diagnosis would not have improved homogeneity of the primary outcome, while it was the binding constraint on recruitment. Heterogeneity is handled by full individual patient-level reporting of diagnosis and iron-status descriptors alongside extraction data, rather than by restriction or by underpowered subgroup analyses.

> ***Major 2.*** *P8L39 You are planning to have patients according to liver concentrations. Having high ferritin levels and high liver concentrations are 2 different scenarios… The inclusion criteria should be accurate and well defined.*

Response. We agree that ferritin and liver iron concentration describe different scenarios and that the submitted criterion left this unaddressed. Two changes have been made.

First, the ferritin criterion has been made more robust: serum ferritin > 1,000 µg/L is now required on two successive measurements at least two weeks apart, rather than on a single value.

Second, we state in the manuscript that ferritin, liver iron concentration and cardiac T2* are not interchangeable and identify partly different populations. They are retained as alternative routes because each independently establishes a transfusional iron burden justifying chelation and because requiring MRI in every candidate would restrict access without informing the primary outcome. Which criterion was met is recorded and reported for each participant, so that the reader can see which scenario each participant represents.

We should be candid about the trade-off the reviewer is pointing to. The alternative he proposes — requiring, for example, > 20 transfusions and a high liver iron concentration — would define a more homogeneous and more convincingly overloaded population. Applied to a single centre recruiting ten evaluable patients who must also be chelation-intolerant and dialysable, it would make the study unfeasible. We have therefore chosen the more robust ferritin definition plus complete descriptive reporting of the transfusion and end-organ data, and we agree that a future efficacy trial, where the endpoint is clinical rather than mechanistic, should define its population on end-organ burden as the reviewer suggests.

> **Change in the manuscript.** Inclusion criteria; 'Rationale for the eligibility criteria' (Definition of iron overload).

> ***Major 3.*** *Concomitant treatment excluded, please define: at the time of the procedure week, acceptable, but excluding all the patients on Rx will be not wise and fair.*

Response. This criterion has actually been amended in the last version and is now defined more precisely. The amended criterion excludes azacitidine (alone or in combination) or another chemotherapy administered on a repeated or monthly schedule for acute leukaemia, myelodysplastic syndrome or myelofibrosis. Patients treated with a JAK1/2 inhibitor may be included provided the treatment was introduced at least three months before inclusion and the dose has not been modified in the four weeks preceding inclusion — that is, provided the patient is in a stable haematological state. Luspatercept and erythropoiesis-stimulating agents remain excluded.

The reason for the residual exclusions is now stated, and it is interpretative rather than one of safety. Repeated-schedule cytotoxic agents alter erythropoiesis, transfusion requirement and iron kinetics over exactly the window of the intervention and its 90-day follow-up, and with ten evaluable patients that variability cannot be separated from the effect of the device. Luspatercept and erythropoiesis-stimulating agents are retained on the same ground, and we would argue the ground is strongest for them: both act directly on erythropoiesis and on the transfusion requirement. We take the reviewer's point that a blanket exclusion of all treated patients would be neither wise nor fair; that is why the criterion now admits patients on stable background JAK1/2 inhibition, and the exclusions are confined to agents acting on the measured pathway itself. Ongoing treatments and any change during follow-up are recorded and reported.

> **Change in the manuscript.** Exclusion criteria; 'Rationale for the eligibility criteria' (Concomitant antineoplastic treatment).

> ***Major 4.*** *Why are allo Tx recipient candidates excluded? Any specific reason — these are the patients in urgent need.*

Response. We agree with the reviewer, and the criterion has been amended. In the amended protocol, only patients for whom allogeneic transplantation is planned within one month of inclusion are excluded; having an indication for transplantation is no longer in itself an exclusion criterion. The residual one-month window is a matter of interference: conditioning blocks erythropoiesis and abruptly changes iron handling, and the transplant admission would overlap the follow-up window, so a patient transplanted within days of the cycle would contribute uninterpretable follow-up data. Transplant candidates as a group are now eligible, which we agree is the population in which extracorporeal iron depletion is of greatest potential interest.

> **Change in the manuscript.** Exclusion criteria; 'Rationale for the eligibility criteria' (Allogeneic transplantation).

Major 5. Share your expectation for iron removal for each session and add a proposed analysis for a standard candidate with a certain ferritin and labile iron.

Response. This was a valuable request. A new subsection, 'Expected magnitude of iron extraction', states an explicit a priori expectation so that the observed result can be judged against a prespecified benchmark rather than post hoc. The worked example the reviewer asks for is given explicitly. For a 70 kg patient with serum ferritin 2,500 µg/L, transferrin saturation 90% and an estimated NTBI of 5 µmol/L (0.28 mg/L), assuming complete equilibration of the effluent with plasma NTBI, the expected extraction is 2 L × 0.28 mg/L ≈ 0.56 mg per session, or approximately 1.7 mg over the three-session cycle. Under a more conservative assumption of 50% equilibration — plausible with the standard-porosity paediatric dialyser used here — the expected values are approximately 0.28 mg per session and 0.8 mg per cycle.

We then state plainly what these figures mean, which also addresses the Editor's request that efficacy language be toned down. The purpose of the trial is to establish that the device removes a quantifiable mass of the NTBI species that mediate iron toxicity, and to characterise safety, in a deliberately conservative configuration — not to demonstrate a reduction in iron stores. Extraction is expected to scale with membrane surface area, porosity and dialysate flow, and those are the parameters a subsequent dose-finding study would address. We also prespecify a failure threshold: a recovered mass below 0.1 mg per session, or below the limit of quantification after blank subtraction, would be interpreted as a failure of the mechanistic proof-of-concept in this configuration.

Change in the manuscript. New subsection 'Expected magnitude of iron extraction'; Abstract; Strengths and limitations bullet 4.

Minor comments

Minor 1. P8L42 define chelation failure by reference, exact time and agent used.

Response. Chelation failure or intolerance is now defined explicitly in the inclusion criteria as a stable (≥80% of baseline) or increasing ferritin level after at least 6 months of continuous treatment at the maximum tolerated dose.

Change in the manuscript. Inclusion criteria, third criterion; 'Rationale for the eligibility criteria' (Chelation failure or intolerance).

Minor 2. P8L53 Capacity, needs rewording.

Response. Reworded. The criterion now reads: 'Able to understand the study information and to give written informed consent personally.'

Change in the manuscript. Inclusion criteria, sixth criterion.

Minor 3. P8L48 … use not G but metrics, 10e9/L.

Response. Corrected throughout. All platelet counts are now expressed in $\times 10^9$/L — in the inclusion criteria, in the heparin dosing rule, and in the pre-session transfusion rule.

Change in the manuscript. Inclusion criteria; Interventions (anticoagulation); Additional treatments.

Minor 4. Why are sessions 200 mins, please explain.

Response. Explained in the new 'Rationale for the dialysis prescription' subsection. The duration is the quotient of the fixed dialysate volume and the dialysate flow rate: 2,000 mL ÷ 600 mL/h = 3 h 20 min. It is the time required to deliver the entire prepared volume of MEX-CD1-containing dialysate exactly once, in

single-pass mode without recirculation. The reference to '4 hours' elsewhere in the submitted manuscript was an error and has been corrected.

Change in the manuscript. Interventions; new subsection 'Rationale for the dialysis prescription' (Session duration); Feasibility outcomes.

Minor 5. *Why a dense program like HD sessions, not every week? Any scientific, regulatory or ethical reason, please clarify.*

Response. Explained in the same new subsection, with three converging reasons and an explicit statement that none of them is regulatory:

Biological. The plasma NTBI pool is replenished from tissue and macrophage stores within hours of its removal. Closely spaced sessions allow the kinetics of that replenishment, and any cumulative effect across sessions, to be observed within a single physiological state. Weekly sessions would be separated by intervening transfusions, which would confound the within-patient comparison that the primary outcome depends on.

Monitoring. A one-week cycle keeps the entire intervention within a single closely supervised observation window, which the sponsor and the ethics committee considered appropriate for a first exposure of this population to the device.

Change in the manuscript. New subsection 'Rationale for the dialysis prescription' (Session spacing).

We hope that these revisions address the Editor's and the reviewers' concerns, and we thank them again for the time and care they have devoted to this protocol.

VERSION 2 - REVIEW

**Reviewer**	**1**
**Name**	**Słomka, Artur**
**Affiliation**	**Nicolaus Copernicus University, Collegium Medicum, Department of Pathophysiology**
**Date**	**22-Sep-2026**
**COI**	

The authors have addressed my remarks in a thorough and satisfactory fashion.

**Reviewer**	**2**
**Name**	**Arat, Mutlu**
**Affiliation**	**Grup Florence Nigtingale Hospital Turkey, Turkey**
**Date**	**03-Oct-2026**
**COI**	

Acceptable in current version. Changes and comments are well restructured.

VERSION 2 - AUTHOR RESPONSE

Reviewer 1 (Dr Artur Słomka): The authors have addressed my remarks in a thorough and satisfactory fashion.

Response: We sincerely thank Dr Słomka for his careful review and constructive comments, which substantially improved the manuscript.

Reviewer 2 (Dr Mutlu Arat): Acceptable in current version. Changes and comments are well restructured.

Response: We sincerely thank Dr Arat for his positive assessment and his contribution to improving the manuscript
